# Selective Chemo‐Divergent Hydrogenation of Ethylene Carbonate Enabled by Multi‐Functional Poly(Ionic Liquids)‐Stabilized Ru Nanoparticles

**DOI:** 10.1002/anie.202507548

**Published:** 2025-06-25

**Authors:** Wenjuan Wang, Thierry Tassaing, Joan Vignolle

**Affiliations:** ^1^ University of Bordeaux, CNRS, Bordeaux INP, Laboratoire de Chimie des Polymères Organiques (LCPO), UMR 5629 16 Av. Pey Berland Pessac cedex 33607 France; ^2^ University of Bordeaux, CNRS, Bordeaux INP, ISM UMR 5255 351 Cours de la Libération Bordeaux Talence Cedex 33405 France

**Keywords:** Chemo‐divergent, Cyclic carbonates, Poly(ionic liquid)s, Ruthenium nanoparticles, Selective hydrogenation

## Abstract

Cyclic carbonates, in particular ethylene carbonate (EC), are pivotal compounds across chemical sciences because of their unique properties. Although, their transformation into valuable products has attracted great attention, efficient and selective transformations remain challenging. In this work, we report a catalytic system composed of Ru nanoparticles (RuNPs) stabilized by poly(ionic liquids) (Ru@PIL), that enables the selective chemo‐divergent hydrogenation of EC into either EtOH and CO_2_ or EG and CH_4_, under solvent‐free conditions. Those transformations relied on the multi‐task ability of poly(ionic liquids) (PILs), which provides efficient electro‐steric protection of the NPs, good solubility in neat carbonates, and organocatalytic activity depending on the nature of the PIL counter‐anions. Hence, PIL incorporating nucleophilic anions, such as I^−^, triggers the cascade transformation of EC into EtOH via a sequential decarboxylation‐hydrogenation process. Conversely, in the presence of non‐nucleophilic anions, the PIL is catalytically a spectator, yielding to the “direct hydrogenation” of EC by the RuNPs.

## Graphical abstract

A highly efficient and selective chemo‐divergent hydrogenation of cyclic carbonates is promoted by in situ generated poly(ionic liquid)‐stabilized ruthenium nanoparticles (Ru@PIL) catalysts. Under solvent‐free conditions, ethylene carbonate is transformed into either ethylene glycol and CH_4_ or EtOH and CO_2_, following a “direct hydrogenation” or a “hydrogenative decarboxylation” pathway, respectively.

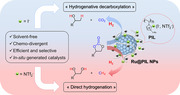

Five‐membered cyclic carbonates, such as ethylene carbonate (EC), are primarily recognized as green solvents and electrolytes for batteries due to their desirable physicochemical properties, including high boiling points, high polarity, and hydrolytic stability.^[^ ^1^, ^2^ ^]^ These compounds have also garnered increased interest in various fields, including synthetic organic chemistry, material science, and CO₂ valorization, owing to their versatility.^[^ ^2^, ^3^ ^]^ In these applications, carbonates serve as genuine reagents or substrates that can undergo various transformations, primarily: (i) decarboxylation, (ii) hydrolysis or transesterification, and (iii) hydrogenation (Scheme 1). The decarboxylation of five‐membered ring cyclic carbonates is an endergonic process with a high energy barrier, typically requiring high temperatures and a catalyst.^[^ ^4^ ^]^ This process offers a safe method for producing epoxides, such as ethylene oxide (EO) (Scheme 1a). Industrially, EC is a key intermediate in the Shell–Omega process, where it is hydrolyzed into ethylene glycol (EG) with the concomitant release of CO₂ (Scheme 1b).^[^ ^5^, ^6^, ^7^ ^]^

**Scheme 1:**
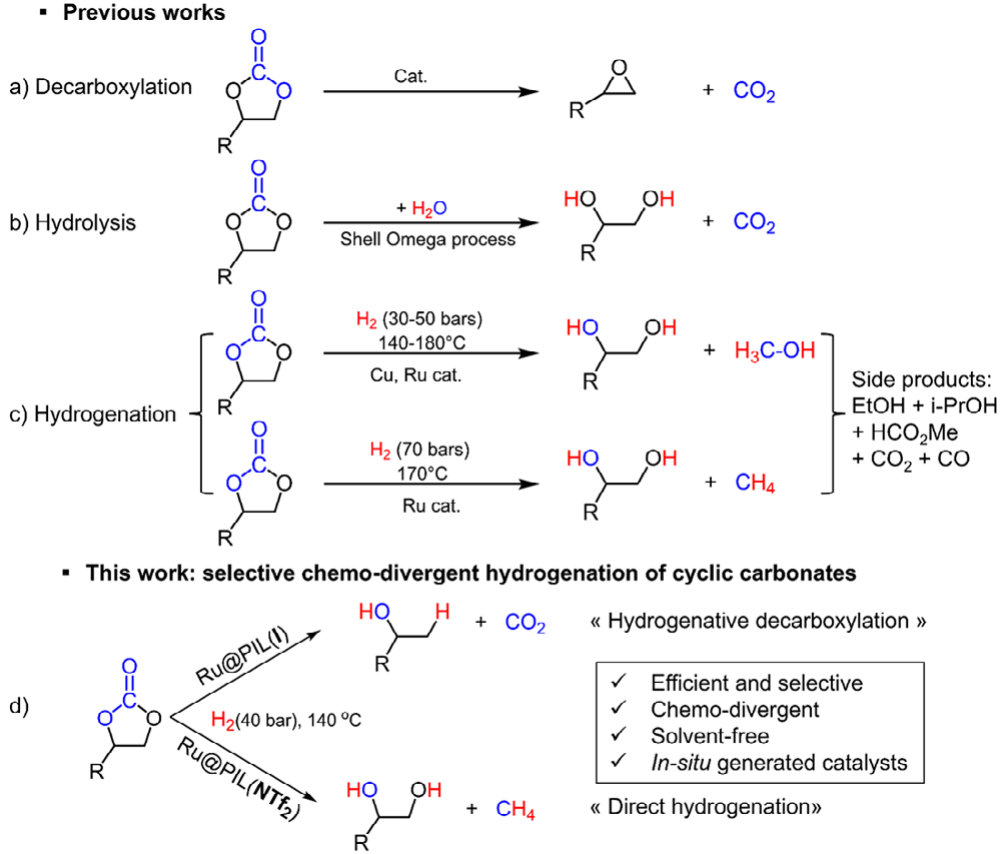
a) Decarboxylation, b) hydrolysis of EC (R = H), c) previous contributions on the hydrogenation of EC into MeOH and glycols and hydrogenation of EC into EG and CH_4_ and d) this work on the selective chemo‐divergent hydrogenation of EC mediated by Ru@PIL(X) NPs (X = I^−^ or NTf_2_^−^). NTf_2_^:^ bis‐(trifluoromethyl)sulfonylimide.

The hydrogenation of EC has attracted considerable attention in recent years as it provides an indirect, yet highly selective, pathway for the transformation of CO₂ into valuable chemicals such as EG and MeOH (Scheme 1c).^[^ ^8^, ^9^, ^10^, ^11^, ^12^, ^13^, ^14^, ^15^, ^16^, ^17^, ^18^, ^19^, ^20^ ^]^ Besides EC, various carbonates and carbamates have been considered promising activated CO₂ sources, enabling more selective transformations under moderate reaction conditions.^[^ ^8^, ^9^, ^21^, ^22^ ^]^

Initially, homogeneous Ru‐based catalysts demonstrated efficacy in this reaction,^[^ ^8^, ^13^, ^14^, ^15^, ^16^, ^17^, ^18^, ^19^, ^20^ ^]^ but the development of heterogeneous systems, particularly those based on Cu, has also been reported.^[^ ^8^, ^14^, ^15^, ^16^, ^17^, ^18^, ^19^ ^]^ In contrast to homogeneous systems, Ru nanoparticle (RuNP)‐based catalysts have shown the ability to selectively hydrogenate EC to produce EG and CH₄ (Scheme 1c),^[^ ^23^, ^24^ ^]^ thereby avoiding the energy‐intensive separation of MeOH and diols. Notably, the hydrogenation of carbonates can yield small amounts of higher alcohols, such as EtOH and isopropanol (iPrOH), or oligo(glycols), as well as methyl formate, CO_2_ and CO as side products,^[^ ^23^ ^]^ resulting from the competition between different reaction pathways (Scheme 1c). This complexity also highlights the synthetic potential of cyclic carbonates as versatile precursors for various liquid fuels and chemicals under hydrogenative conditions.

To fully exploit carbonates as sources of specific gaseous and liquid products, it is essential to develop highly active and selective catalysts that operate under moderate and solvent‐free conditions, thereby enhancing sustainability. While the chemo‐divergent^[^ ^25^ ^]^ selective hydrogenation of cyclic carbonates represents a significant challenge, as it requires precise control over reaction pathways to direct the formation of desired products, it would enable the formation of different valuable liquid products, such as EtOH, iPrOH, MeOH, EG, and propylene glycol (PG), from a single substrate‐catalyst system.

In the context of CO₂ transformations, ionic liquids (ILs) have emerged as versatile, non‐volatile solvents^[^ ^26^, ^27^ ^]^ and stabilizers for nanoparticles (NPs),^[^ ^28^, ^29^, ^30^ ^]^ not only due to their capability to solubilize CO_2_ but also because of their tailorable properties.^[^ ^26^, ^27^, ^28^, ^29^, ^30^ ^]^ ILs are also known to catalyze organocatalytic transformations, such as the cycloaddition of CO₂ with epoxides,^[^ ^31^, ^32^ ^]^ facilitated by the nucleophilicity or basicity of their anions and the hydrogen‐bond‐donating ability of their cations.^[^ ^32^, ^33^ ^]^ Moreover, ILs have enabled the development of highly effective NP‐based catalysts for CO₂ valorization,^[^ ^34^ ^]^ thanks to their tunable properties derived from the virtually limitless combinations of cations and anions.

Poly(ionic liquids) (PILs),^[^ ^35^, ^36^, ^37^, ^38^ ^]^ the polymeric analogs of IL, offer additional advantages due to their macromolecular structure, which enhances the steric stabilization of NPs^[^ ^39^, ^40^, ^41^ ^]^ and allows for tailored solubility in various solvents.^[^ ^42^, ^43^ ^]^ Their properties can be fine‐tuned via anion exchange reactions,^[^ ^35^, ^36^, ^37^, ^38^, ^42^, ^43^ ^]^ providing a versatile platform for catalytic applications.^[^ ^44^, ^45^ ^]^ We have recently demonstrated that such anion metathesis could reversibly modulate the activity and selectivity of poly(imidazolium)‐stabilized RuNPs (Ru@PIL NPs) in the hydrogenation of unsaturated substrates.^[^ ^46^ ^]^ This ability to control catalytic performance “on demand”^[^ ^47^ ^]^ positions PIL‐stabilized catalysts as promising candidates for achieving other selective hydrogenation reactions.

Herein, we report that the solvent‐free, selective, and chemo‐divergent hydrogenation of EC can be achieved through the multifunctionality of PIL‐stabilized RuNPs (Figure 1). Notably, the nature of the PIL counter‐anion plays a pivotal role in directing the reaction pathway, enabling the selective hydrogenation of EC to either ethylene glycol (EG) and methane (CH₄) or ethanol (EtOH) and carbon dioxide (CO₂) (Scheme 1d). This selective chemo‐divergent hydrogenation reaction arises primarily from the nucleophilic or spectator role of the PIL counter‐anion, coupled with the hydrogenating ability of the Ru surface.^[^ ^48^ ^]^

**Figure 1:**
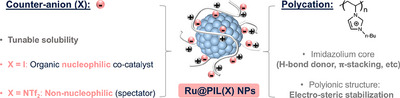
Multi‐functionality of PILs (in grey) and catalytic properties of Ru@PIL(X) NPs depending on the nature of X^−^ counter‐anion (in pink).

We recently reported that the chemo‐selective hydrogenation of styrene into ethylbenzene could be achieved quantitatively by using Ru@PIL(I), with I‐ as PIL counter‐anions.^[^ ^46^ ^]^ Based on solid state and solution characterizations,^[^ ^46^ ^]^ this chemo‐selectivity was proposed to arise from the peculiar PIL‐Ru interaction that prevents styrene from binding to the faces of RuNPs via the aromatic moiety. Hence, only the exocyclic C═C bond can access the active sites located on edges and corners. We thus hypothesized that the constrained access to the faces of RuNPs might also be relevant for the selective hydrogenation of cyclic carbonates. Thus, our preliminary investigations started with ex situ prepared Ru@PIL(I) NPs of 1–2 nm according to TEM analysis (Figure S1). X‐ray photoelectron spectroscopy (XPS) analysis revealed that while Ru(0) was the main oxidation state (95%), some oxidized Ru was also present on the surface (Figure S2).

The solvent‐free hydrogenation of EC was thus investigated at 140 °C, under 40 bars of H_2_ (standard conditions), with 1 mol% of Ru@PIL(I). After 2 4 h, 94% conversion of EC was observed by ^1^H NMR analysis of the liquid phase (Table 1, entry 1). To our surprise, and in contrast to the selectivity reported in the literature with RuNP‐based systems,^[^ ^23^, ^24^ ^]^ neither MeOH nor EG could be identified in this transformation. Instead, EtOH was produced in high yield (94%). Furthermore, analysis of the gas phase revealed an almost complete selectivity for CO_2_ (98%).

**Table 1:** Hydrogenation of EC by different catalysts.^a)^

			Liquid prod. (%)^b)^			Gas prod. (%)^c)^	
Entry	PIL(X) stabilizer	Conv. (%)^b)^	EtOH	EG	MeOH	CH_4_	CO_2_
1^d)^	PIL(I)	94	94	0	0	2	98
2^e)^	PIL(I)	99.9	95	5	0	4	95
3^e)^	PIL(Br)	33	3	31	0	30	64
4	PIL(Cl)	99.9	<1	>99	35	69	31
5^f)^	PIL(NTf_2_)	99.7	2	95	5	73	27

^a)^ Reaction conditions: 3 mmol EC, (1 mol% Ru(II) (COD)(Meallyl)_2_ and 3.5 mol% PIL), H_2_ (40 bar), 24 h, 140 °C.

^b)^ The conversion of EC and the yield of alcohols are calculated based on ^1^H NMR spectrum, mesitylene as the internal standard, and as the external standard in the case of PIL(NTf_2_).

^c)^ The selectivity of each gas is determined by FT‐IR spectra.

^d)^ Ex situ prepared Ru@PIL(I) NPs.

^e)^ 1% of CO was also generated.

^f)^ 2 mol% of Ru and 7 mol% PIL(NTf_2_) were used.

The unexpected formation of EtOH is believed to follow a two‐steps mechanism,^[^ ^49^ ^]^ involving the PIL(I)‐catalyzed decarboxylation of EC to generate EO and CO_2_ in the first step (Scheme S2), by analogy with the known catalytic activity of PIL for the reverse reaction, i.e., the cycloaddition of CO_2_ and epoxides,^[^ ^32^, ^50^ ^]^ and in line with the recent work of Wang, Liu et al. on the decarboxylation of cyclic carbonates catalyzed by porous PIL.^[^ ^4^, ^51^ ^]^ Whereas this reaction is thermodynamically unfavorable, the coupling of this decarboxylation step with the highly exergonic hydrogenation of EO into EtOH in the second step^[^ ^52^ ^]^ would push the overall transformation toward the formation of EtOH (Figure S16 and Table S4). Furthermore, the high yield of EtOH may also suggest an efficient cascade process due to the proximity of both nucleophilic and metallic active sites. Hence, the high activity and selectivity of Ru@PIL(I) observed in this transformation remarkably highlights the multiple functions of PILs, for solubilizing the nano‐catalyst in neat EC, for efficiently stabilizing the RuNPs, for activating (via H‐bonding) and catalyzing, via the I^−^ counter‐anion,^[^ ^4^ ^]^ the decarboxylation of EC in the vicinity of the Ru surface. The high selectivity for EtOH is also favored by the endergonic decarboxylation step of EC, which prevents the accumulation of EO and hence the formation of higher oligo‐glycols (see GC/MS, Figure S5).

Given the key role of PIL counter‐anions in this transformation, it was of interest to investigate different counter‐anions. Furthermore, to avoid the excessive oxidation of ex situ Ru@PIL(I) NPs (Figure S3) and facilitate the screening of the experimental conditions, the in situ generation of Ru@PILs NPs was next explored in neat EC. However, instead of using RuX_3_ (X = Cl, Br), we turned our attention to halide‐free Ru precursors, such as [Ru^II^ (COD)(Meallyl)_2_] and [Ru^0^(COD)(COT)], that only generate alkanes upon hydrogenation, while avoiding the generation of potentially detrimental HX (X = Cl, Br) under H_2_ pressure.

Thus, the solvent‐free hydrogenation of EC was then investigated with in situ generated RuNPs from commercially available Ru^II^ precursor and different counter‐anions at 140 °C, under 40 bars of H_2_ for 24 h (Table 1). The molar ratio between PILs and Ru was set to 3.5 to target a metal loading of 10 wt%.

By analogy with the reactivity of ex situ prepared Ru@PIL(I) NPs, the combination of PIL(I) and the Ru^II^‐complex led to the quantitative conversion of EC (99.9%) with 1 mol% of Ru catalyst (Table 1, entry 2). Furthermore, the liquid phase was mainly composed of EtOH (95%) with a small amount of EG (5%), according to ^1^H NMR (Figure S4) and gas chromatography mass spectrometry (GC‐MS, Figure S5). As expected, the gas phase was mainly composed of CO_2_ (95%) resulting from the decarboxylation of the carbonate (Figure S14), with traces of CH_4_ (4%). As anticipated from the lower nucleophilicity of Br^−^, in situ generated Ru@PIL(Br) resulted in a much lower EC conversion (33%), with the liquid phase being composed of EG and EtOH as the main (31%) and minor products (3%), respectively (Table 1, entry 3). Unexpectedly, PIL(Cl) led to a much more active catalyst than Ru@PIL(Br), despite chloride being the least nucleophilic counter‐anion of the halogen series (full EC conversion, Table 1, entry 4). However, it is worthy to note that the selectivity of the hydrogenation was completely and selectively switched from EtOH to EG in this case. In the gas phase, the moderate 70:30 CH_4_/CO_2_ selectivity observed likely originated from the incomplete hydrogenation of MeOH (35%) that is coproduced with EG during the hydrogenation of EC (Figure S7). As expected, a similar selectivity for EG was observed with in situ generated Ru@PIL(NTf_2_) catalysts, featuring NTf_2_^−^ as non‐nucleophilic counter‐anions. Using 2 mol% of Ru precursor (Table 1, entry 5), full EC conversion and high yield of EG could be obtained, with minute amounts of EtOH (2%) and MeOH (5%) (see Table S3 for optimization study).

Those results highlight that, depending on the nucleophilicity of the PIL(X) counter‐anions, Ru@PIL(X) could either behave as a mono‐functional catalyst, where the Ru surface promotes the “direct hydrogenation” of EC into EG and CH_4_, or as a bifunctional organic‐metallic hybrid catalyst that enable the hydrogenation of EC into EtOH and CO_2_ via a sequential decarboxylation‐hydrogenation transformation, with non‐nucleophilic and nucleophilic X^−^ counter‐anions, respectively.

The in situ formation of very small Ru@PIL(X) NPs (X = Cl, Br, I, NTf_2_) of 1.8–2.9 nm could be confirmed by TEM analysis of the crude reaction mixture (Figure 2a,b and ESI), in agreement with the mean size of RuNPs obtained by the ex situ polyol process (ESI). Analysis of those in situ generated RuNPs by XPS confirmed the formation of Ru^0^ as the main oxidation state (461.83 eV) and the presence of some oxidized Ru at 461.4 eV (Figure 2a,b and Table S2), likely originating from the preparation of the sample in air.

**Figure 2:**
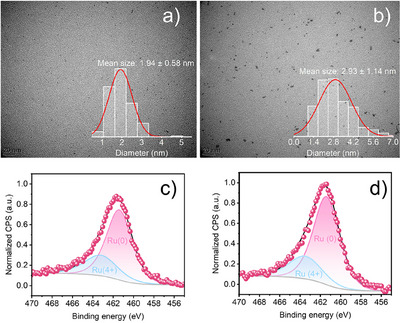
TEM images of a) in situ synthesized Ru@PIL(I) and b) Ru@PIL(NTf_2_) and their size distributions (embedded figure). c) XPS spectrum of Ru3p for Ru@PIL(I) and d) Ru@PIL(NTf_2_).

Finally, to highlight the interest of PIL compared to classical PVP stabilizer, the hydrogenation of EC was performed using in situ generated nano‐catalysts (Table S3). Under the standard conditions, 45% EC conversion and 62% selectivity for CH_4_ were obtained. Note that the liquid phase was composed of 43% of EG and 1.6% of EtOH, evidencing a lower activity and selectivity of Ru@PVP NPs. In the case of commercially available Ru/C, low EC conversion (17%) was obtained, further evidencing the superior catalytic and tunable properties of Ru@PIL systems.

Given the high selectivity for either EG or EtOH observed for the chemo‐divergent hydrogenation of EC with in situ generated Ru@PIL(X) NPs, with X = I^−^ and NTf_2_^−^, the solvent‐free hydrogenation of various carbonates was investigated under the standard reaction conditions. The scope of these transformations was first studied in the case of the hydrogenative decarboxylation cascade catalyzed by Ru@PIL(I) (Table 2).

**Table 2:** Hydrogenative decarboxylation of cyclic carbonates mediated by Ru@PIL(I).^a)^

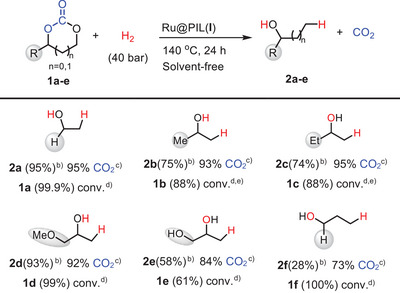

^a)^ Reaction conditions: 3 mmol substrates, 1 mol% Ru(II) (COD)(Meallyl)_2_, 3.5 mol% PILs(I).

^b)^ Yield of the major “branched” alcohol isomers; 10%–15% of linear alcohol isomers were also formed; see Table S4 for details.

^c)^ The CO_2_ selectivity was determined by FT‐IR analysis.

^d)^ The conversion of EC was calculated by ^1^H NMR analysis using mesitylene as internal standard.

^e)^ The reaction was performed at 160 °C.

High conversions were obtained for both propylene carbonate (**1b**) and butylene carbonate (**1c)**, affording good yields of isopropanol (**2b**) and 2‐butanol (**2c**), respectively with high selectivity toward CO_2_ (93%–95%). Similarly, **1d** underwent quantitative conversion to afford **2d** in high yield (93%), with high selectivity of CO_2_ (92%). Interestingly, glycerol carbonate (**1e**) also proved to be a suitable substrate, albeit a moderate conversion (61%) and yield of glycerol (**2e**, 58%) were obtained. All those transformations involving unsymmetrical cyclic carbonates occurred with fairly good regioselectivity in favor of the “branched” alcohol, the linear isomer being obtained in 10%–15% yield (Table 2, ESI). Finally, six‐membered rings, such as trimethylene carbonate (TMC, **1f**), could not undergo selective hydrogenative decarboxylation, which most likely reflects the inability of PIL(I) to catalyze the formation of the corresponding oxetane under those experimental conditions.

The “direct hydrogenation” of the same cyclic carbonates into diols and CH_4_ was next investigated with in situ generated catalyst Ru@PILs(NTf_2_) (Table 3). Gratifyingly, all five‐membered cyclic carbonates could be hydrogenated with high conversions (78%–100%), resulting in high yields of the corresponding diol (73%–100%), even in the case of glycerol carbonate (99% conv. and 99% yield). Hydrogenation of those carbonates also produced methane with high selectivity (73%–83%). Finally, hydrogenation of TMC provided only a low yield of 1,3‐butanediol despite the high conversion observed, suggesting the coexistence of multiple reaction pathways, regardless of the Ru@PIL(NTf_2_) catalyst used.

**Table 3:** Direct hydrogenation of different cyclic carbonates by in situ generated Ru@PIL(NTf_2_).^a)^

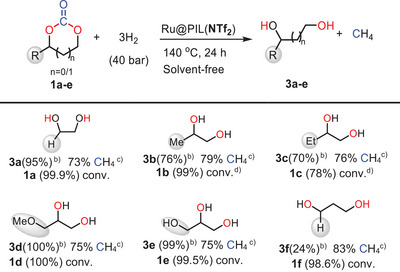

^a)^ Reaction conditions: 3 mmol substrates, 2 mol% Ru(II) (COD)(Meallyl)_2_, 7 mol% PIL(NTf_2_).

^b)^ The conversion of EC and the yield of diols are calculated based on ^1^H NMR analysis with mesitylene as external standard.

^c)^ The gas selectivity was determined by FT‐IR analysis.

^d)^ The reaction was performed at 160 °C.

In conclusion, this work demonstrates that PILs can behave as multi‐functional stabilizers of RuNPs that enable the selective chemo‐divergent hydrogenation of EC. Hence, depending on the nature of the PIL counter‐anion, either the “direct hydrogenation” or the “hydrogenative decarboxylation” of carbonates could be selectively performed, yielding either EG and CH_4_ or EtOH and CO_2_, respectively. The latter transformation was proposed to proceed via the initial PIL‐catalyzed decarboxylation of EC, followed by the hydrogenation of the ensuing epoxide by the RuNPs. The spatial proximity of these catalytic sites allows this cascade transformation to proceed with high efficiency and selectivity. Importantly, not only EC but also various five‐membered ring cyclic carbonates proved suitable for this transformation using in situ prepared catalysts, under solvent‐free conditions.

Given the pivotal role of cyclic carbonates in CO_2_ related transformations and more generally in synthetic chemistry, we believe that the design of such selective chemo‐divergent strategies could be of high interest to generate diversity from common resources. Moreover, the multi‐functionality of PILs arising from the virtually infinite combination of poly(cations) and anions could lead to new cascade‐type transformations that we are currently investigating.

## Conflict of Interests

The authors declare no conflict of interest.

## Supporting information



Supporting Information

## Data Availability

The data that support the findings of this study are available from the corresponding author upon reasonable request.
