# Supplementary material for: Selective Chemo‐Divergent Hydrogenation of Ethylene Carbonate Enabled by Multi‐Functional Poly(Ionic Liquids)‐Stabilized Ru Nanoparticles
Source: Angew Chem Int Ed Engl. 2025 Jun 25;64(36):e202507548. doi: 10.1002/anie.202507548 (PMC12402846; doi:10.1002/anie.202507548)
Supplement: Supplementary file 1 — Supporting Information [file ANIE-64-e202507548-s001.docx]

**Selective Chemo-divergent Hydrogenation of Ethylene Carbonate Enabled by Multi-functional Poly(ionic liquids)-stabilized Ru Nanoparticles**

Wenjuan Wang, ^[a,b]^ Thierry Tassaing, ^[b]^*Joan Vignolle ^[a]^*

DOI: 10.1002/anie.2024XXXXX

**Table of Contents**

| General data. | S1-3 |
| --- | --- |
| **Scheme S1** and **Table S1** Synthesis of PILs and Ru@PIL(I) | S3-6 |
| **Figure S1** TEM images and size distribution of *ex-situ* and *in-situ* generated Ru@PIL(X) | S7 |
| **Figures S2-S3 and Table S2** XPS and TGA analysis of the isolated Ru@PIL(I). | S8-9 |
| General procedure for the hydrogenation of ethylene carbonate by Ru-based catalysts. | S10 |
| **Table S3** Screening of the reaction conditions hydrogenolysis of EC under solvent-free conditions. | S11-12 |
| **Figure S4:** Qantification and identification of the liquid phase of the hydrogenation of EC catalyzed by in-situ prepared Ru@PIL(I). | S13-14 |
| **Figures S5:** GC-Mass spectrum of the liquid phase of the hydrogenation of EC catalyzed by in-situ prepared Ru@PIL(I). | S15 |
| **Figures S6-S8** Qantification and identification of the liquid phase of the hydrogenation of EC catalyzed by in-situ synthesized Ru@PIL(Y), (Y= Br,Cl, NTf_2_). | S16-18 |
| **Figures S9-S11** GC-Mass spectrum of the liquid phase of the hydrogenation of EC catalyzed by Ru@PIL(Y), (Y= Br, Cl, NTf_2_) generated in-situ. | S19-20- |
| **Figures S12-S15** Qantification and identification of the gas phase of the hydrogenation of EC. | S21-24 |
| **Figure S16** In-situ IR monitoring test of the reaction of EC and PIL(I) under N_2_. | S25 |
| **Figures S17-S18 and Table S4** Hydrogenation of PO by in-situ prepared Ru@PIL(I). | S26-27 |
| **Scheme S2** Proposed mechanisms of hydrogenation of EC by Ru@PIL(I) generated in-situ. | S28 |
| **Scheme S3** Proposed mechanisms of hydrogenation of EC by Ru@PIL(NTf_2_) generated in-situ. | S29 |
| **Tables S5 and S6 (All information of Tables 2 and 3)** | S30-31 |
| **Figures S19-S28** ^1^H NMR spectrum and GC-MS of different substrates in Table 2 | S32-38 |
| **Figures S29-S39** ^1^H NMR spectrum and GC-MS of different substrates in Table 3 | S39-44 |
| **References** | S45-46 |

**Experimental**

**General Data**

1-Vinyl-imidazole, 1-chlorobutane, 1-bromobutane, 1-iodobutane, and Poly(vinylpyrrolidone) (MW 55000 g/mol) (PVP) were purchased from Sigma Aldrich, France. Ruthenium chloride hydrate (RuCl_3_•xH_2_O, 99.9%), Ru(0) (COD)(COT) and Ru(II) (COD)(Meallyl)_2_ were purchased from Strem Chemicals. Lithium bis(trifluoromethanesulfonimide) (LiNTf_2_) and ethylene carbonate (EC) were purchased from TCI France. Ru on Carbon (Ru/C, 5 wt%) was purchased from Alfa Aesar France and used as received. HPLC grade solvents were used for synthesis, purification, and analysis. All reagents and solvents were used without further purification unless otherwise mentioned.

**^1^H NMR** spectra were recorded at 25 °C with a Bruker Avance 400 MHz. All the chemical shifts are reported in parts per million (*δ*, ppm) and are calibrated using the residual proton in the deuterated solvent (DMSO-*d*_6_ at 2.50 ppm ^1^H NMR, Acetone-d_6_ at 2.05 ppm ^1^H NMR, isopropanol-d_8_ at 5.10 and 1.09 ppm ^1^H NMR).

**Transmission Electron Microscopy (TEM)**: The particle sizes of RuNPs were determined by recording the TEM images using a Hitachi 7650 TEM operating at 80 kV in high-resolution mode. The samples for TEM analyses were prepared by putting a drop (10 μL) of NP solution (0.5 mg/mL in methanol) on a carbon coated copper grid and drying it for 90 minutes before analysis. The particle size and distribution were determined by measuring 200 particles at random locations using Nano Measurer 1.2 software.

**X-ray Photoelectron Spectroscopy (XPS)**: XPS analysis was carried out on PIL-X and Ru@PIL(X) to study the nature of the interaction between PILs and RuNPs. K-Alpha X-ray Photoelectron Spectrometer from Thermo Fisher Scientific equipped with monochromatized AlKα source (h*ν*=1486.6 eV) was used for surface analysis. The full spectra (0-1350 eV) and high-resolution

spectra were recorded with a constant pass energy of 200 eV and 40 eV respectively. Ar+ sputtering was used for depth profiles. High-resolution spectra were processed and fitted with AVANTAGE software (Version 6.8.0) from Thermo Fisher Scientific. All the scans were corrected considering C(1s) as the reference (284.8 eV).^[1]^ Peaks were assigned according to Moulder et at.^[2]^ and NIST database.^[3]^.

**Thermogravimetric analysis (TGA)** was recorded by TGA Q-500 from TA instruments to quantify the Ru content of all NP samples.

**Fourier transform infrared (FTIR)** spectra were measured by FT-IR Spectrometer Bruker (NICOLET 6700). Data analysis was done with OMNIC software.

**Gas chromatography mass spectrometry** (**GC-MS,** EI mode): The GC–MS analysis was performed by the CESAMO on a Thermo Trace GC ultra gas chromatograph coupled to a Thermo ISQ mass detector. Capillary GC analysis was performed on an Optima Wax (30m×0.25mm i.d., 0.25 μm) capillary column (PEG) with helium as carrier gas. GC conditions were 40 ^o^C keeping 4 min, at rate of 15 ^o^C/min to 260 ^o^C, keeping 4 min, carrier gas (He) flow rate of 1.2 mL/min. Injection temperature was at 230 ^o^C with split mode. The transfer line and ion source temperatures were maintained at 250 and 200 ^o^C. In the full-scan mode, electron ionization (EI) mass spectra in the range of 40–800 (m/z) were recorded at 70 eV electron energy.

**Size exclusion chromatography (SEC)**: Molecular weight and polydispersity index of PIL were determined by the procedure reported by Matyjaszewski et. al.^[4]^ A SEC system equipped with PSS SDV Linear S (5 µm) column was used at eluent flow-rate of 1 ml/minute. THF containing 10 mmol/L of LiNTf_2_ was used as eluent. A viscometer, a multi-angle light scattering detector, and a RI detector from Wyatt Technology were used to acquire elution traces. ASTRA 6.1 software was used for the processing of elution traces. Macromolecular characteristics of different PIL are given in Table S1. For SEC sample preparation anion exchange was carried out on all synthesized PILs to achieve bis(trifluoromethyl)sulfonimide (NTf_2_^-^) anion by following our previous work (Scheme S1b).^[5]^ Typically, 4 molars equivalent LiNTf_2_ dissolved in water (10 mL) was added to a 20 ml aqueous solution of **PIL(X)** (**X** is Cl or Br) (10 mg/mL) under stirring for 24 h. For the anion exchange of **PIL(I)**, AgNTf_2_ was used instead of LiNTf_2_. In this case, 2 molars equivalent AgNTf_2_ dissolved in methanol (10 mL) was added to 20 mL methanol solution of **PIL(I)** (10 mg/ml) under stirring for 1 day.

**
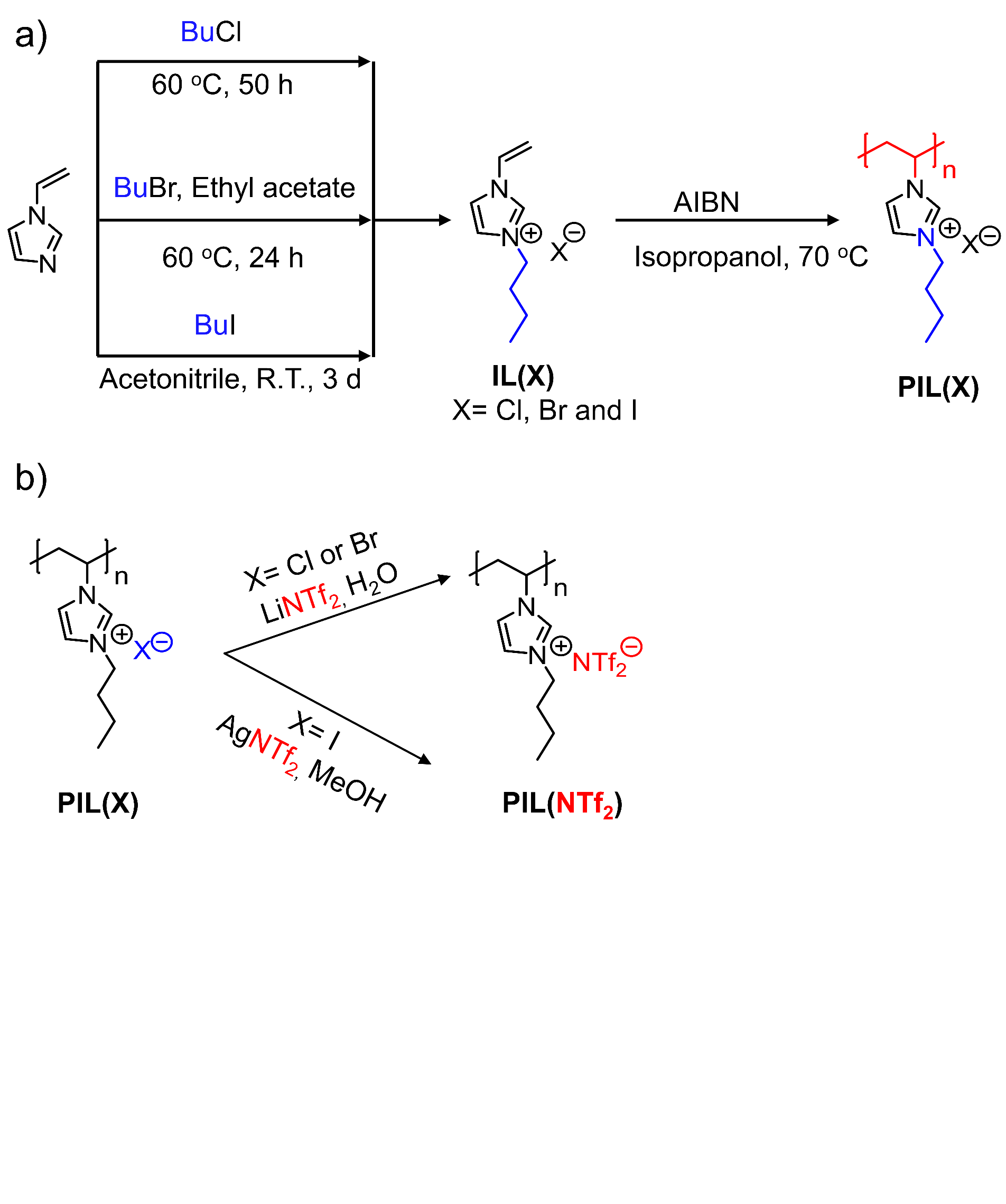
**

**Scheme S1** Synthetic route (a) of **PIL(X)** and the anion exchange (b) of **PIL(X)** with LiNTf_2_ or AgNTf_2_.

**Synthesis of Ionic Liquids (IL):** ^[6]^

Briefly, butyl-3-vinylimidazolium halides (X =Cl, Br, I) were synthesized by the quaternization reaction (Scheme S1a) between 1-vinyl-imidazole and butyl halides reported in the literature.^6a-b^

Synthesis of butyl-3-vinylimidazolium bromide (**IL(Br)**): 1-Bromobutane (BuBr, 99.6 mmol) was added into a flame-dry Schlenk flask (100 mL) containing the ethyl acetate (dry, 30 mL) solution of 1-butyl-1-vinylimidazole (83 mmol) and a stir bar under argon. Then the Schlenk flask was immersed in an oil bath at 60 °C for 18 h. After cooling down to room temperature (R.T.), the upper phrase was poured away, then the lower phrase was dissolved in MeOH and was precipitated three times in cold diethyl ether (Et_2_O) and dried at 60 °C overnight, a light-yellow liquid (**IL(Br)**, 95%) was got.

Synthesis of butyl-3-vinylimidazolium chloride (**IL(Cl)**): In a flame-dry Schlenk flask (100 mL), 83 mmoL of 1-butyl-1-vinylimidazole and a stir bar were added under argon, then 1-chlorobutane (BuCl, 99.6 mmoL) was dropwise added. After that, the Schlenk flask was equipped with a condenser and was immersed in an oil bath at 70 °C under vigorous stirring for 24 h. After cooling down to R.T., most of BuCl was removed under vacuum in a water bath. Afterwards, the mixture left was dissolved in MeOH and was further purified in cold Et_2_O 3 times and dried, affording a white solid of **IL(Cl)** (65%).

Synthesis of butyl-3-vinylimidazolium iodide (**IL(I)**): A flame-dry Schlenk flask (100 mL) was charged with 1-butyl-1-vinylimidazole (83 mmol), dry acetonitrile (20 mL) and a stir bar under argon. Subsequently, 99.6 mmol of 1-iodobutane (BuI) was added. Then the reaction mixture was stirred at room temperature for 3 days. Then the reaction mixture was washed with MeOH and a large amount of cold Et_2_O three times and dried, collecting a yellow liquid (**IL(I)**, 97%).

**Synthesis of Poly(Ionic) Liquids (PIL):** ^[6]^

A 100 mL Schlenk flask was charged with 5 mmol of **IL**, 0.5 mol% of AIBN (azobis(2-methylpropionitrile), the thermal initiator), 20 mL of isopropanol and a stir bar as well. To make the mixture into a homogeneous solution, the Schlenk flask was put on a vibration mixer (shake vortex:10) for 5 min. After 3 freeze–pump–thaw cycles, the free radical polymerization was carried out at 70 °C for 24 h (Scheme S1). Poly(1-butyl-3-vinylimidazolium chloride) (**PIL(Cl)**) and poly(1-butyl-3-vinylimidazolium bromide) (**PIL(Br)**) were precipitated repeatedly in diethyl ether. Poly(1-butyl-3-vinylimidazolium iodide) (**PIL(I)**) was precipitated once in diethyl ether and then taken for dialysis in methanol to remove the remaining **IL(I)** monomer. Finally, **PIL** was analyzed by ^1^H NMR to confirm the absence of any unreacted monomers, and ^1^H NMR spectra of **PIL(Cl)**, **PIL(Br)**, and **PIL(I)** match those reported in the literature.^[6]^

**Table S1**. Molecular weight (Mw and Mn) and polydispersity index (PDI) of synthesized PIL.

| **Entry** | **Sample** | **Mw(g/mol)** | **Mn (g/mol)** | **PDI** |
| --- | --- | --- | --- | --- |
| 1 | PIL(Cl) | 4.717×10^3^ | 6.320×10^3^ | 1.340 |
| 2 | PIL(Br) | 4.015×10^3^ | 6.026×10^3^ | 1.501 |
| 3 | PIL(I) | 6.471×10^3^ | 1.073×10^4^ | 1.659 |
| 4 | PVP | 55000 (Sigma) |  |  |

**Synthesis of Ru@PIL(I):** ^[7]^

RuCl_3_•xH_2_O was used as metal precursors and PIL(I) was used as the stabilizer for the synthesis of PILs-I-stabilized Ru nanoparticles (NPs). The best molar ratio between PIL(I) (IL(I) units) to RuCl_3_•xH_2_O (i.e. 10:1) we found from our previous work was adopted to prepare **Ru@PIL(I)** via polyol process. ^[7]^ Typically, 400 mg of PIL-X and RuCl_3_•xH_2_O were dissolved in 40 ml ethylene glycol in a 200 mL Schlenk flask. After stirring for 4 h at 750 rpm, the homogeneous red-brown solution was degassed with argon and immersed in an oil bath preheated at 170 °C. The red-brown solution slowly turned black, indicating the formation of Ru (0) NPs. After 1.5 h, **Ru@PIL(I)** NPs were collected by centrifuging 5 ml of NP solution with 30 mL of a mixture of diethyl ether and acetone (15:15). After this initial centrifugation, **Ru@PIL(I)** NPs were centrifuged with acetone (15 mL), diethyl ether (10 mL) and pentane (10 mL) two times. The crude **Ru@PIL(I)** was next dissolved in MeOH (5 ml) and centrifuged with diethyl ether (15 mL) and pentane (15 mL). All centrifugations were carried out at 8000 rpm for 15 min at 5 °C. The resulting shiny black product was dried at 35 °C under vacuum for 24 h. This step ensures the removing of the different solvents, including ethylene glycol (b.p. = 197°C). The metal content of **Ru@PIL(I)** NPs calculated by using the TGA analysis,^[5]^ and it was found to be 4.2 wt%.


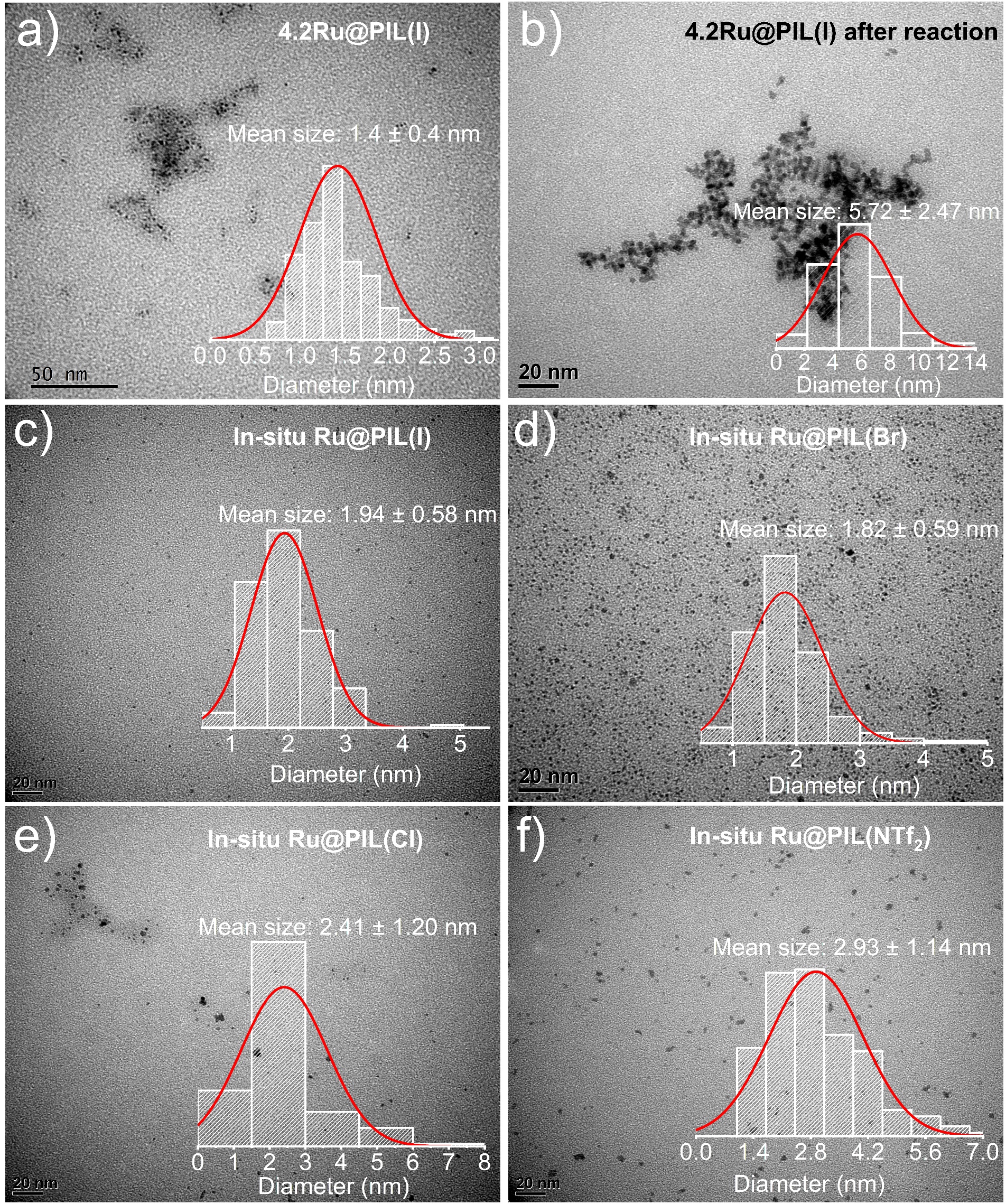


**Figure S1** TEM images and size distribution of the *ex-situ* and *in-situ* synthesized **Ru@PIL(X)** NPs (X = I, Cl, Br and NTf_2_), and the *ex-situ* prepared **Ru@PIL(I)** after reaction.


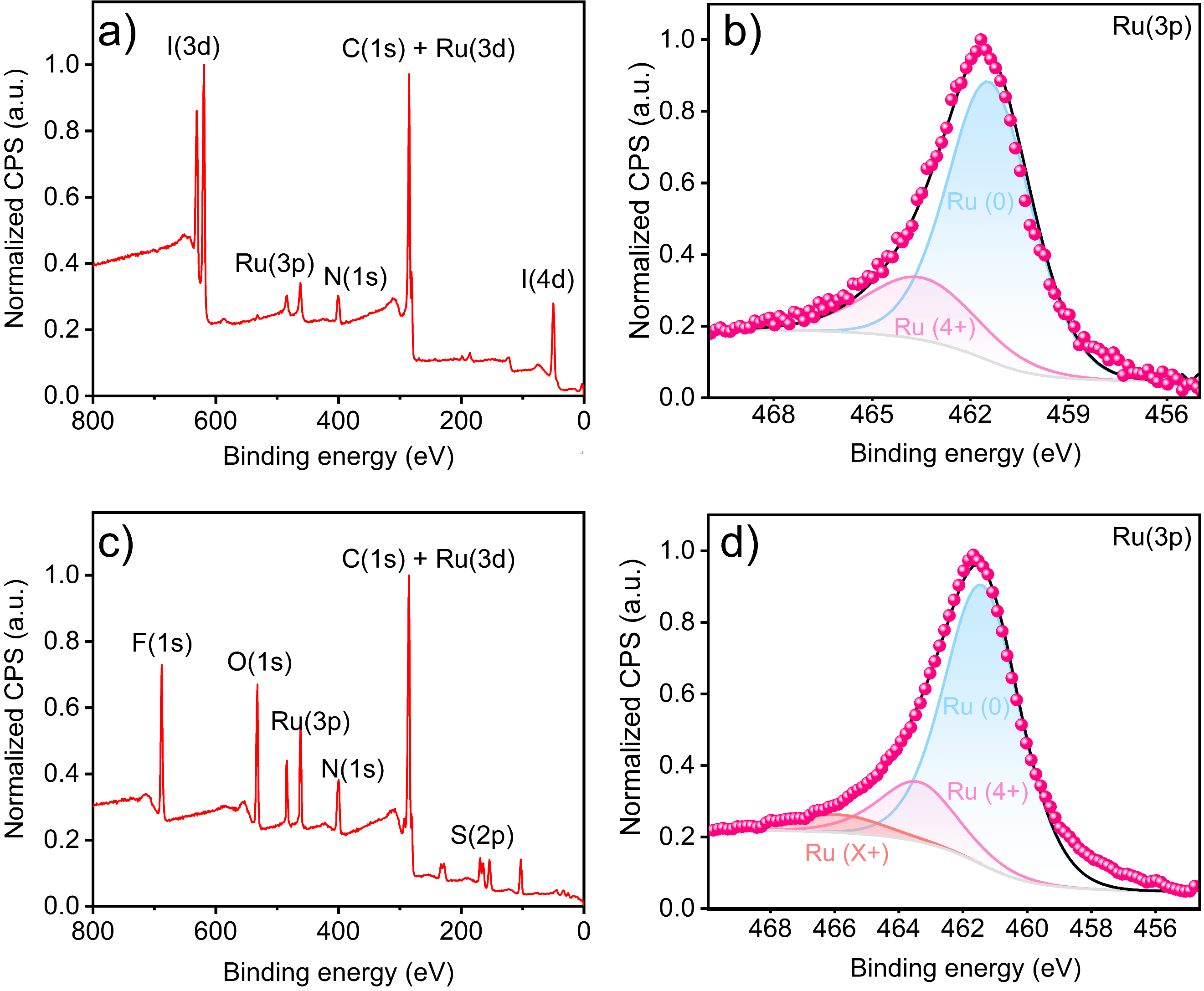


**Figure S2** XPS analysis, full scan spectrum of ex-situ synthesized **Ru@PIL(I)** (a) and its narrow scans of the Ru3p spectra (b).

**Table S2**. Different oxidation states of Ru and their proportion in NPs determined by deconvolution of Ru(3p) scan.

| **Entry** | **Sample** | **Position of peaks and Ru species** | | |
| --- | --- | --- | --- | --- |
|  |  | **Ru(0) [Area %]** | **Ru(4+) [Area %]** | **Ru(X+) [Area %]** |
| 1 | 4.2Ru@PIL(I) | 461.5 [81] | 463.5 [19] | - |
| 2 | In-situ generated Ru@ PIL(I) | 461.5 [80] | 463.7 [20] | - |
| 3 | In-situ generated Ru@PIL(NTf_2_) | 461.5 [83] | 463.7 [17] | - |

**Ru content determination by TGA** ^[5]^: Ru content of **Ru@PIL(I)** was determined by thermogravimetric analysis (TGA) using a TGA Q-500 from TA instruments. A typical TGA curve of **PIL(I)** (a) and **Ru@PIL(I)** (b) are shown in Figure S3. TGA was performed in two steps: in the first step, the samples were heated until 600 °C under nitrogen to remove **PIL**. In the next step, air was introduced after 600 °C to facilitate the removal of residual carbon by combustion. The residual weight obtained at around 615 °C was considered as the Ru content as all carbon-based matter had been removed.


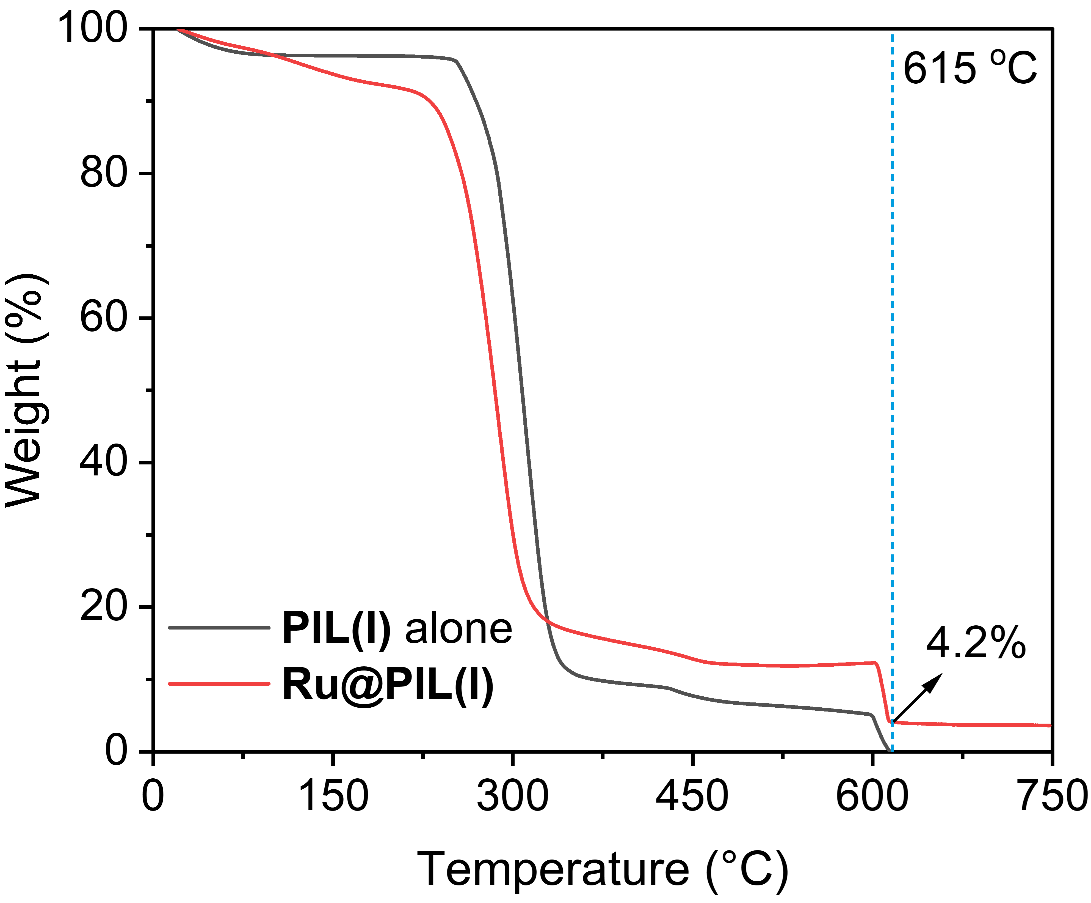


**Figure S3** TGA curve of **PIL(I)** (black line) and as-synthesized **Ru@PIL(I)** (red line), showing weight loss with temperature. Residual weight after 615 °C was considered as the Ru content of **Ru@PIL(I)**.

**General procedure for the Hydrogenation of ethylene carbonate by Ru -based catalysts** (Batch reactor)

Typical procedure for ethylene carbonate (EC) hydrogenolysis: EC (264.2 mg, 3 mmol), Ru-precursor (11.0 mg, 0.03 mmol, 0.01 equiv.), PILs (1.05 mmol, 0.35 equiv.) and a stir bar were added to a screw-thread clear vial (2 mL) and sealed in glove box. After that, the vial was put in an oil bath at 45 ^o^C overnight to make the mixture homogeneous. The Teflon cap was pierced with a needle, the vial was capped and inserted into an autoclave (5.2 mL). The autoclave was purged 3 times with H_2_ and then pressurized to the appropriate H_2_ pressure. The pressurized autoclave was placed in a heating block at the desired temperature and stirred (800 rpm) for 24 h. After the reaction, the steel autoclave was cooled in an ice-bath for 30 min. The gaseous products were carefully transferred into a collecting cell for the FT-IR analysis, then the left gas was vented off slowly. Mesitylene (58 uL, 0.42 mmol) and acetone-d_6_ (1.5 mL) was added to the suspension, and the reaction mixture was filtered using a syringe filter, The filtration was analyzed by GC-MS. The conversion of carbonates and the yields of alcohols were determined by integration of the ^1^H NMR signals with respect to the internal standard (mesitylene). Throughout the study, different batches of catalyst were prepared, characterized, and evaluated as catalysts, and were found to exhibit reproducible catalytic activity and structural characteristics (e.g. TEM).


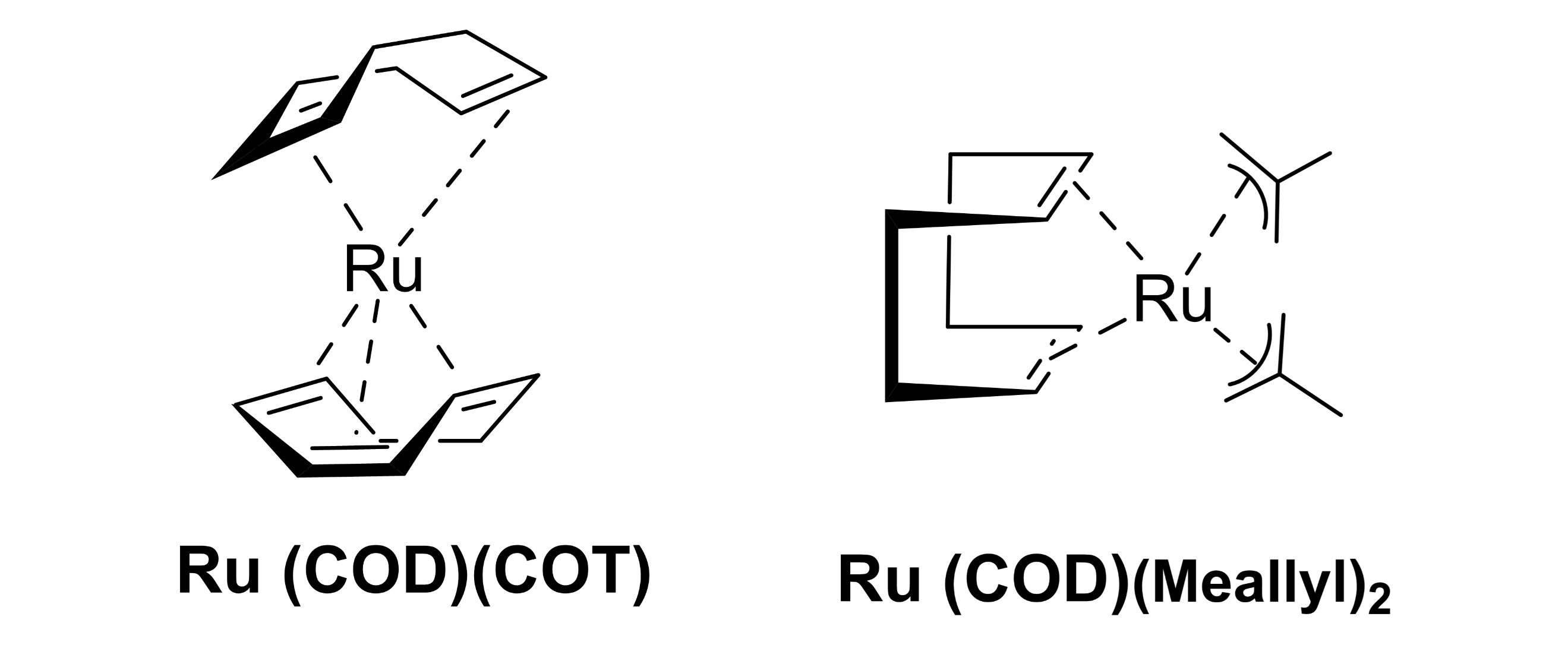


**Screening of the reaction conditions hydrogenolysis of EC under solvent-free conditions**

Optimization of the reaction conditions was initially performed with 1 mol% of Ru precursor and 3.5 mol% of PIL(NTf_2_) in neat EC (Table S3 below). Increasing the temperature from 100°C to 140°C drastically increased the EC conversion from 3% to 54%, under 40 bars of H_2_ (entries 1 and 2). By increasing the H_2_ pressure up to 70 bars, the conversion reached 80% (entry 3). Although a further increase of the temperature to 160°C (under 70 bars of H_2_) led to quantitative EC conversion, the selectivity for EG diminished (entry 4). Gratifyingly, 98% EC conversion with 96% yield of EG could be obtained at 140°C, under 40 bars of H_2_, using 2 mol% of catalyst (entry 5). In addition, to compare with a common stabilizer, the commercial PVP was adopted in the hydrogenation of EC (entry 6). Under the standard conditions, 45% conversion and 62% of selectivity for CH_4_ were obtained. Note that the liquid phase was composed of 43% of EG and 1.6% of EtOH, proving a lower activity and selectivity of Ru@PVP NPs. Furthermore, in the case of commercially available Ru/C, low EC conversion (17%) was obtained (entry 7). Thus, the experimental conditions (140°C, 24 h, under 40 bars of H_2_, using 2 mol% of Ru) were kept for the whole study.

| Entry | Stabilizer | Ru  (mol%) | T  (^o^C) | P  (bar) | Conv.  (%)^[b]^ | Liquid products (%)^[b]^ | | | Gas products (%)^[c]^ | |
| --- | --- | --- | --- | --- | --- | --- | --- | --- | --- | --- |
|  |  |  |  |  |  | EtOH | EG | MeOH | CH_4_ | CO_2_ |
| 1^[d]^ | PILs(NTf_2_) | 1 | 100 | 40 | 3 | 0 | 3 | 0 | 95 | 5 |
| 2^[d]^ | PILs(NTf_2_) | 1 | 140 | 40 | 54 | 5 | 46 | 3 | 95 | 5 |
| 3^[d]^ | PILs(NTf_2_) | 1 | 140 | 70 | 80 | 1 | 77 | 2 | 78 | 22 |
| 4^[d]^ | PILs(NTf_2_) | 1 | 160 | 70 | >99 | 5 | 88 | 6.6 | 74 | 26 |
| **5^[d]^** | PILs(NTf_2_) | **2** | **140** | **40** | **98** | **1** | **96** | **1** | **70** | **30** |
| 6^[d]^ | PVP | 2 | 140 | 40 | 45 | 1.6 | 43 | 0.4 | 62 | 38 |
| 7 | Ru/C | 2 | 140 | 40 | 16.5 | 0.9 | 15 | 0.6 | 61 | 39 |

**Table S3** Screening of the reaction conditions of the hydrogenolysis of EC under solvent-free conditions.^[a]^

[a] Reaction conditions: 3 mmol EC, 24 h. [b] The conversion of EC and the yield of alcohols are calculated based on ^1^H NMR spectrum, mesitylene as the external standard. [c] The selectivity of each gas is determined by FT-IR spectra. [d] In-situ prepared catalysts, here, Ru(0) (COD)(COT) complex is the Ru precursor, mol stabilizer / mol Ru= 3.5:1.

**^1^H NMR spectrum of the crude reaction mixture**

After 24 h reaction, the ^1^H NMR spectra of the crude reaction mixture (Figure S4) confirmed the formation of the ethanol owing to the appearance of a typical triplet at 1.21 ppm and a quartet at 3.68 ppm. Meanwhile, the high conversion of the reactant EC was highlighted by a negligible signal at 4.50 ppm.


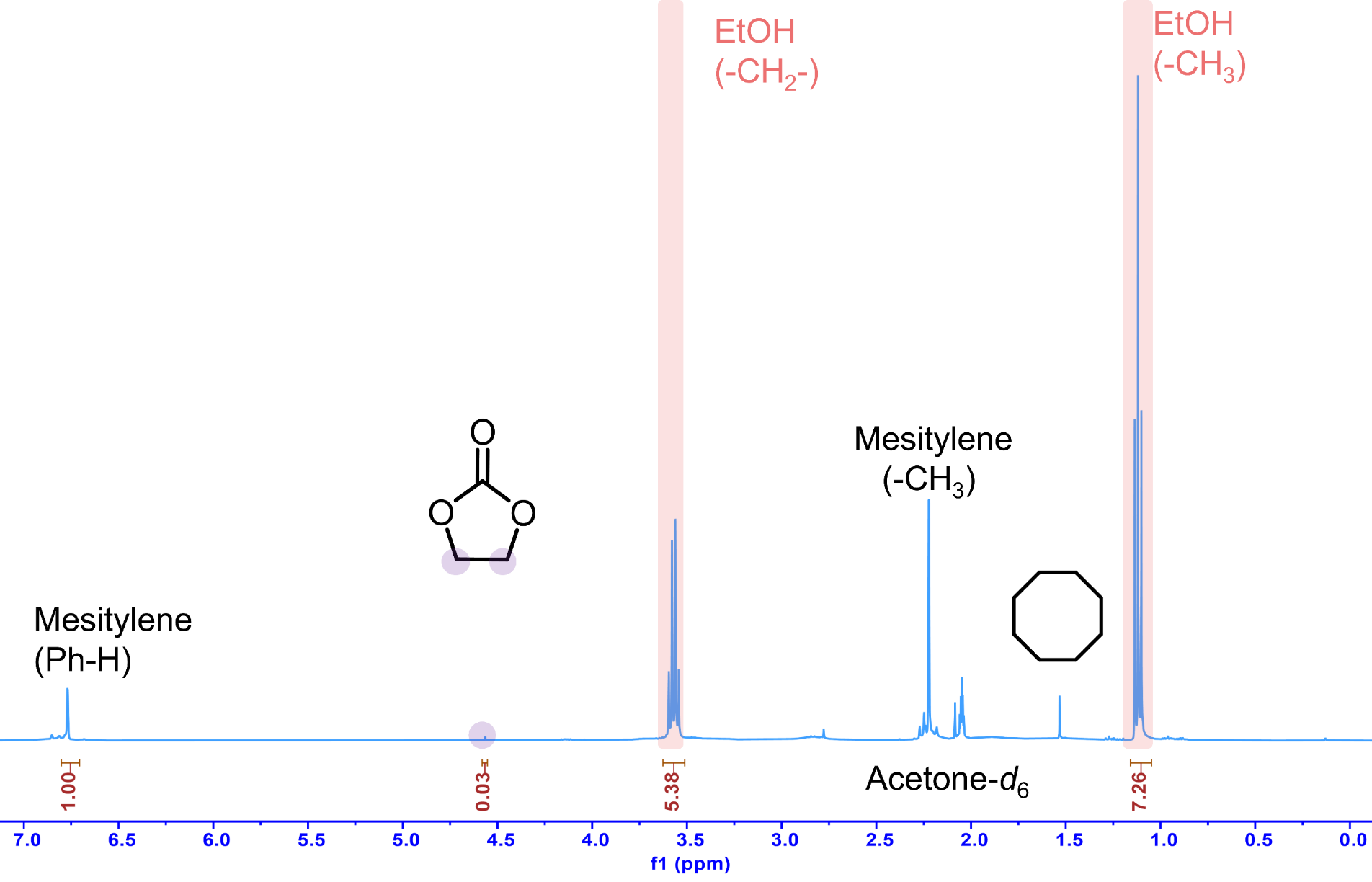


**Figure S4** ^1^H NMR (400 MHz, Acetone-d_6_, 25 ^o^C) spectrum of the hydrogenation of EC by **PIL(I)** (3.5 mol%) and Ru(II) (COD)(Meallyl)_2_ (1 mol%) (Table 1, entry 2) using mesitylene as an internal standard.

**^1^H NMR spectra: data processing**

Then, the conversion of EC and the yield of ethanol were calculated to be 99.7% and 95% respectively by comparing the relative intensities of the integration of characteristic peaks of each sample (**Eq**. S1-S3, SI).


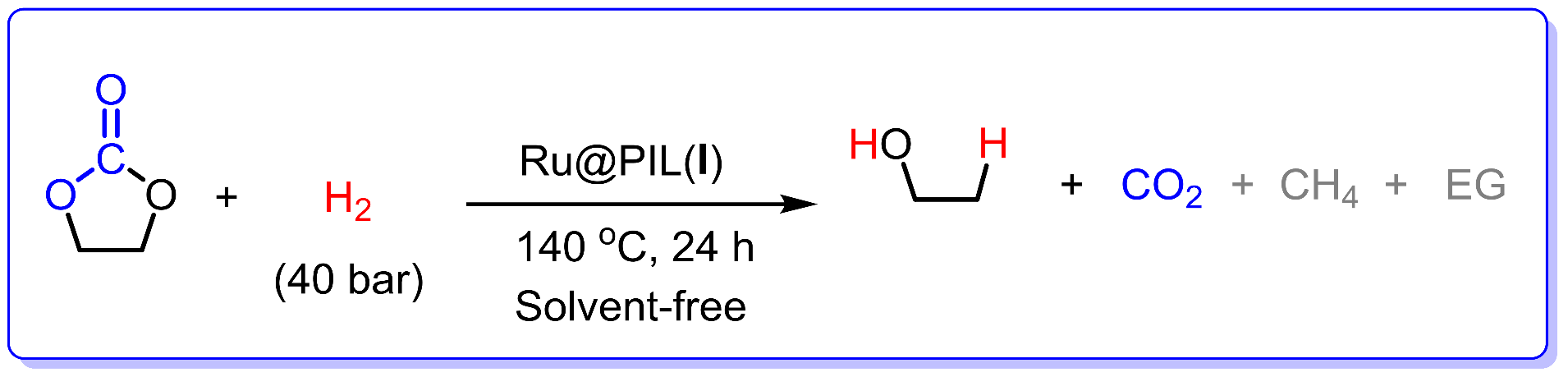

$$\frac{A_{{EC}_{(-C_{2}H_{4})}}}{N_{{EC}_{(-C_{2}H_{4})}}\times n_{{EC}_{left}}}=\frac{A_{{Mesitylene}_{(Ph-H)}}}{N_{{Mesitylene}_{(Ph-H)}}\times n_{Mesitylene}} (\boldsymbol{Eq}.S1-1)$$

$Conversion\% \left( EC \right)=100\%\times(1-\frac{n_{{EC}_{left}}}{n_{{EC}_{initial}}}$) $(\boldsymbol{Eq}.S1-2)$

$$\frac{A_{{EtOH}_{({-CH}_{3})}}}{N_{{EtOH}_{({-CH}_{3})}}\times n_{EtOH}}=\frac{A_{{Mesitylene}_{(Ph-H)}}}{N_{{Mesitylene}_{(Ph-H)}}\times n_{Mesitylene}} (\boldsymbol{Eq}.S2-1)$$

$Yield\% (EtOH)=100\%\times(1-\frac{n_{EtOH}}{n_{{EC}_{initial}}}$)$(\boldsymbol{Eq}.S2-2)$

$$\frac{A_{{EG}_{(-C_{2}H_{4})}}}{N_{{EG}_{(-C_{2}H_{4})}}\times n_{EG}}=\frac{A_{{EtOH}_{({-CH}_{2})}}-{\frac{2}{3}A}_{{EtOH}_{({-CH}_{3})}}}{N_{{EG}_{(-C_{2}H_{4})}}\times n_{EG}} =====================\frac{A_{{Mesitylene}_{(Ph-H)}}}{N_{{Mesitylene}_{(Ph-H)}}\times n_{Mesitylene}} (\boldsymbol{Eq}.S3-1)$$

$Yield\% (EG)=100\%\times(1-\frac{n_{EG}}{n_{{EC}_{initial}}}$)$(\boldsymbol{Eq}.S3-2)$

Here, A stands for an integration area; N represents the number of protons; n means the molar of a compound. The signal associated with the reference protons (3) of mesitylene are integrated as 1.

**GC-MS spectrum of the crude reaction mixture**

In order to confirm qualitatively the presence of ethanol as the main product, we have performed GC-MS (Gas chromatography-mass spectrometry) analysis of the crude reaction mixture (Figure S5). We clearly observe an intense peak at 5.51 min that correspond to EtOH together with the peak of cyclooctane and mesitylene at 6.71 and 9.93 min respectively. We could also detect at 13.48 min a weak peak corresponding to the signal of EG that is obtained as a secondary product.


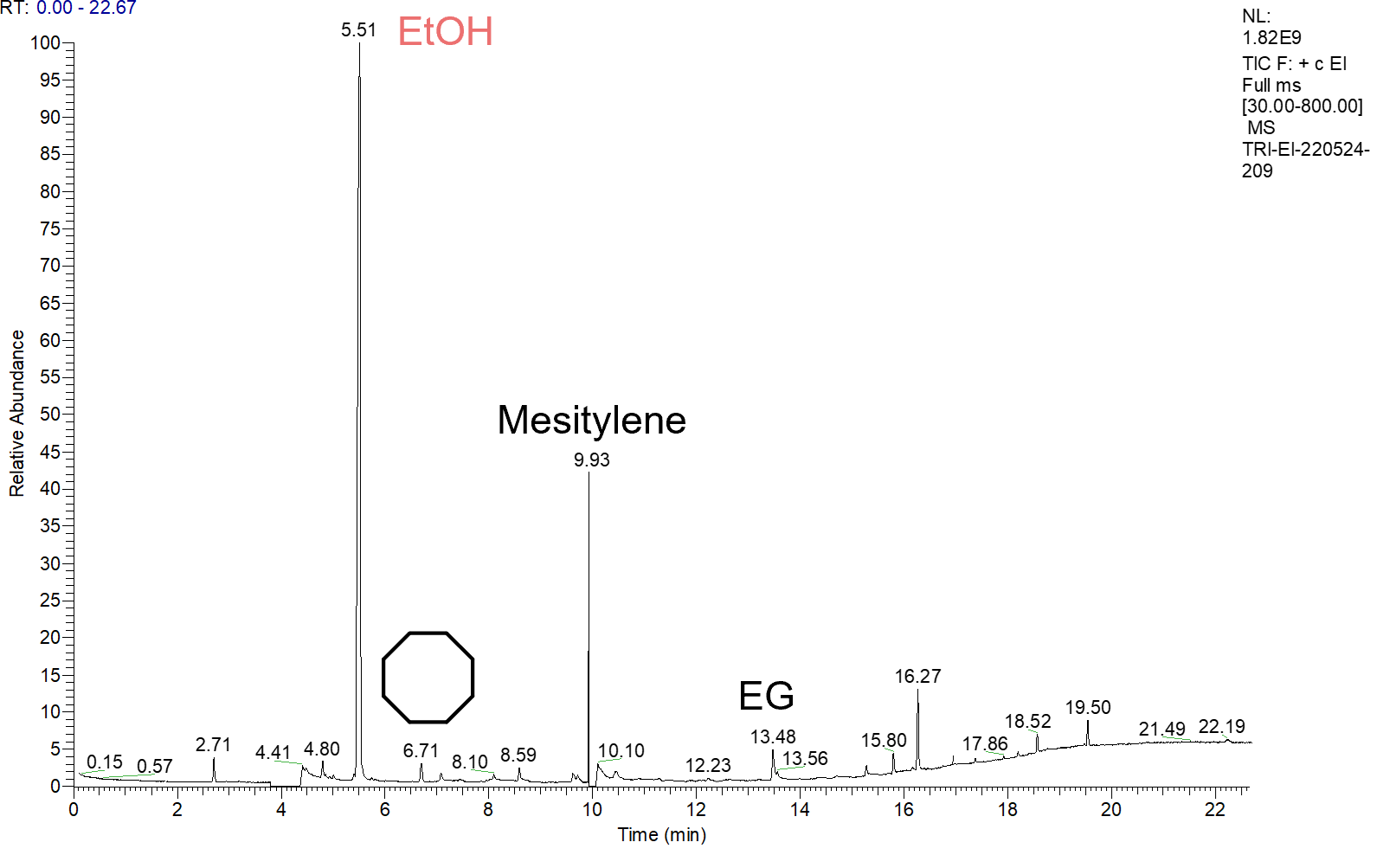


**Figure S5** GC-MS spectrum of the hydrogenation of EC by **PIL(I)** (3.5 mol%) and Ru(II) (COD)(Meallyl)_2_ (1 mol%) (Table 1, entry 2) using mesitylene as an internal standard.

**
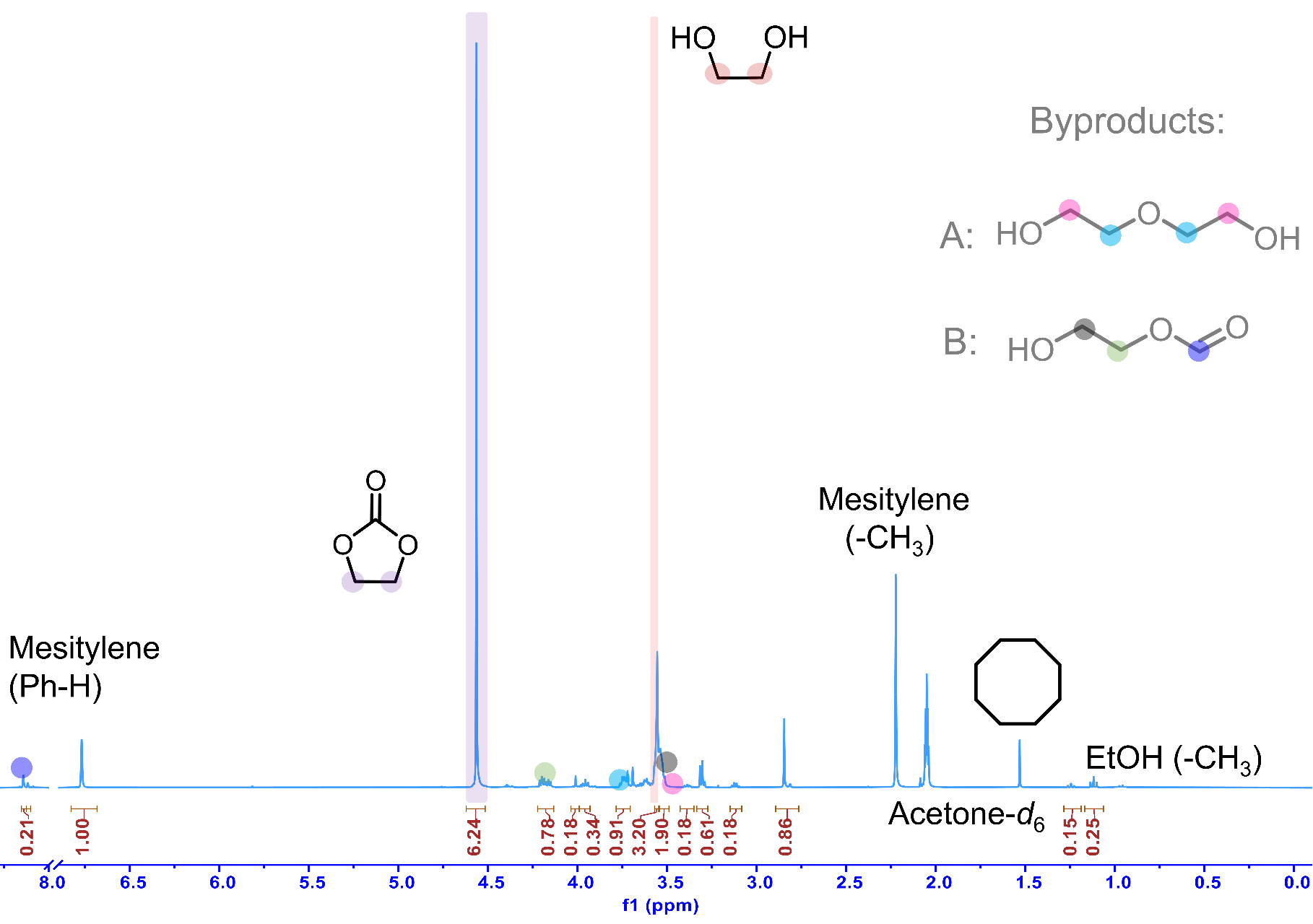
**

**Figure S6** ^1^H NMR (400 MHz, Acetone-d_6_, 25 ^o^C) spectrum of the hydrogenation of EC by **PIL(Br)** (3.5 mol%) and Ru(II) (COD)(Meallyl)_2_ (1 mol%) (Table 1, entry 3) using mesitylene as an internal standard. The side-product A was generated from the dehydration of EG, and byproduct B was formed by the hydrogenation of EC with one equivalent H_2_.


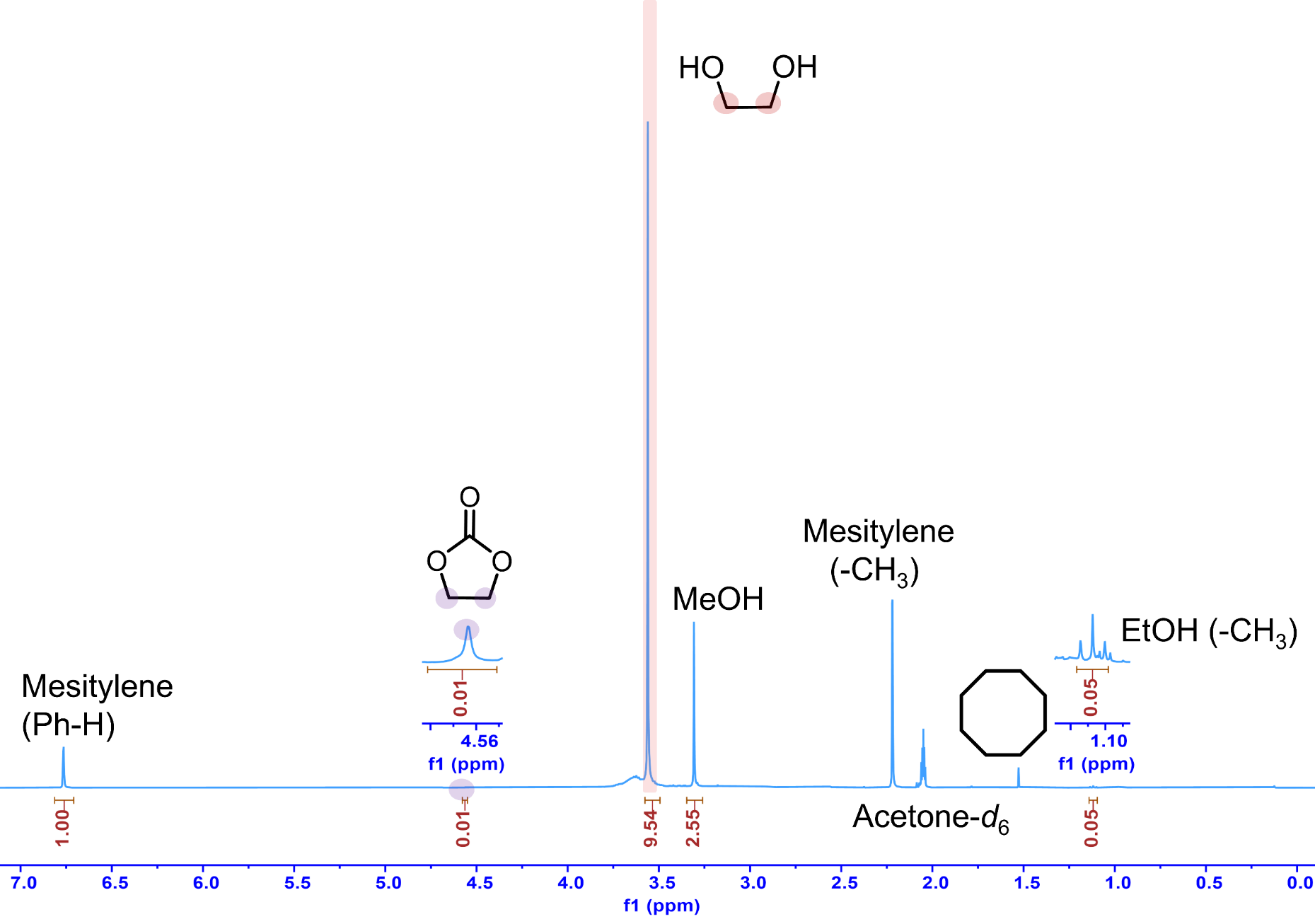


**Figure S7** ^1^H NMR (400 MHz, Acetone-d_6_, 25 ^o^C) spectrum of the hydrogenation of EC by **PIL(Cl)** (3.5 mol%) and Ru(II) (COD)(Meallyl)_2_ (1 mol%) (Table 1, entry 4) using mesitylene as an internal standard.

**
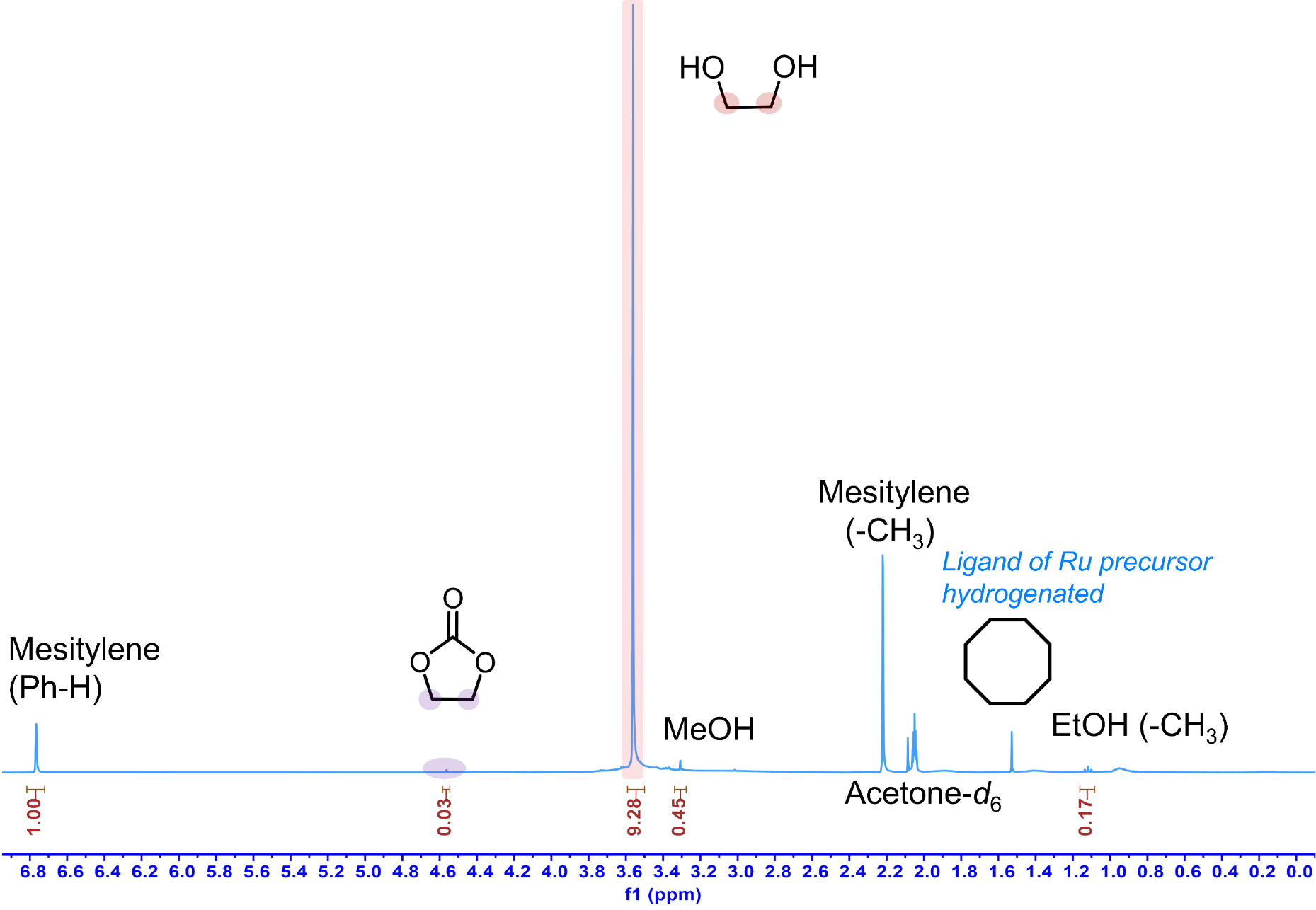
**

**Figure S8** ^1^H NMR (400 MHz, Acetone-d_6_, 25 ^o^C) spectrum of the hydrogenation of EC by **PIL(NTf_2_)** (7 mol%) and Ru(II) (COD)(Meallyl)_2_ (2 mol%) (Table 1, entry 5) using mesitylene as an internal standard.

**^1^H NMR spectra: data processing**

Then, the conversion of EC and the yield of EG were calculated to be 99.7% and 95% respectively by comparing the relative intensities of the integration of characteristic peaks of each sample (**Eq**. S1-S3, S4-S5, SI).


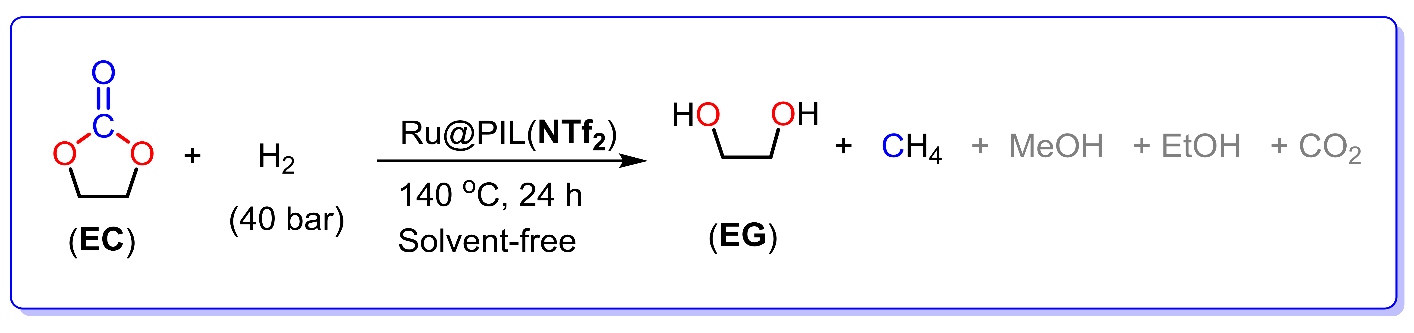

$$\frac{A_{{EC}_{(-C_{2}H_{4})}}}{N_{{EC}_{(-C_{2}H_{4})}}\times n_{{EC}_{left}}}=\frac{A_{{Mesitylene}_{(Ph-H)}}}{N_{{Mesitylene}_{(Ph-H)}}\times n_{Mesitylene}} (\boldsymbol{Eq}.S1-1)$$

$Conversion\% \left( EC \right)=100\%\times(1-\frac{n_{{EC}_{left}}}{n_{{EC}_{initial}}}$) $(\boldsymbol{Eq}.S1-2)$

$$\frac{A_{{EG}_{(-C_{2}H_{4})}}}{N_{{EG}_{(-C_{2}H_{4})}}\times n_{EG}}=\frac{A_{{Mesitylene}_{(Ph-H)}}}{N_{{Mesitylene}_{(Ph-H)}}\times n_{Mesitylene}} (\boldsymbol{Eq}.S4-1)$$

$Yield\% (EG)=100\%\times(1-\frac{n_{EG}}{n_{{EC}_{initial}}}$)$(\boldsymbol{Eq}.S4-2)$

$$\frac{A_{{MeOH}_{({-CH}_{3})}}}{N_{{MeOH}_{({-CH}_{3})}}\times n_{MeOH}}=\frac{A_{{Mesitylene}_{(Ph-H)}}}{N_{{Mesitylene}_{(Ph-H)}}\times n_{Mesitylene}} (\boldsymbol{Eq}.S5-1)$$

$Yield\% (MeOH)=100\%\times(1-\frac{n_{MeOH}}{n_{{EC}_{initial}}}$)$(\boldsymbol{Eq}.S5-2)$

$$\frac{A_{{EtOH}_{({-CH}_{3})}}}{N_{{EtOH}_{({-CH}_{3})}}\times n_{EtOH}}=\frac{A_{{Mesitylene}_{(Ph-H)}}}{N_{{Mesitylene}_{(Ph-H)}}\times n_{Mesitylene}} (\boldsymbol{Eq}.S2-1)$$

$Yield\% (EtOH)=100\%\times(1-\frac{n_{EtOH}}{n_{{EC}_{initial}}}$)$(\boldsymbol{Eq}.S2-2)$

Here, A stands for an integration area; N represents the number of protons; n means the molar of a compound. The signal associated with the reference protons (3) of mesitylene are integrated as 1.

**GC-MS spectrum of the crude reaction mixture** (Table 1, entries 3-5)


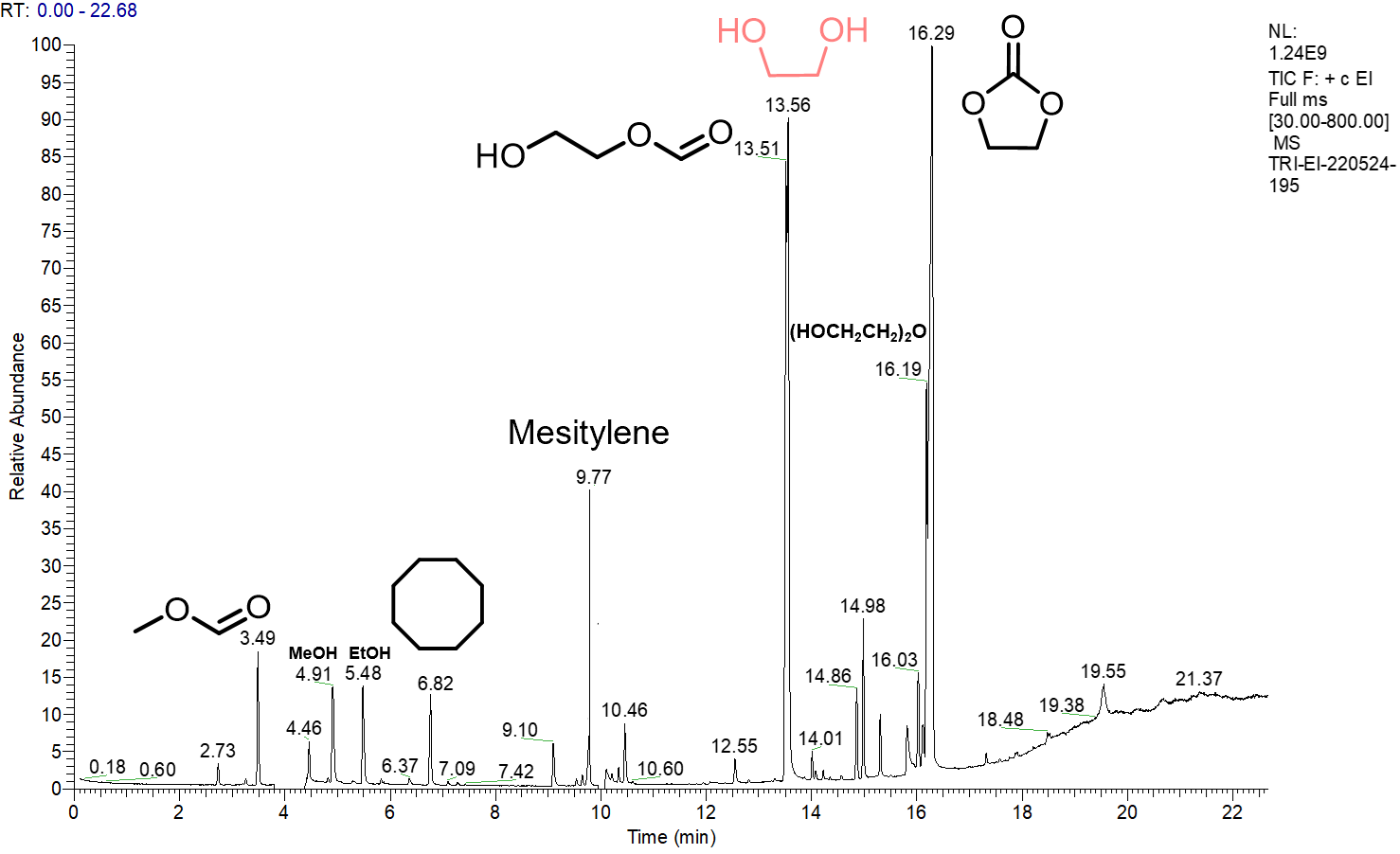


**Figure S9** GC-MS spectrum of the hydrogenation of EC by **PIL(Br)** (3.5 mol%) and Ru(II) (COD)(Meallyl)_2_ (1 mol%) (Table 1, entry 7) using mesitylene as an internal standard. The peak at 13.51 belongs to HOCH_2_CH_2_OCHO formed by hydrogenation of EC with one equivalent H_2_. And diethylene glycol was found at 16.19, generated from the dehydration of EG.

**
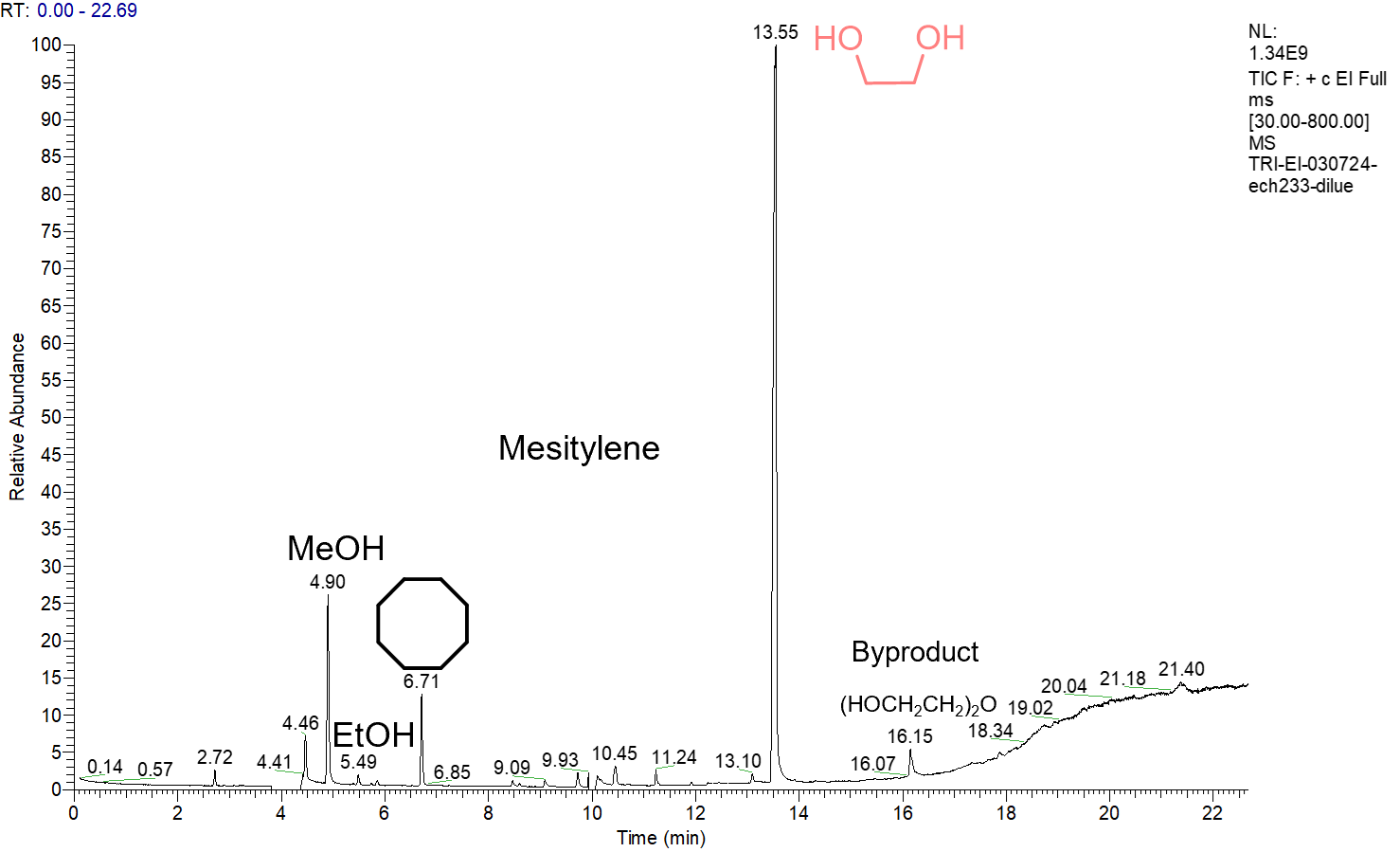
**

**Figure S10** GC-MS spectrum of the hydrogenation of EC by **PIL(Cl)** (3.5 mol%) and Ru(II) (COD)(Meallyl)_2_ (1 mol%) (Table 1, entry 6) using mesitylene as an internal standard.

**
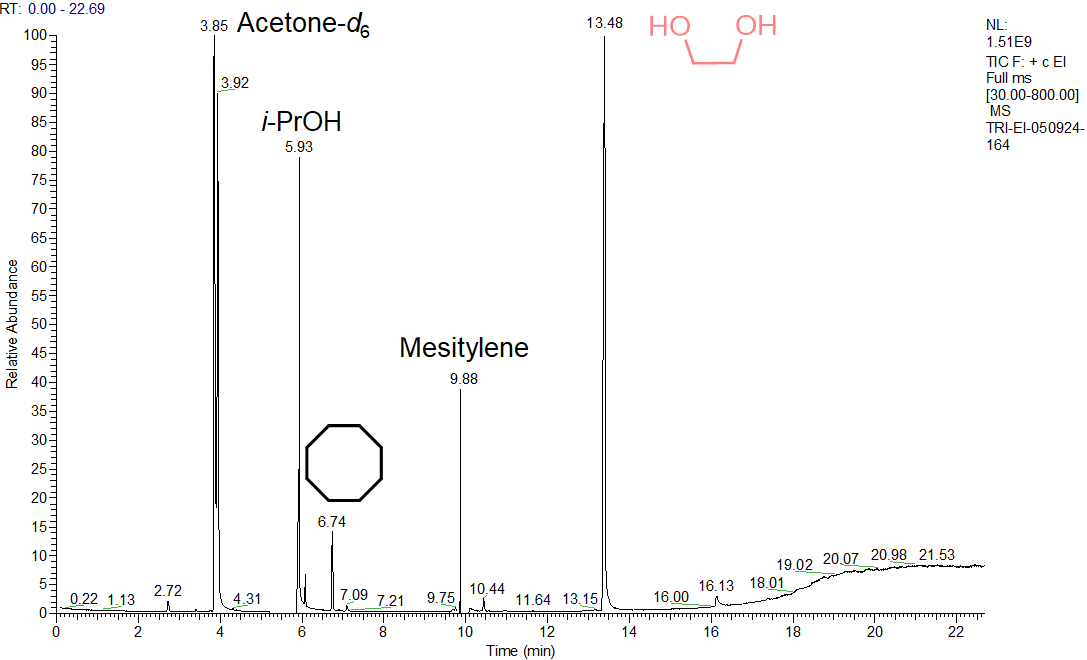
**

**Figure S11** GC-MS spectrum of the hydrogenation of EC by **PIL(NTf_2_)** (7 mol%) and Ru(II) (COD)(Meallyl)_2_ (2 mol%) (Table 1, entry 5) using mesitylene as an internal standard. In the case of Ru@**PIL(NTf_2_)**, *i*-PrOH is used to dilute the reaction mixture to remove the catalyst for GC-MS analysis unless otherwise mentioned.**FTIR spectrum of the gas phase of the crude reaction mixture**

The FTIR spectra of the gas phase of the crude reaction mixture obtained after 24 h reaction is shown in Figures S12-15. The formation of CO_2_ and CH_4_ were demonstrated by the FTIR spectra. For CO_2_, the vibrational peaks from 2550 to 2150 cm^-1^ (asymmetric stretching contribution 𝜈_3_), and the peaks from 3800 to 3500 cm^-1^ (combination modes 2𝜈_2_+ 𝜈_3_ in left doublet and the modes 𝜈_1_+ 𝜈_3_ in right doublet, where 𝜈_1_ is the symmetric stretch) were found.^8^ Meanwhile, there are the asymmetric bending mode 𝜈_4_ (1390 to 1216 cm^-1^) and asymmetric stretching 𝜈_3_ (3200 to 2800 cm^-1^) of CH_4_ in Figure S12.^9^ In addition, the FTIR spectrum of the crude sample also highlights the presence of a secondary product that has been assigned to the C=O stretching mode (1805 to 1690 cm^-1^) of ethylformate(EtOCHO), but its amount (below 1 %) is negligible. (Figures S12, 13-14).

Then, the selectivity towards the formation of CO_2_ and CH_4_ were determined from the intensities of the characteristic peaks of each sample (Eq. S6-S4, SI).

^
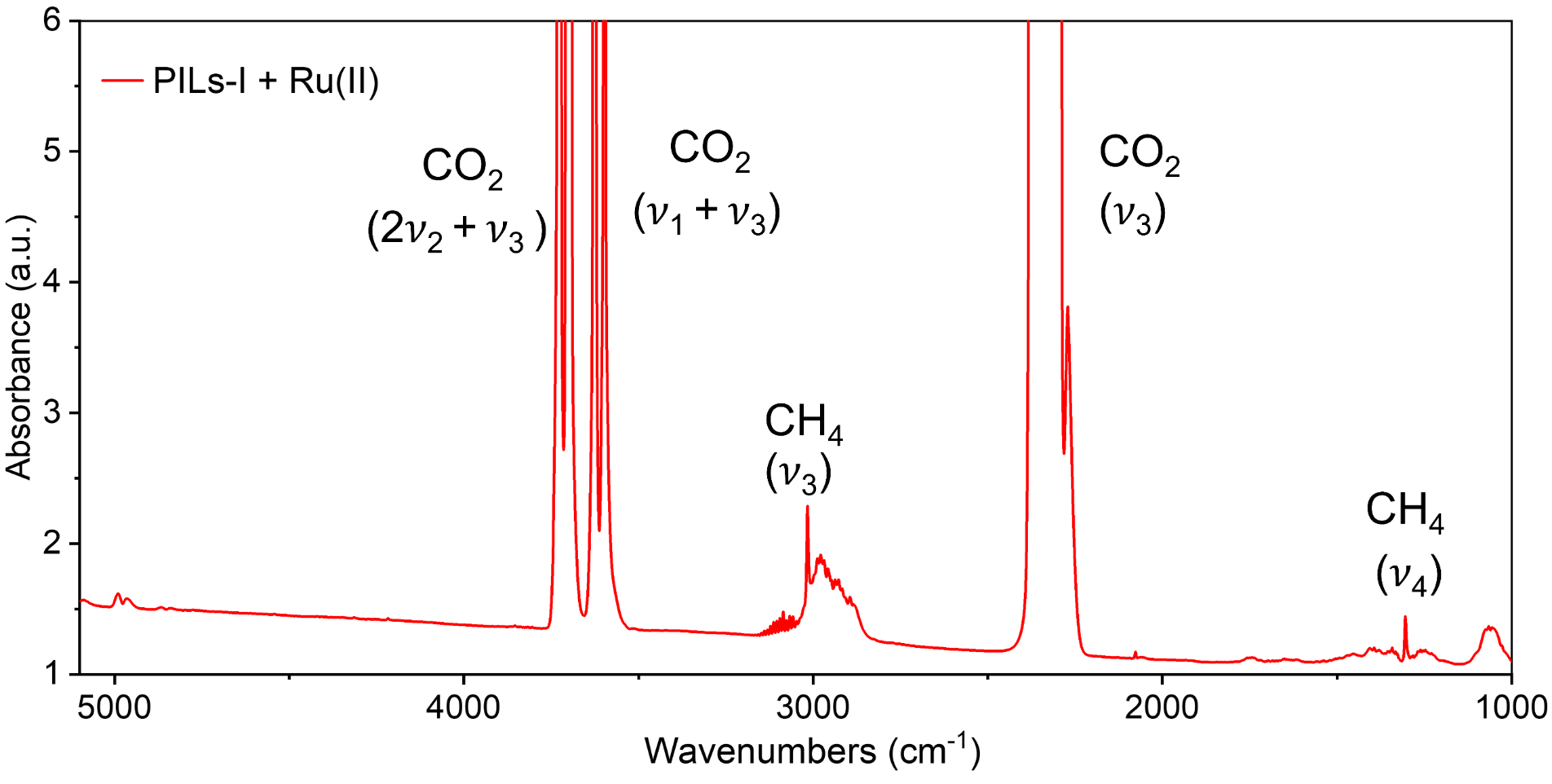
^

**Figure S12** FT-IR spectrum of the gas phase of the hydrogenation of EC by **PIL(I)** and Ru(II) (COD)(Meallyl)_2_ (Table 1, entry 2).


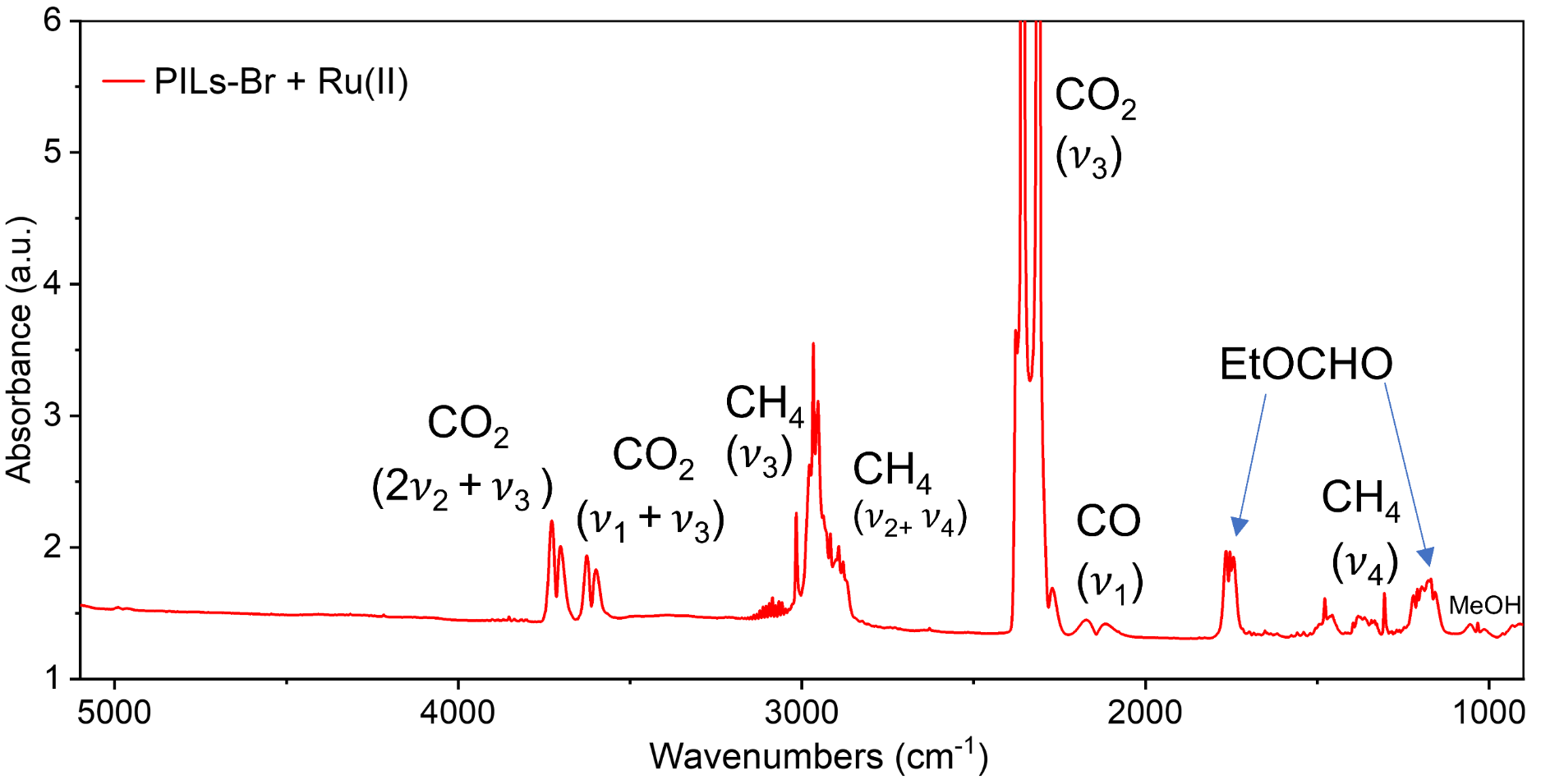


**Figure S13** FT-IR spectrum of the gas phase of the hydrogenation of EC by **PIL(Br)** and Ru(II) (COD)(Meallyl)_2_ (Table 1, entry 3).


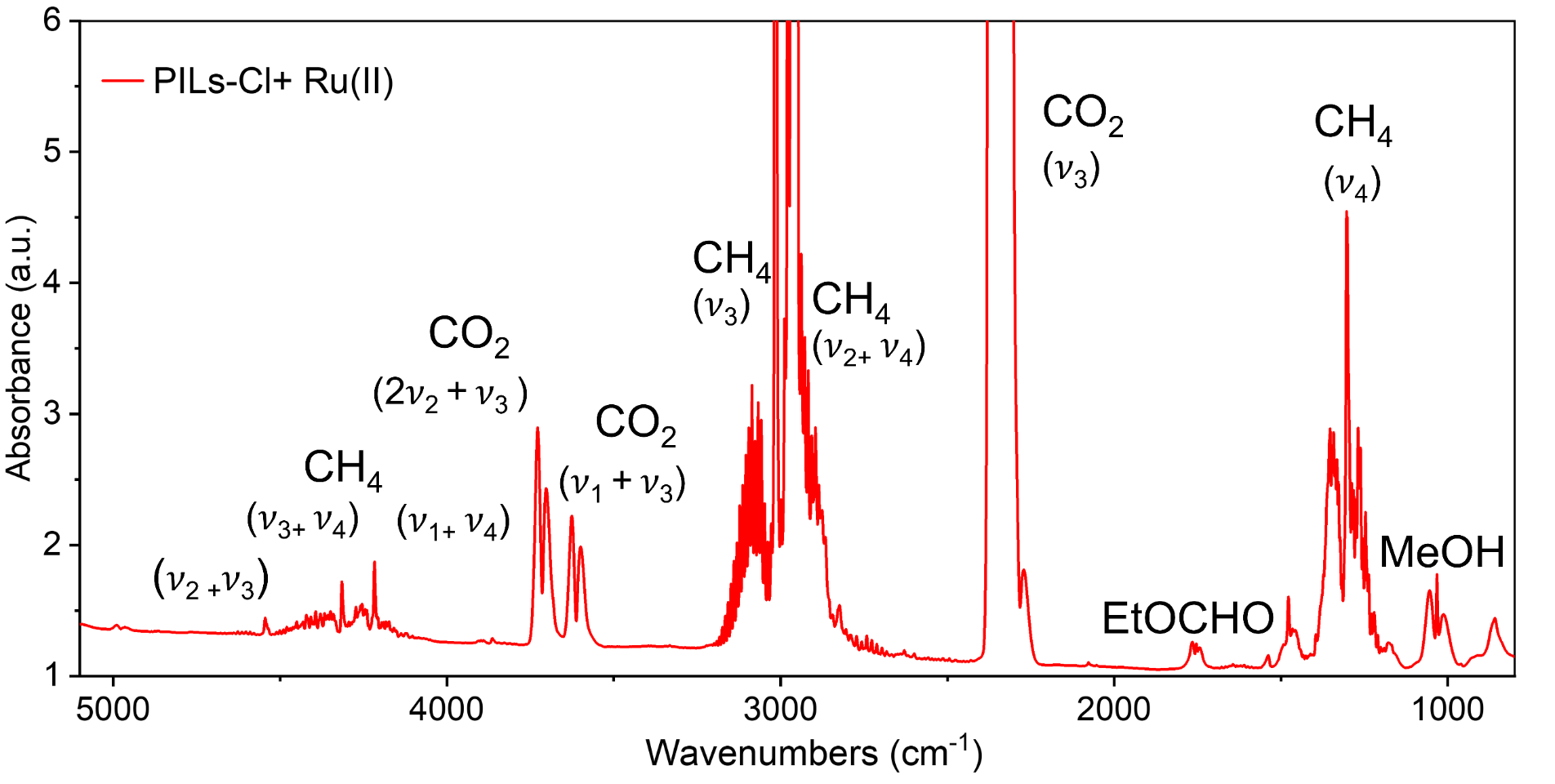


**Figure S14** FT-IR spectrum of the gas phase of the hydrogenation of EC by **PIL(Cl)** and Ru(II) (COD)(Meallyl)_2_ (Table 1, entry 4).


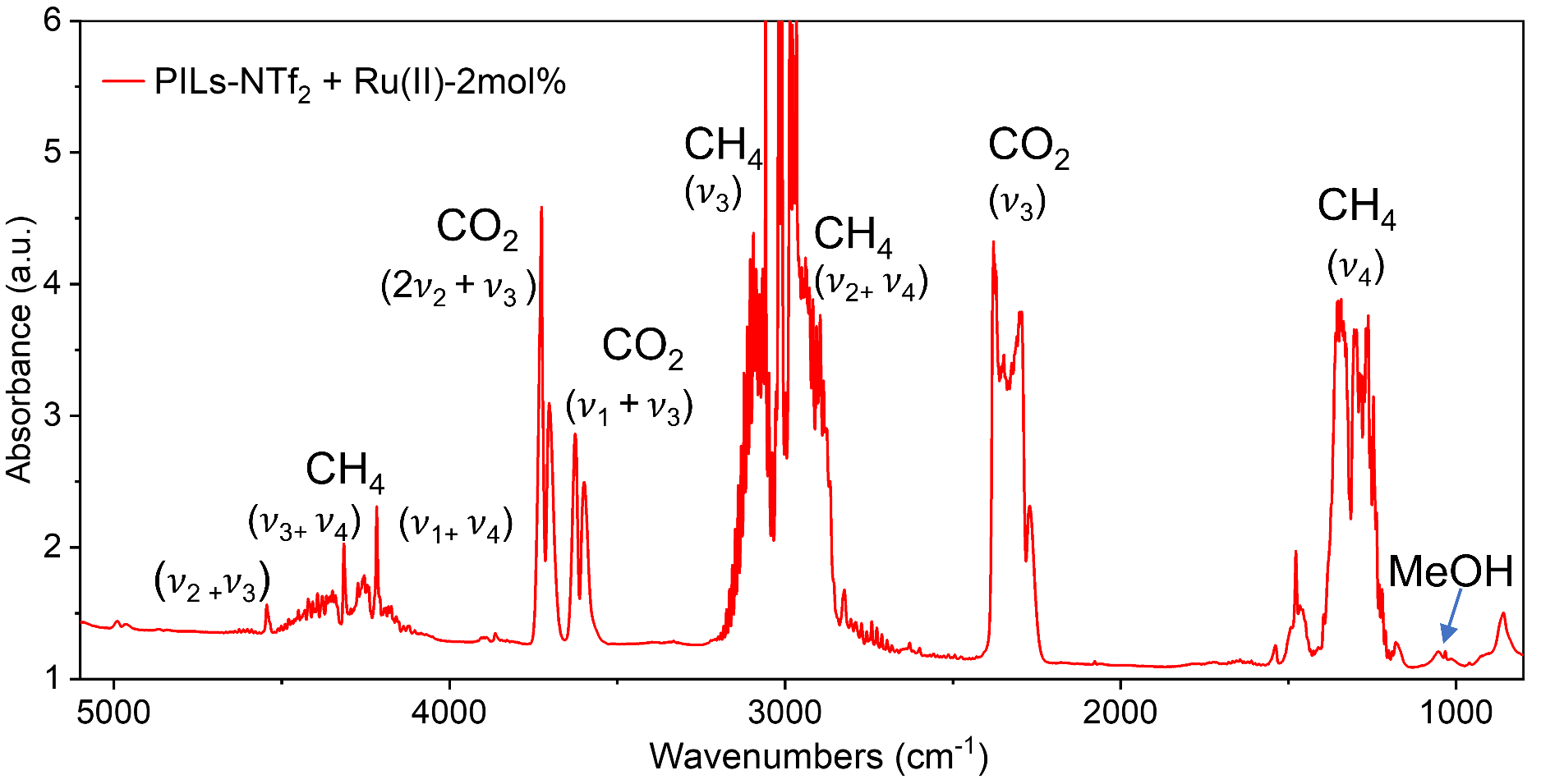


**Figure S15** FT-IR spectrum of the gas phase of the hydrogenation of EC by **PIL(NTf_2_)** and Ru(II) (COD)(Meallyl)_2_ (Table 1, entry 5). The multiple peaks of CH_4_ from 4727 to 3953 cm^-1^ contains the combination modes 𝜈_2_+ 𝜈_3_, 𝜈_3_+ 𝜈_4_ and 𝜈_1_+ 𝜈_4_ (where 𝜈_1_ is the symmetric stretch, 𝜈_2_ is the symmetric bend) were found.^[10]^

**Infrared absorption spectra: data processing**

To calculate the concentration of each gas, the Beer Lambert law was applied. First, the concentration of each gas was calculated based on ***Eq***. S6:

$$C_{\mathrm{CO}_{2}}=\frac{H_{{CO}_{2}}}{\varepsilon_{{CO}_{2}}\times l} (\boldsymbol{Eq}.S6-1)$$

$$C_{\mathrm{CH}_{4}}=\frac{A_{{CH}_{4}}}{\varepsilon_{{CH}_{4}}\times l} (\boldsymbol{Eq}.S6-2)$$

$$C_{CO}=\frac{A_{CO}}{\varepsilon_{CO}\times l} (\boldsymbol{Eq}.S6-3)$$

$$C_{EtOCHO}=\frac{A_{EtOCHO}}{\varepsilon_{EtCHO}\times l} (\boldsymbol{Eq}.S6-4)$$

Where H is the peak height at 2270.806 cm^-1^ of the selected peak of CO_2_, the base line is from 2423 to 2222 cm^-1^. A is the peak area of the selected peak (CH_4_ or CO), the base line is from 1393 to 1226 cm^-1^ or 4727 to 3953 cm^-1^ for CH_4_ and is from 2241 to 2000 cm^-1^ for CO; ε is the molar extinction coefficient of the peak (L•mol^−1^•cm^−1^) and l the path length (2.5 cm). $\varepsilon_{{CO}_{2}}$, $\varepsilon_{{CH}_{4}}(1393 to 1226 \mathrm{cm}^{-1})$, $\varepsilon_{{CH}_{4}}$ $(4727 to 3953 \mathrm{cm}^{-1})$ and $\varepsilon_{CO}$ are determined to be 6.1519, 645.8540, 190 and 1857.25 L•mol^−1^•cm^−1^ respectively after calibration.^[^[^9^](#_ENREF_2)^]^ Then, the selectivity of each gas was calculated by using ***Eq.*** S7.

$Selectivity\% \left( {CO}_{2} \right)=\frac{C_{{CO}_{2}}}{C_{{CO}_{2}} +C_{CO} + C_{{CH}_{4}} + C_{EtOCHO}}\times100\%$ $(\boldsymbol{Eq}.S7-1)$

$Selectivity\% \left( {CH}_{4} \right)=\frac{C_{CH_{4}}}{C_{{CO}_{2}} +C_{CO} + C_{{CH}_{4}} + C_{EtOCHO}}\times100\%$ $(\boldsymbol{Eq}.S7-2)$

$Selectivity\% \left( CO \right)=\frac{C_{CO}}{C_{{CO}_{2}} +C_{CO} + C_{{CH}_{4}} + C_{EtOCHO}}\times100\%$ $(\boldsymbol{Eq}.S7-3)$

The gas phase of the decarboxylation of EC was monitored by in-situ IR spectroscopy, using PIL(I) as catalyst. Ethylene oxide (EO), characterized by a band at 3065 cm^-1^, could be detected after 15 min at 140 °C together with the peaks of CO_2_ at 3610 and 3715 cm^-1^ (Figure S16 below).


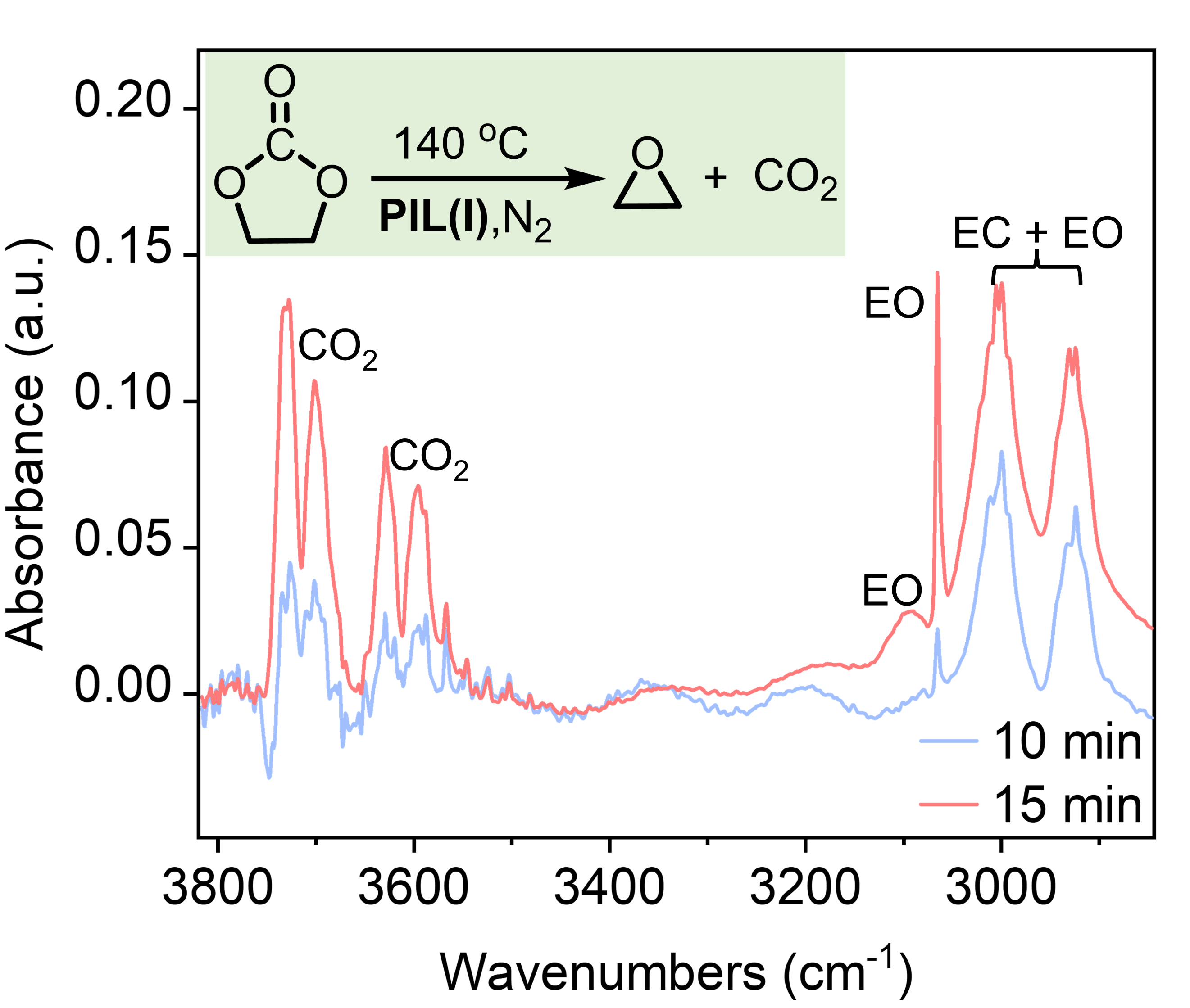


**Figure S16** In-situ FTIR spectra of neat EC at 140 °C under 40 bar of N_2_ in the presence of 3.5 mol% PIL(I) after 10 min of heating from 25 to 140 °C (Blue curve) and after 15min of reaction (Red curve).

**Table S4** Hydrogenation of propylene oxide (PO) with Ru@PIL(I)**.**

|  | | | |  |
| --- | --- | --- | --- | --- |
| Entry | Conversion (%)^[b]^ | Liquid products (%)^[c]^ | | |
|  |  | *i*PrOH | 1-PrOH | |
| 1 | 100 | 94 | 6 | |

^a^ Reaction conditions: PO (3 mmol), PILs-I (3.5 mol%), Ru(II) (COD)(Meallyl)_2_ (1 mol%), 24 h, 100 ^o^C. ^b^ The conversion of EC and the yield of alcohols are calculated based on ^1^H NMR spectrum, mesitylene as the internal standard.

To evidence the ability of Ru@PIL(I) to catalyze the hydrogenation of epoxides, propylene oxide (PO) was investigated rather than EO because it is a liquid that is more conveniently and safely handled (Table S4). After 24 h at 100 °C, full PO conversion was observed. The liquid phase was composed of a mixture of two isomeric mono-alcohol, namely i-PrOH (94%) and n-PrOH (6%) (Figures S17-S18).


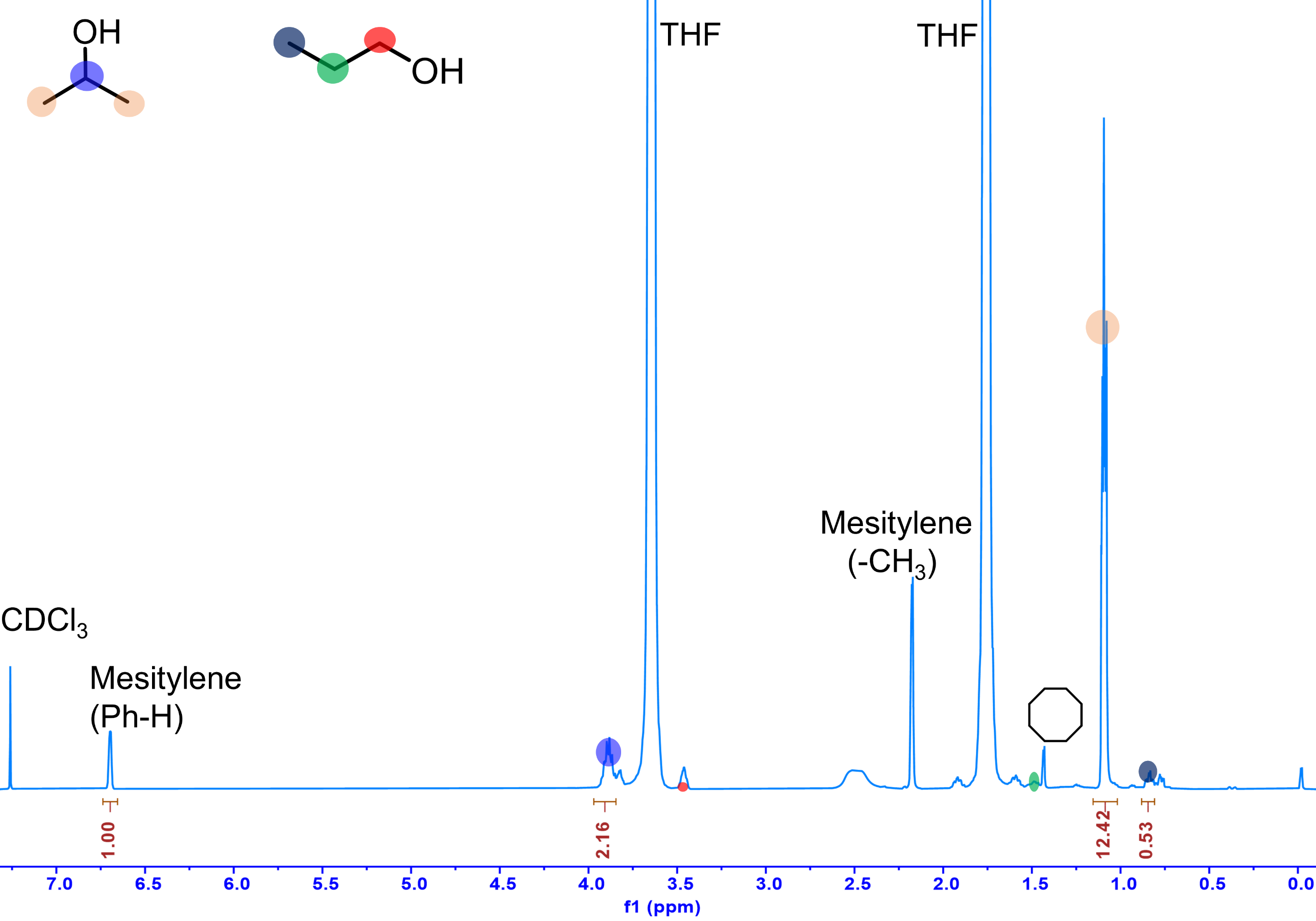


**Figure S17** ^1^H NMR spectrum of the hydrogenation of PO by **PIL(I)** (3.5 mol%) and Ru(II) (COD)(Meallyl)_2_ (1 mol%) using mesitylene as an internal standard.


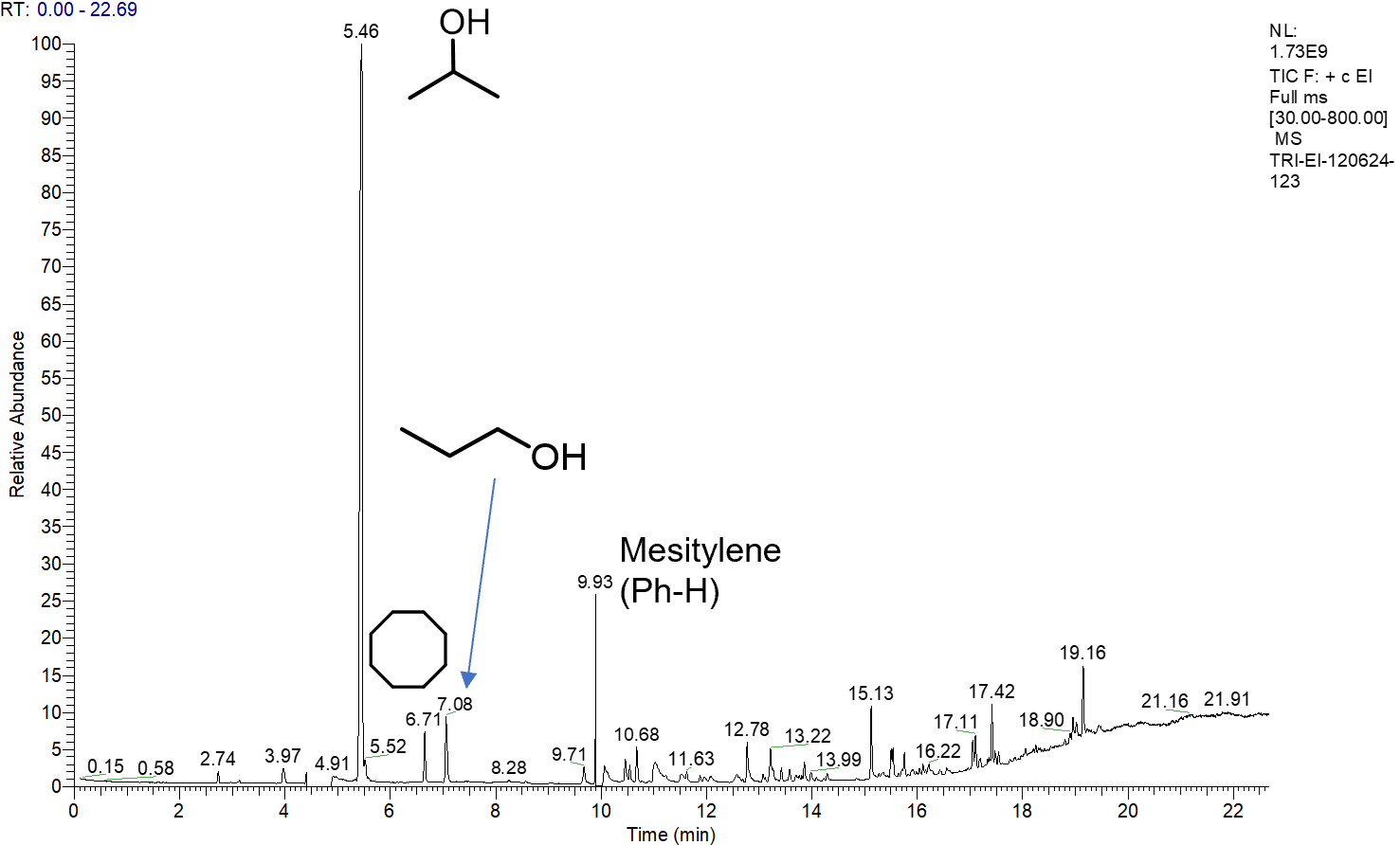


**Figure S18** GC-MS spectrum of the hydrogenation of PO by **PILs-I** (3.5 mol%) and Ru(II) (COD)(Meallyl)_2_ (1 mol%) using mesitylene as an internal standard.

**Proposed mechanisms for the “hydrogenative decarboxylation” of EC catalyzed by *in-situ* generated Ru@PIL(I).**


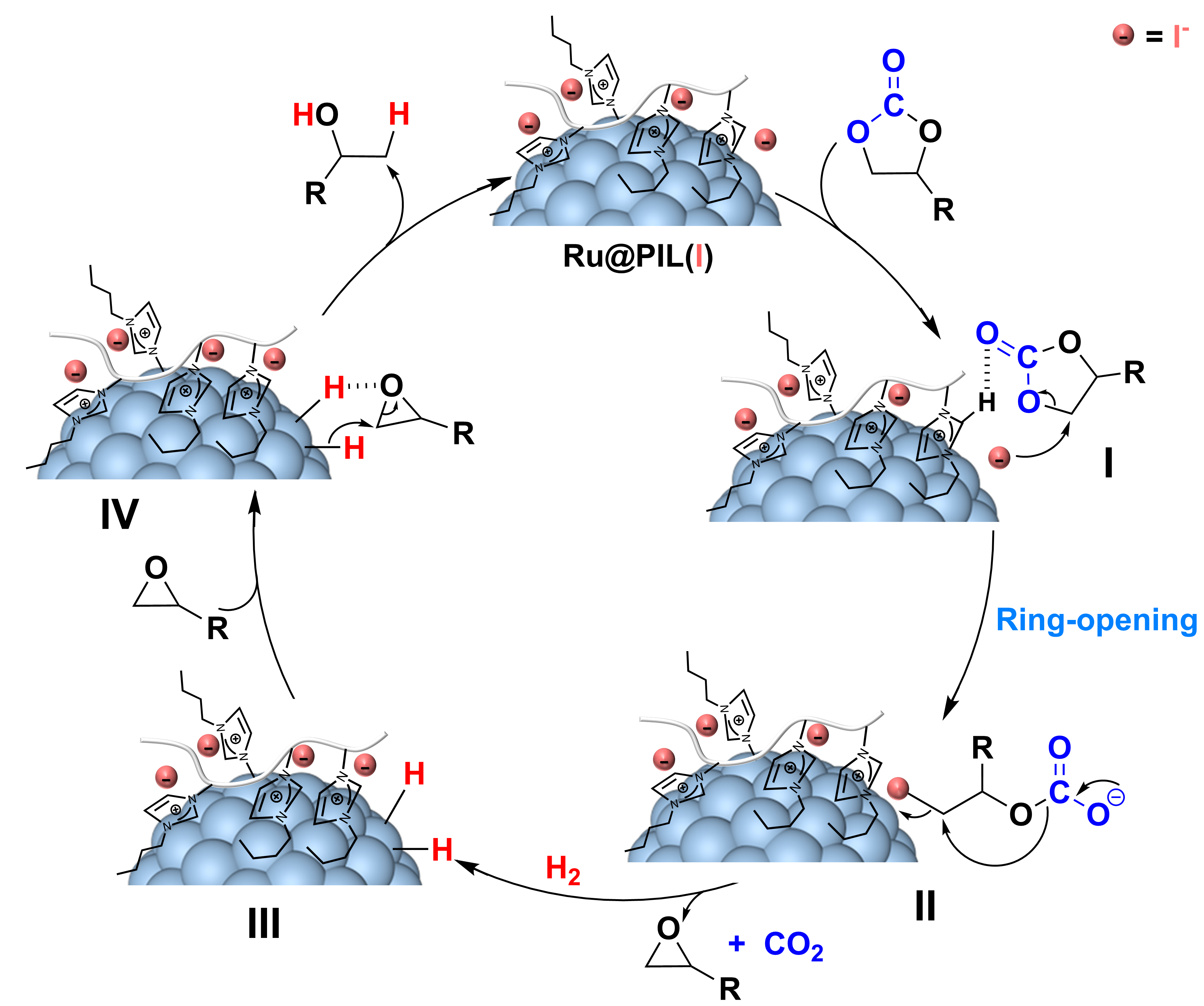


**Scheme S2.** Proposed reaction mechanism of the hydrogenation of cyclic carbonates by Ru@PIL(I).

The proposed mechanism was hypothesized according to the experimental results (Scheme S2). First, the imidazolium proton of PIL(I) can activate the carbonate by hydrogen bonding, thereby increasing its electrophilicity. Then, EC can undergo a ring opening triggered by the nucleophilic I^-^ anion of PIL(I), leading to the intermediate **II**. Decarboxylation of **II** generates an epoxide and CO_2_. Then the H_2_ was activated and disassociated on the surface of Ru NPs, for hydrogenation of the epoxide. The decarboxylation reaction of EC to generate EO and CO_2_ being thermodynamically unfavorable, the coupling of this decarboxylation step with the highly exergonic hydrogenation of EO into EtOH push the overall transformation towards the formation of EtOH.

**Proposed mechanisms for the “direct hydrogenation” of EC catalyzed by *in-situ* generated Ru@PIL(NTf_2_).**


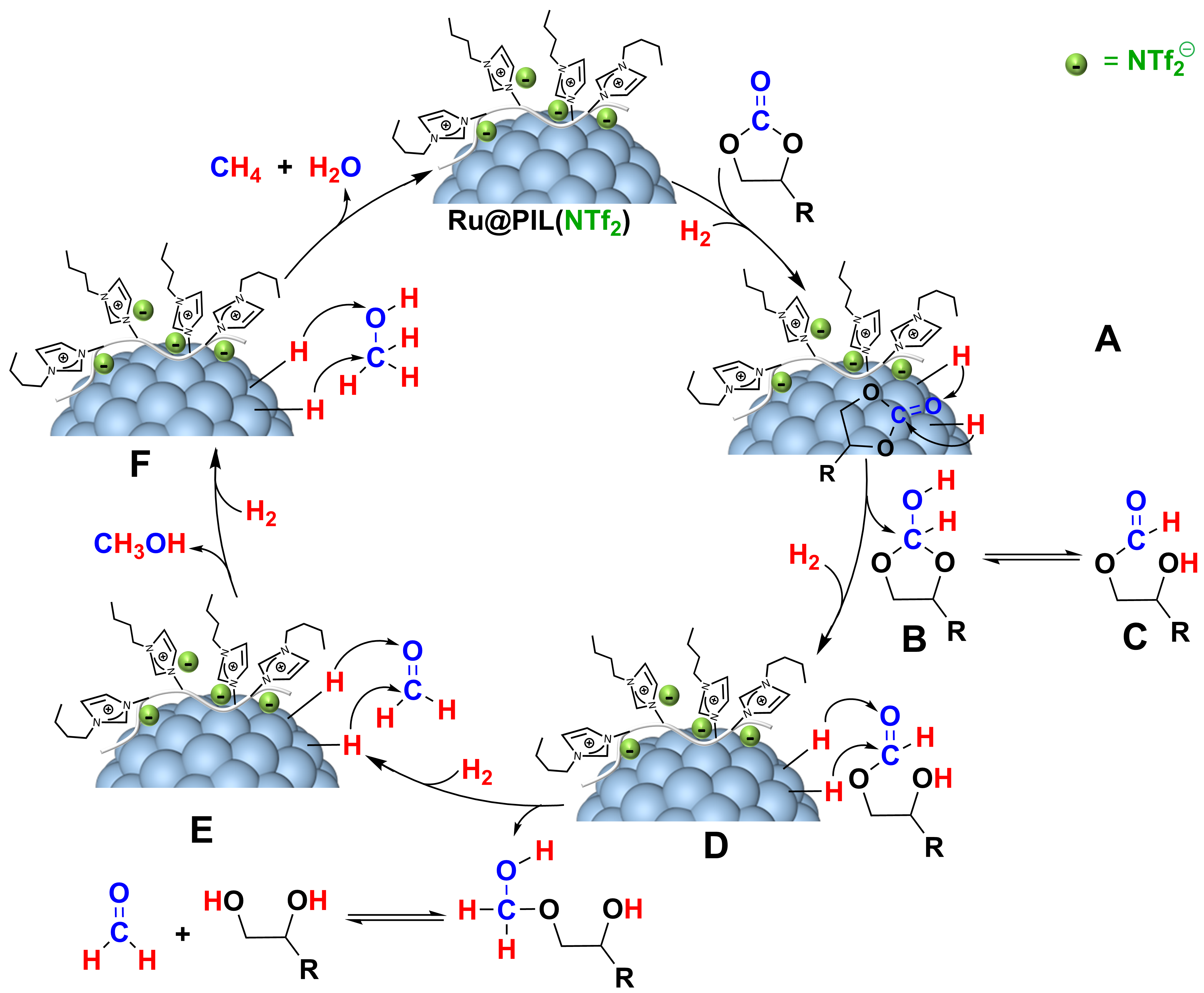


**Scheme S3** Proposed mechanisms of hydrogenation of EC by Ru@PIL(NTf_2_) generated in-situ.

Based on the experimental results and the reported literature,^[11]^ the following mechanisms is proposed (Scheme S3). In particular, it accounts for the different products detected (yet in low amount) by GC/MS analysis.

**Table S5 (All information of Table 2).** Screening the scope of different substrates by Ru@PIL(I) prepared in-situ.^[a]^

|  | | | | | | | | |
| --- | --- | --- | --- | --- | --- | --- | --- | --- |
| Entry | Substrate | Conv.  (%)^[b]^ | Liquid products (Yield) | | | Gas products | | |
|  |  |  | R’OH (%)^[b]^ | | Diols (%)^[b]^ | (Selectivity %)^[c]^ | | |
|  |  |  |  |  |  | CH_4_ | CO_2_ | CO |
| 1 | **1a** | >99 | **2a** (95) | | 5 | 5 | 95 | 0 |
| 2^[d]^ | **1b** | 88 |   **2b** (75) | 14.7 | 9.2 | 7 | 93 | 0 |
| 3^[d]^ | **1c** | 88 |   **2c** (74) | 10.4 | 0 | 5 | 95 | 0 |
| 4 | **1d** | 99 | **2d** (93) | 6.7 | 0 | 8 | 92 | 0 |
| 5 | **1e** | 61 | **2e** (58) | 9.9 | 14.5 | 16 | 84 | 0 |
| 6 | **1f** | 100 |   **2f** (28) | | 21 | 27 | 73 | 0 |

[a] Reaction conditions: 3 mmol substrates, 1 mol% Ru(II) (COD)(Meallyl)_2_, 3.5 mol% PIL(I). [b] The conversion of EC and the yield of alcohols are calculated based on ^1^H NMR spectrum, mesitylene as the internal standard. [c] The selectivity of each gas is determined by FT-IR spectra. [d] 160 ^o^C.

**Table S6 (All information of Table 3).** Hydrogenation of different substrates by in-situ generated Ru@PIL(NTf_2_). ^[a]^

|  | | | | | | | |
| --- | --- | --- | --- | --- | --- | --- | --- |
| Entry | Substrate | Conv.  (%)^[b]^ | Liquid products (Yield) | | | Gas products | |
|  |  |  | Diols (%)^[b]^ | R’OH (%)^[b]^ | | (Selectivity %)^[c]^ | |
|  |  |  |  |  |  | CH_4_ | CO_2_ |
| 1 | **1a** | 99.7 |   **3a** (95) | EtOH (2)  MeOH (5) | | 73 | 27 |
| 2^[d]^ | **1b** | 99 |   **3b** (76) | 0 | 0 | 79 | 21 |
| 3^[d]^ | **1c** | 78 |   **3c** (70) | 4.3 | 0 | 76 | 24 |
| 4 | **1d** | 100 |   **3d** (100) | 0 | 0 | 75 | 25 |
| 5 | **1e** | >99 |   **3e** (99) | 0 | 0 | 75 | 25 |
| 6 | **1f** | 98.6 |   **3f** (24) | 1-Pr-OH (6) | | 83 | 17 |
| 7 | MeOH | 89.2 | 0 | 0 | 0 | 100 | 0 |

[a] Reaction conditions: 3 mmol substrates, 2 mol% Ru(II) (COD)(Meallyl)_2_, 7 mol% PIL(NTf_2_). [b] The conversion of EC and the yield of alcohols are calculated based on ^1^H NMR spectrum, mesitylene as the external standard. [c] The selectivity of each gas is determined by FT-IR spectra. [d] 160 ^o^C.

**^1^H NMR spectrum and GC-MS of different substrates in Table 2.**

**
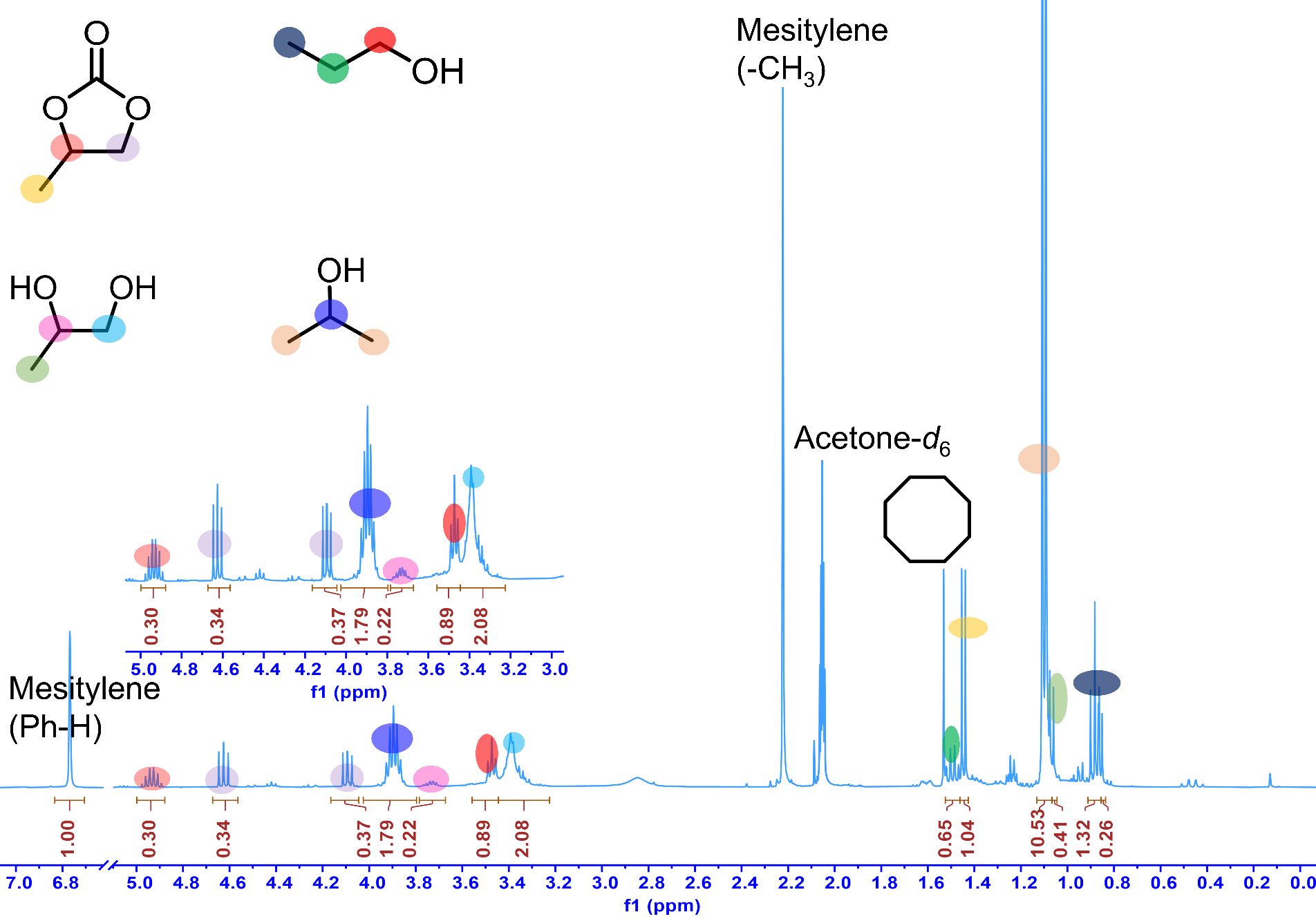
**

**Figure S19** ^1^H NMR (400 MHz, Acetone-d_6_, 25 ^o^C) spectrum of the hydrogenation of PC (**1b**) by **PIL(I)** (3.5 mol%) and Ru(II) (COD)(Meallyl)_2_ (1 mol%) (Table 2, **2b**, entry 2) using mesitylene as an internal standard.

**
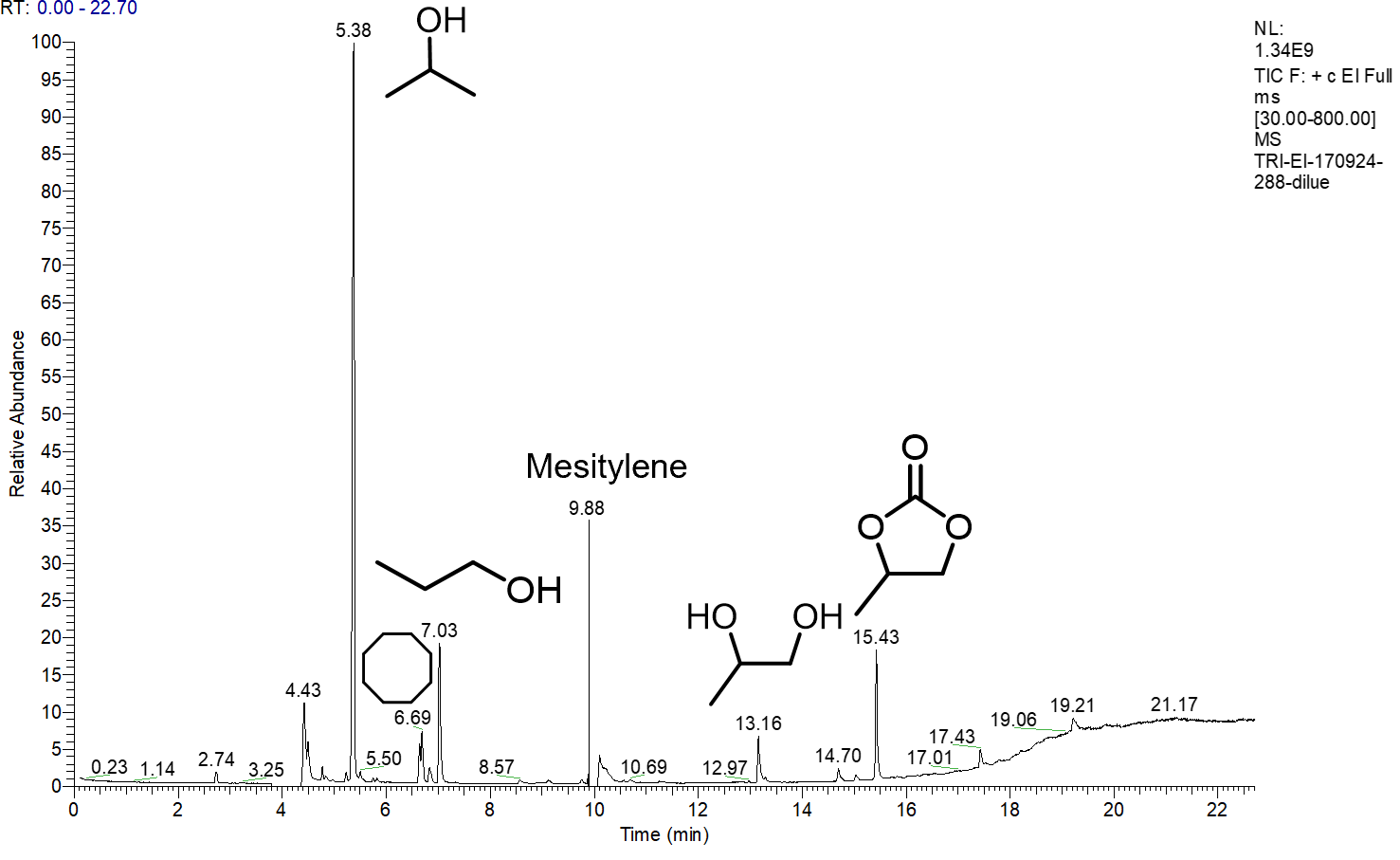
**

**Figure S20** GC-MS spectrum of the hydrogenation of **1b** by **PIL(I)** (3.5 mol%) and Ru(II) (COD)(Meallyl)_2_ (1 mol%) (Table 2, **2b**, entry 2)using mesitylene as an internal standard.

**
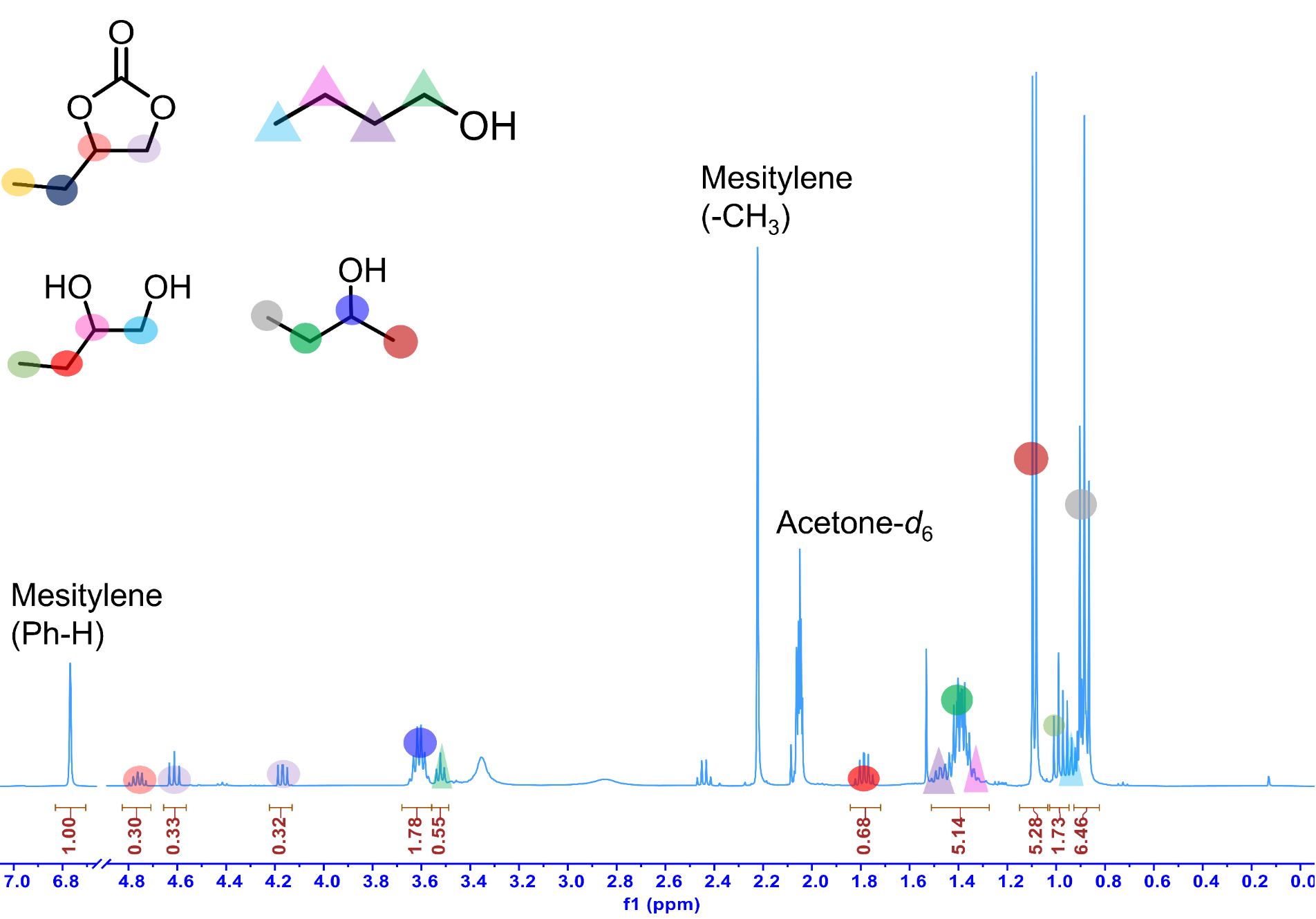
**

**Figure S21** ^1^H NMR (400 MHz, Acetone-d_6_, 25 ^o^C) spectrum of the hydrogenation of BC (**1c**) by **PIL(I)** (3.5 mol%) and Ru(II) (COD)(Meallyl)_2_ (1 mol%) (Table 2, **2c**, entry 3) using mesitylene as an internal standard.

**
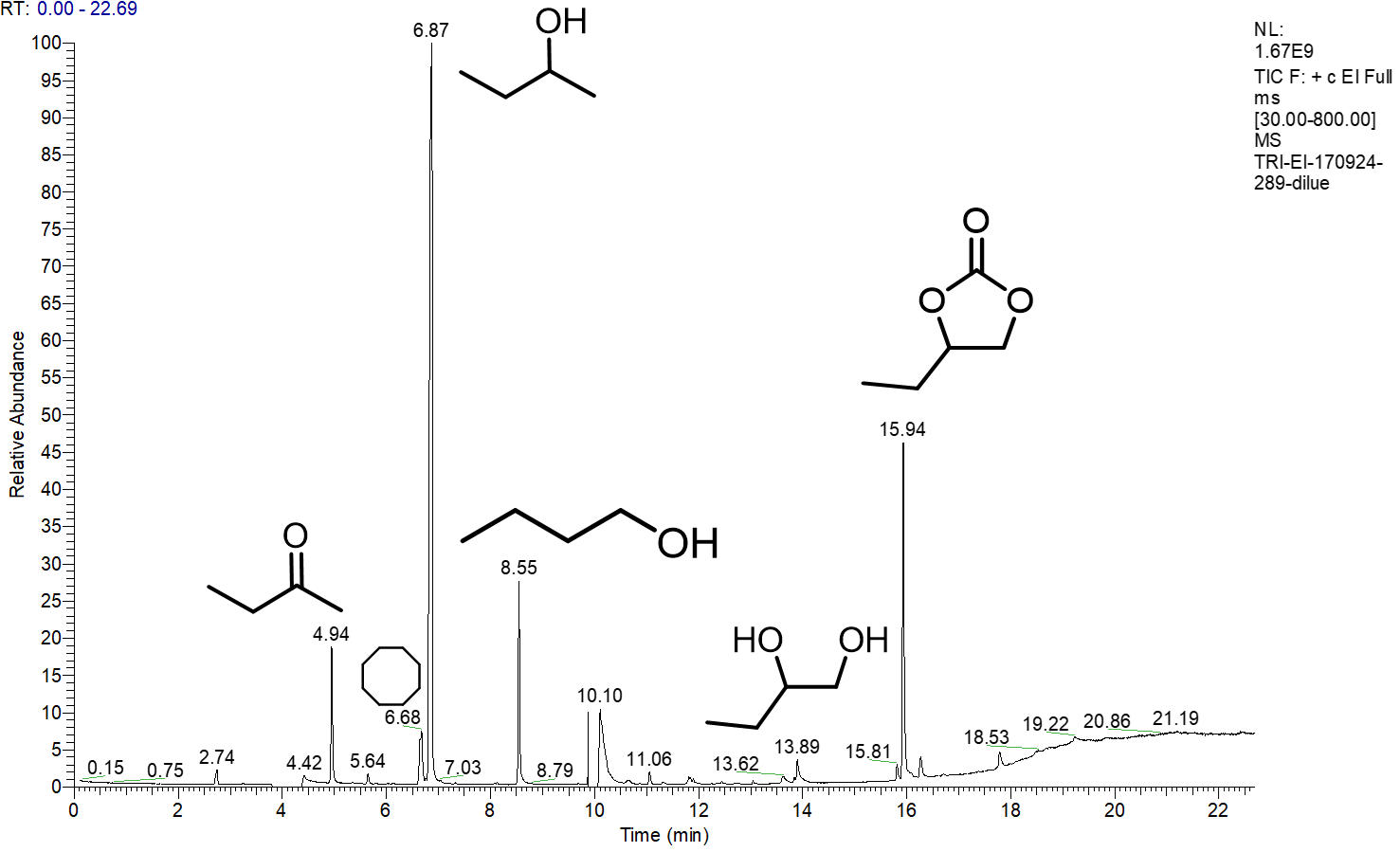
**

**Figure S22** GC-MS spectrum of the hydrogenation of BC (**1c**) by **PIL(I)** (3.5 mol%) and Ru(II) (COD)(Meallyl)_2_ (1 mol%) (Table 2, **2c**, entry 3) using mesitylene as an internal standard.

**
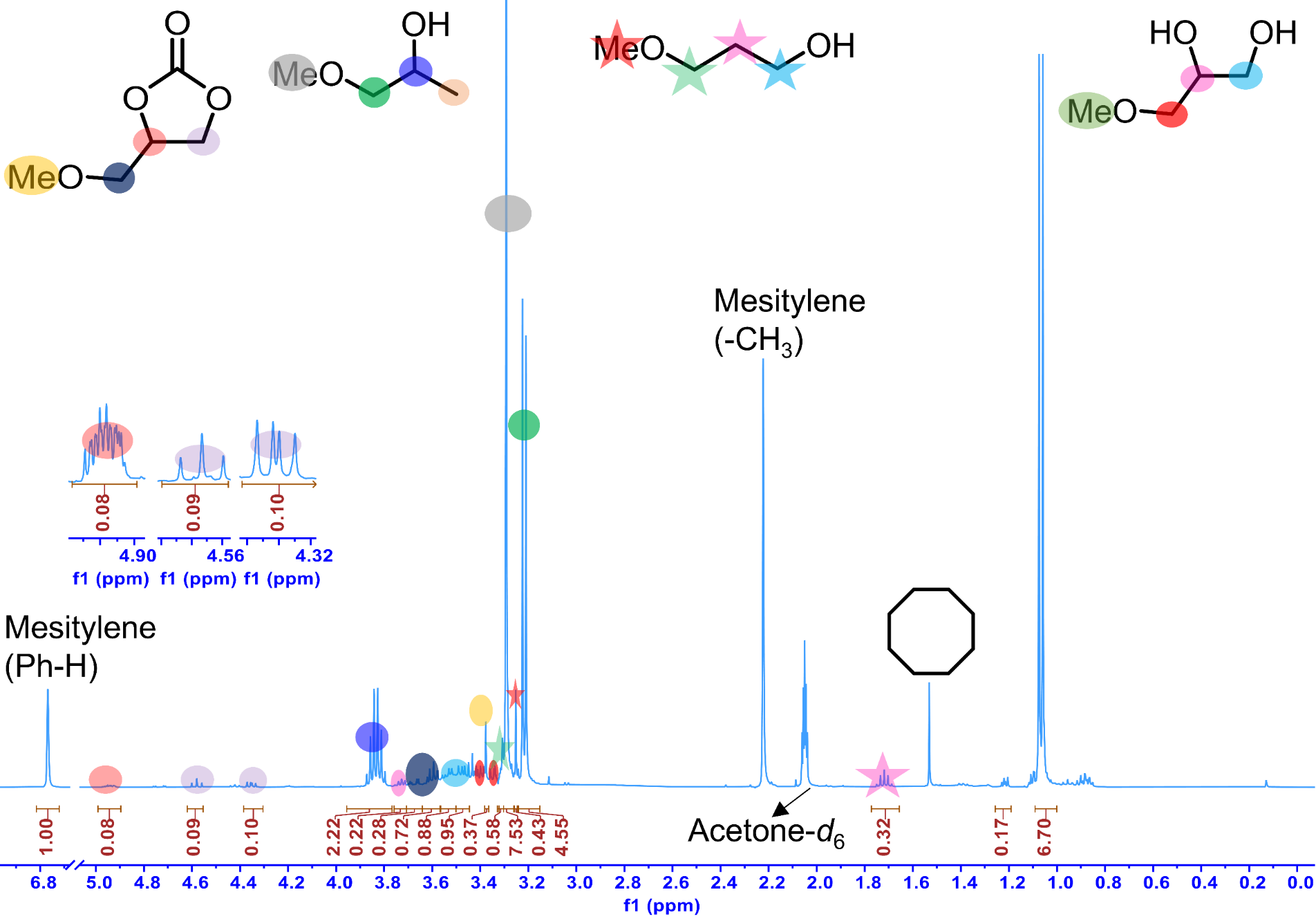
**

**Figure S23** ^1^H NMR (400 MHz, Acetone-d_6_, 25 ^o^C) spectrum of the hydrogenation of 4-(methoxymethyl)-1,3-dioxolan-2-one (**1d**) by **PIL(I)** (3.5 mol%) and Ru(II) (COD)(Meallyl)_2_ (1 mol%) (Table 2, **2d**, entry 4) using mesitylene as an internal standard.

**
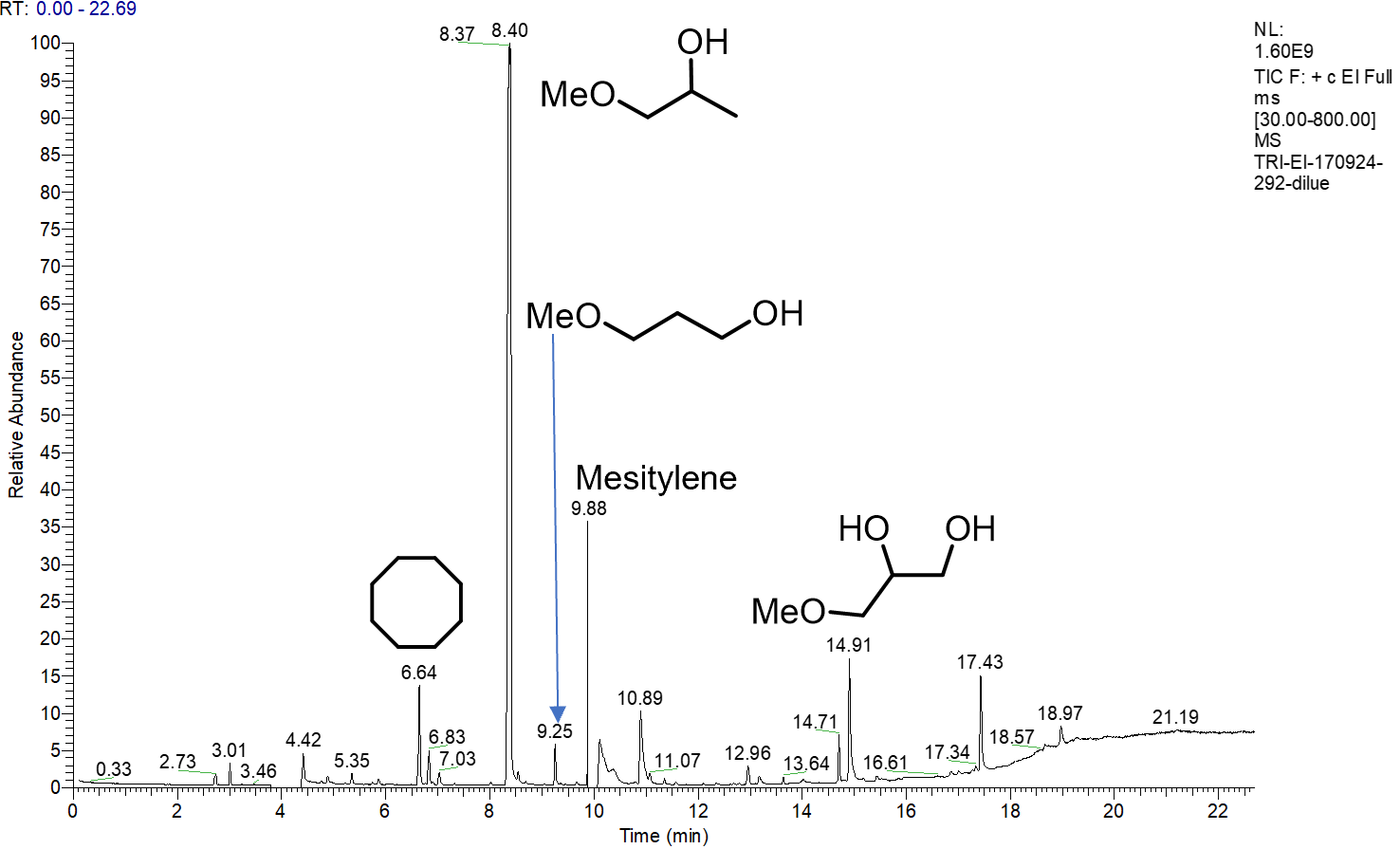
**

**Figure S24** GC-MS spectrum of the hydrogenation of 4-(methoxymethyl)-1,3-dioxolan-2-one (**1d**) by **PIL(I)** (3.5 mol%) and Ru(II) (COD)(Meallyl)_2_ (1 mol%) (Table 2, **2d**, entry 4) using mesitylene as an internal standard.

**
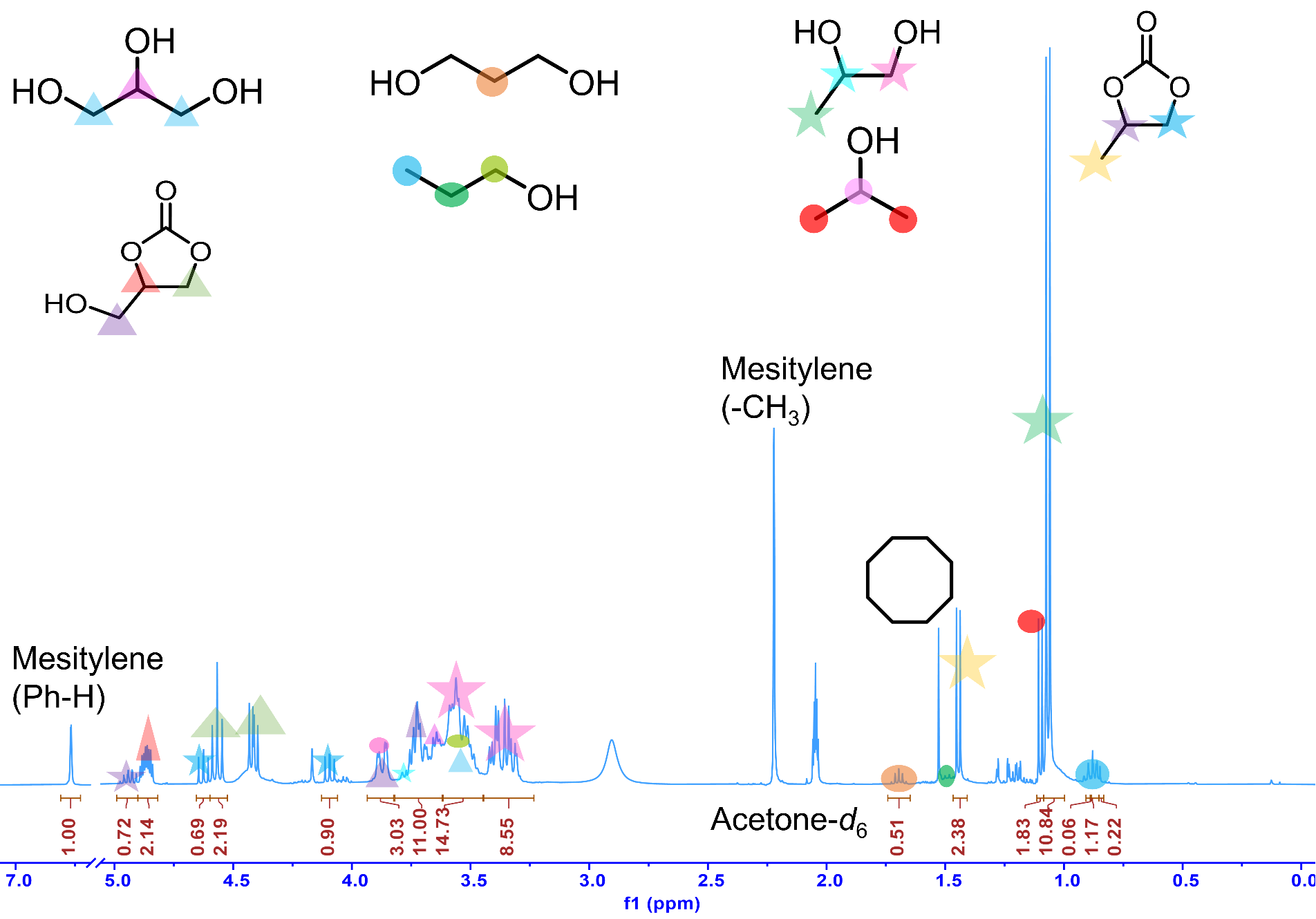
**

**Figure S25** ^1^H NMR (400 MHz, Acetone-d_6_, 25 ^o^C) spectrum of the hydrogenation of glycerol carbonate (GC, **1e**) by **PIL(I)** (3.5 mol%) and Ru(II) (COD)(Meallyl)_2_ (1 mol%) (Table 2, **2e**, entry 5) using mesitylene as an internal standard. Note that propylene carbonate could be generated from the dehydration and hydrogenation of glycerol carbonate.^12]^ Hydrogenation of the latter would propylene glycol and isopropanol.

**
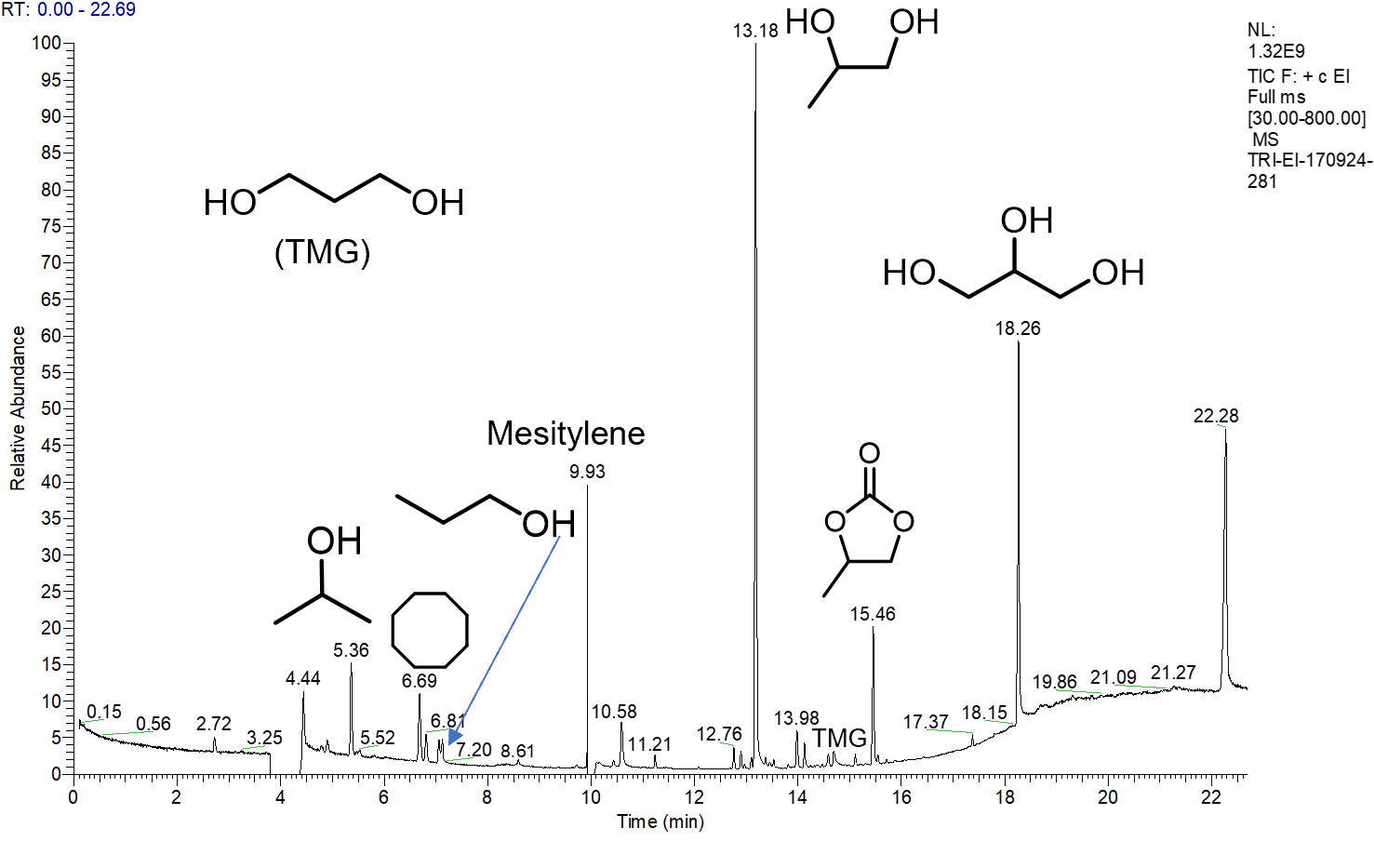
**

**Figure S26** GC-MS spectrum of the hydrogenation of glycerol carbonate ((GC, **1e**) by **PIL(I)** (3.5 mol%) and Ru(II) (COD)(Meallyl)_2_ (1 mol%) (Table 2, **2e**, entry 5) using mesitylene as an internal standard.

**
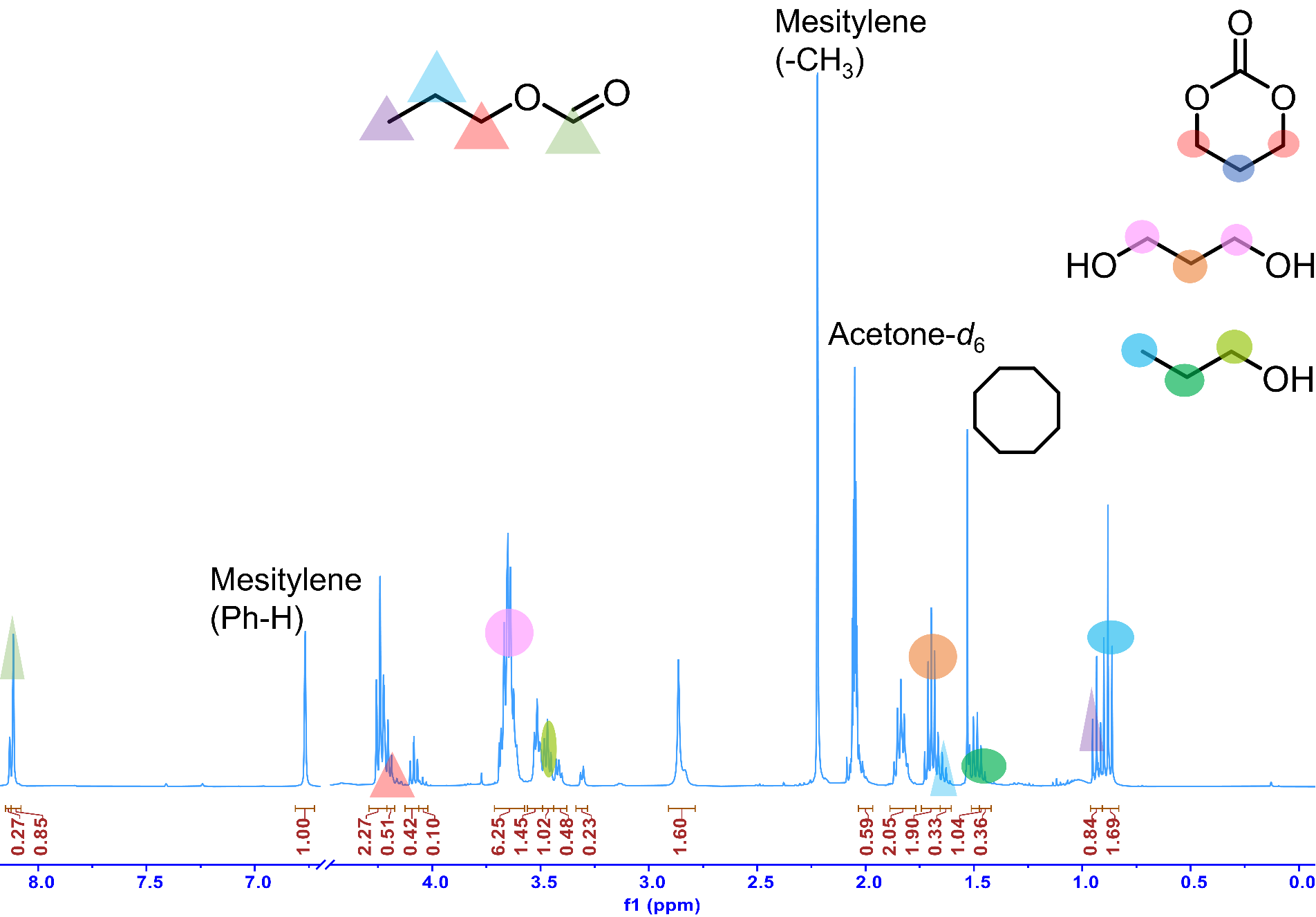
**

**Figure S27** ^1^H NMR (400 MHz, Acetone-d_6_, 25 ^o^C) spectrum of the hydrogenation of trimethylene carbonate (TMC, **1f**) by **PIL(I)** (7 mol%) and Ru(II) (COD)(Meallyl)_2_ (2 mol%) (Table 2, **2f**, entry 6) using mesitylene as an internal standard. Propyl formate could result from the reaction between 1-propanol and the formic acid formed by hydrogenation of CO_2_ or from the transesterification between 1-propanol and TMC, followed by partial hydrogenation of the carbonate moiety.

**
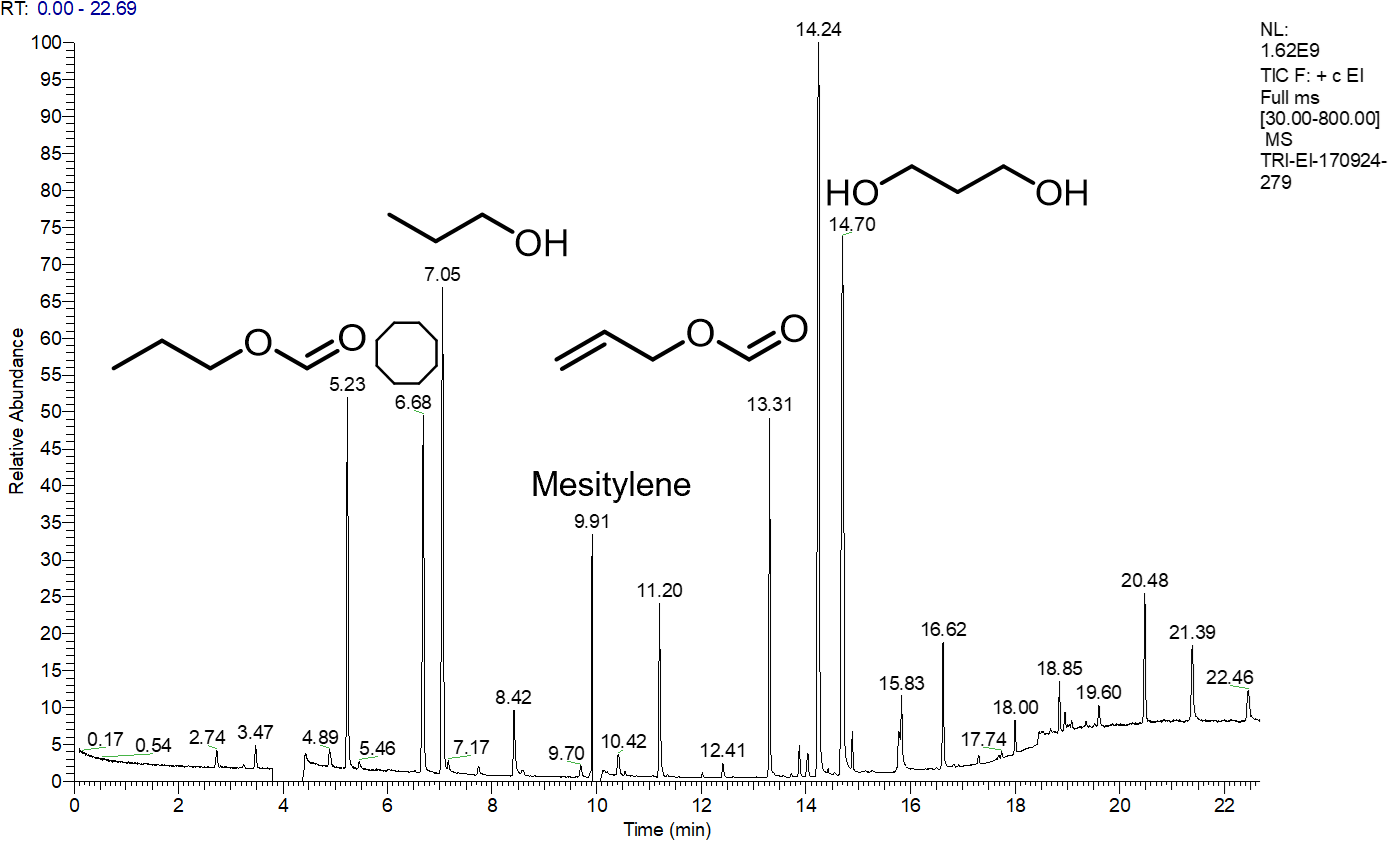
**

**Figure S28** GC-MS spectrum of the hydrogenation of trimethylene carbonate (TMC, **1f**) by **PIL(I)** (7 mol%) and Ru(II) (COD)(Meallyl)_2_ (2 mol%) (Table 2, **2f**, entry 6) using mesitylene as an internal standard. Allyl formate (13.31 min) could be produced by the dehydrogenation of propyl formate.

**^1^H NMR spectrum and GC-MS of different substrates in Table 3.**

**
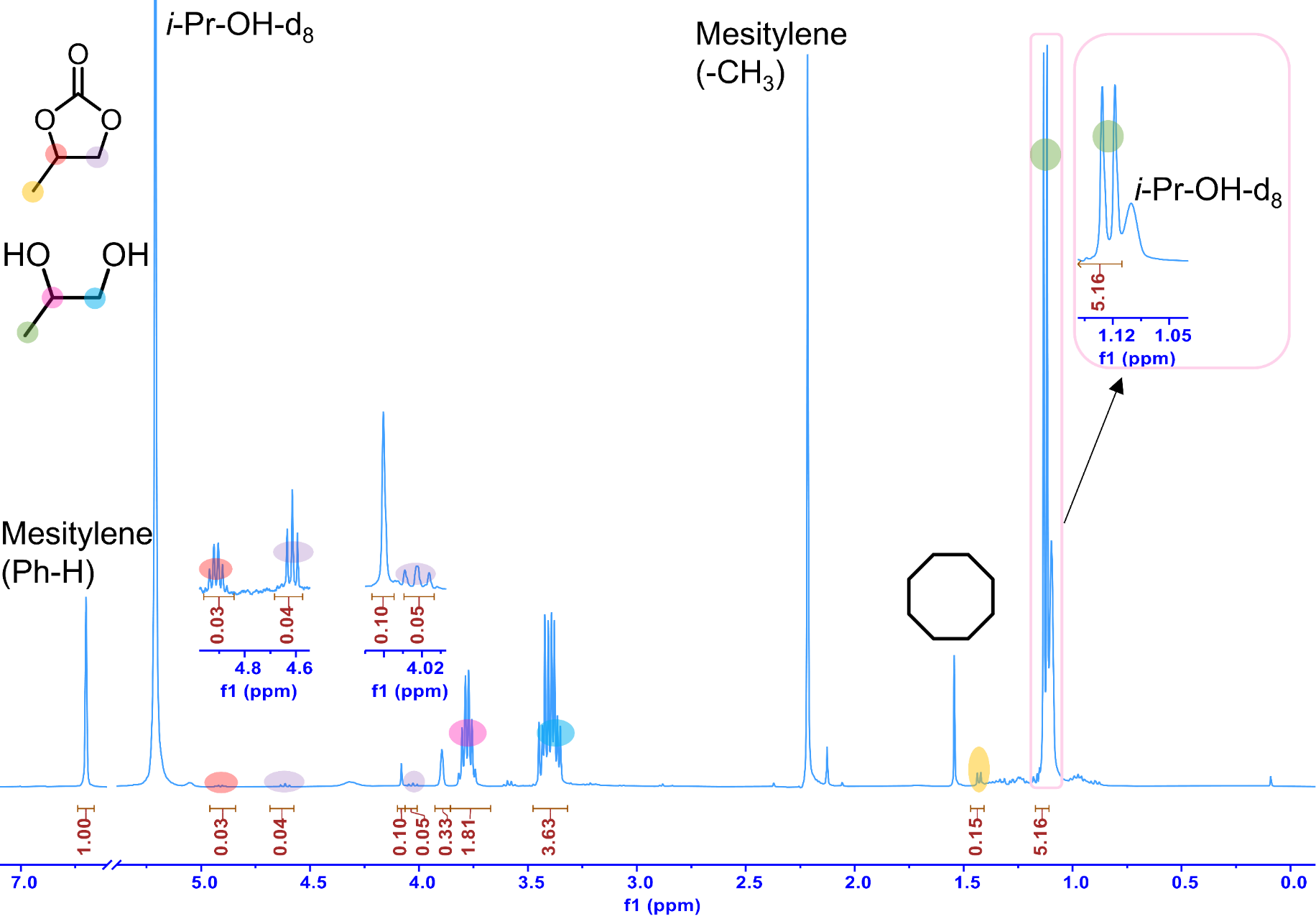
**

**Figure S29** ^1^H NMR (400 MHz, isopropanol-d_8_, 25 ^o^C) spectrum of the hydrogenation of propylene carbonate (PC, **1b**) by **PIL(NTf_2_**) (7 mol%) and Ru(II) (COD)(Meallyl)_2_ (2 mol%) (Table 3, **entry 2**) using mesitylene as an internal standard.

**
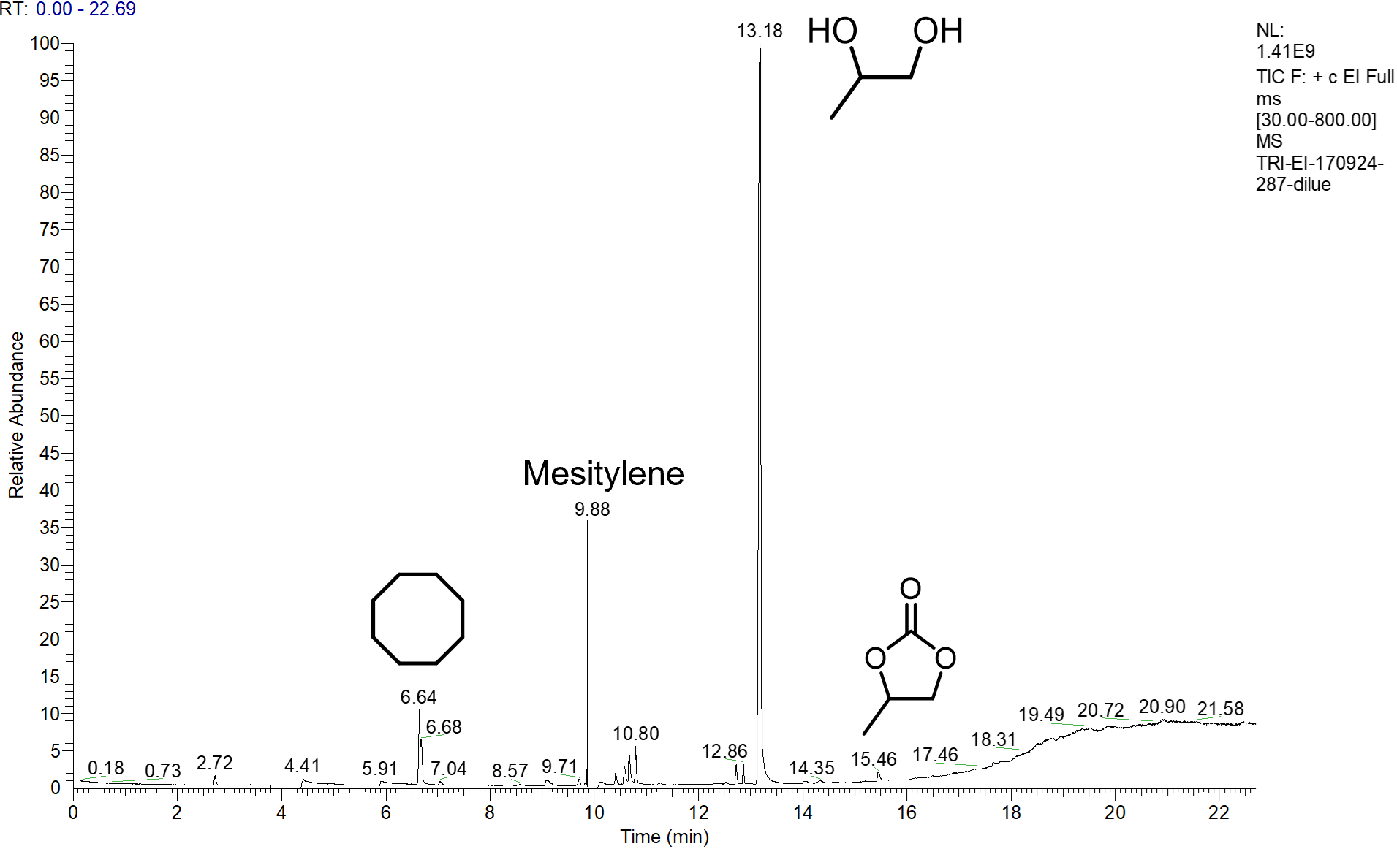
**

**Figure S30** GC-MS spectrum of the hydrogenation of propylene carbonate (PC, **1b**) by **PIL(NTf_2_**) (7 mol%) and Ru(II) (COD)(Meallyl)_2_ (2 mol%) (Table 3, **entry 2**) using mesitylene as an internal standard.

**
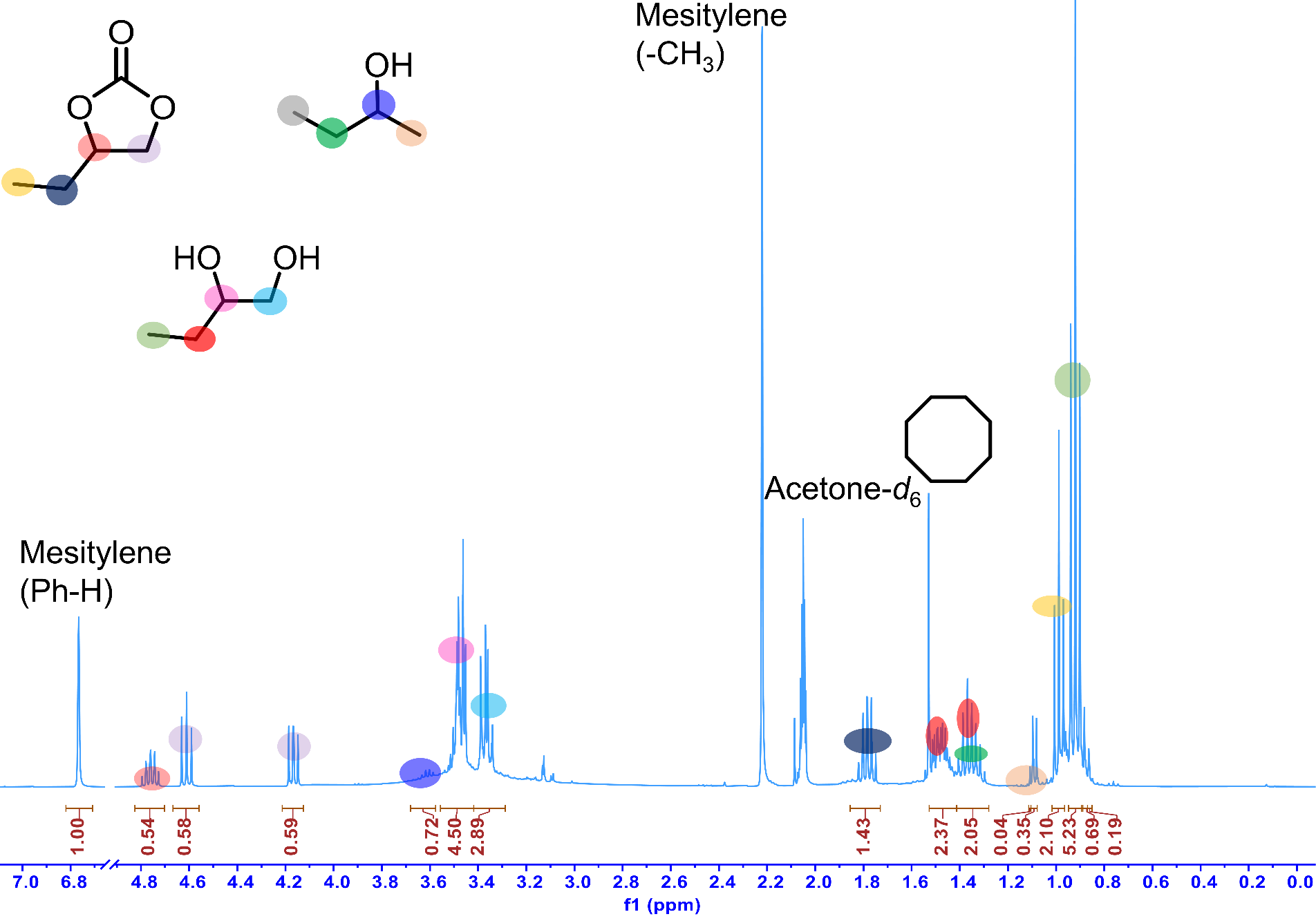
**

**Figure S31** ^1^H NMR (400 MHz, Acetone-d_6_, 25 ^o^C) spectrum of the hydrogenation of butylene carbonate (BC, **1c**) by **PIL(NTf_2_**) (7 mol%) and Ru(II) (COD)(Meallyl)_2_ (2 mol%) (Table 3, **entry 3**) using mesitylene as an internal standard.

**
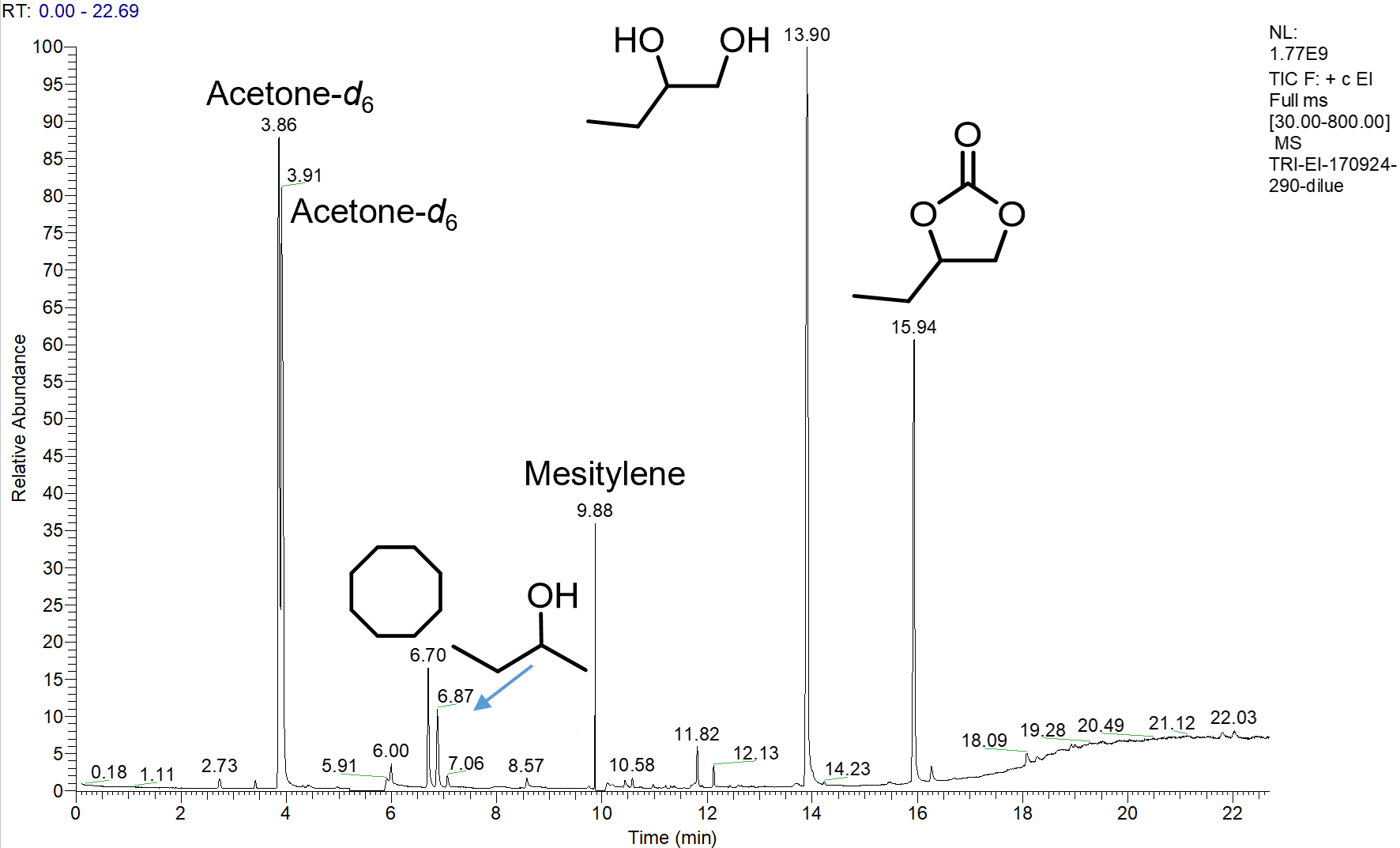
**

**Figure S32** GC-MS spectrum of the hydrogenation of butylene carbonate (BC, **1c**) by **PIL(NTf_2_**) (7 mol%) and Ru(II) (COD)(Meallyl)_2_ (2 mol%) (Table 3, **entry 3**) using mesitylene as an internal standard.

**
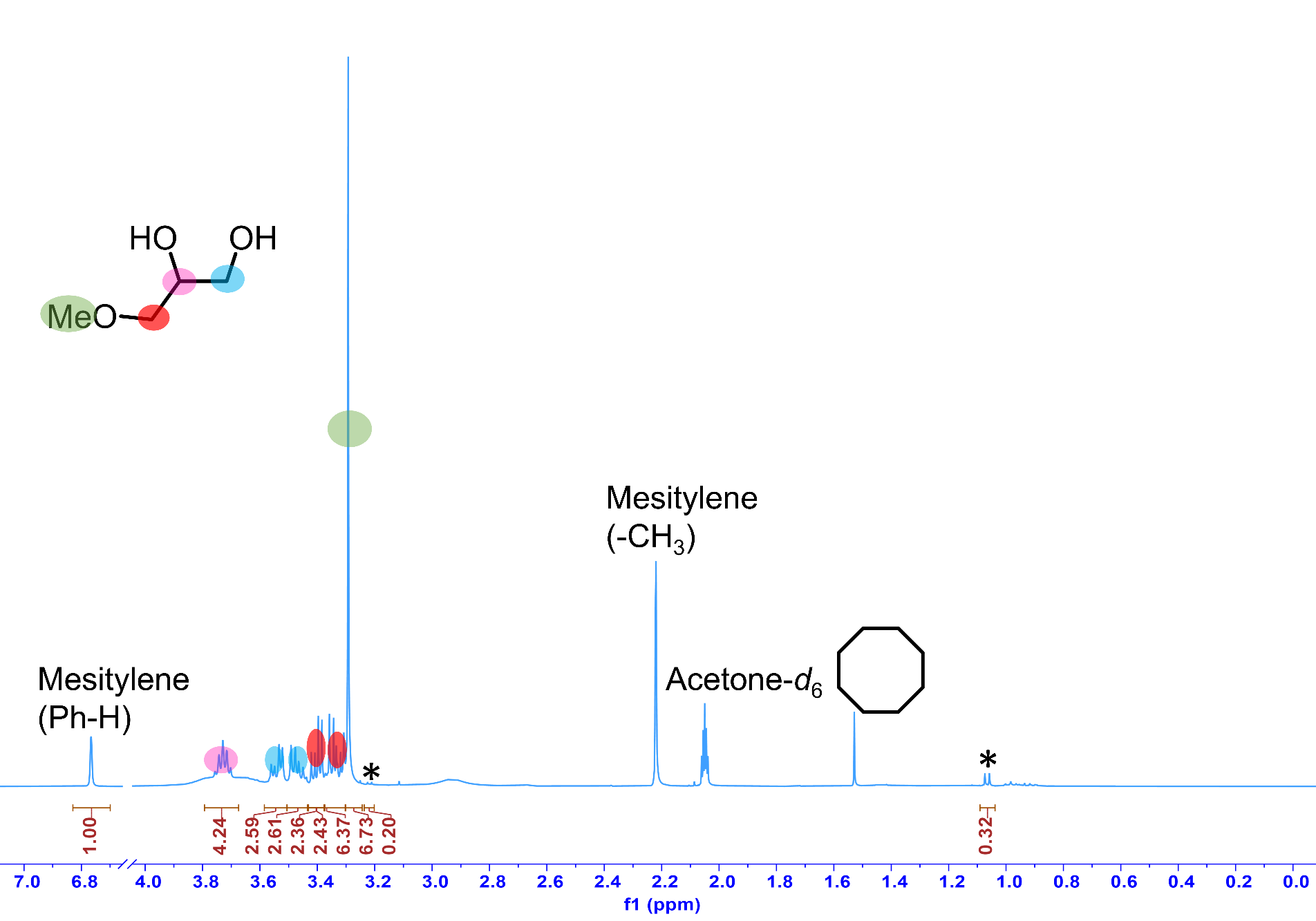
**

**Figure S33** ^1^H NMR (400 MHz, Acetone-d_6_, 25 ^o^C) spectrum of the hydrogenation of 4-(methoxymethyl)-1,3-dioxolan-2-one (**1d**) by **PIL(NTf_2_**) (7 mol%) and Ru(II) (COD)(Meallyl)_2_ (2 mol%) (Table 3, **3d**, entry 4) using mesitylene as an internal standard. The signals at 1.1 and 3.2 ppm belong to the byproduct 1-methoxypropan-2-ol*.

**
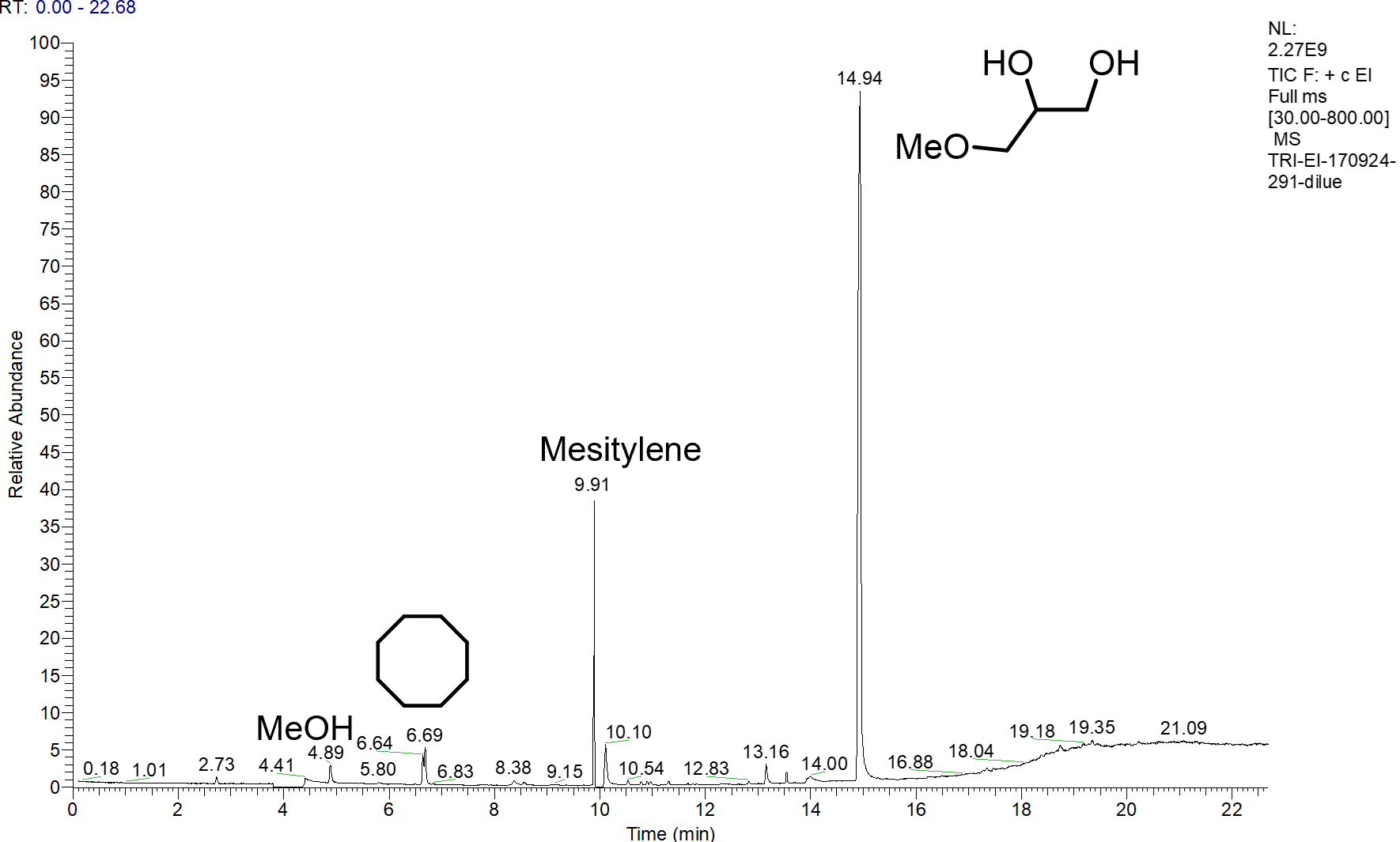
**

**Figure S34** GC-MS spectrum of the hydrogenation of 4-(methoxymethyl)-1,3-dioxolan-2-one (**1d**) by **PIL(NTf_2_**) (7 mol%) and Ru(II) (COD)(Meallyl)_2_ (2 mol%) (Table 3, **3d**, entry 4) using mesitylene as an internal standard.

**
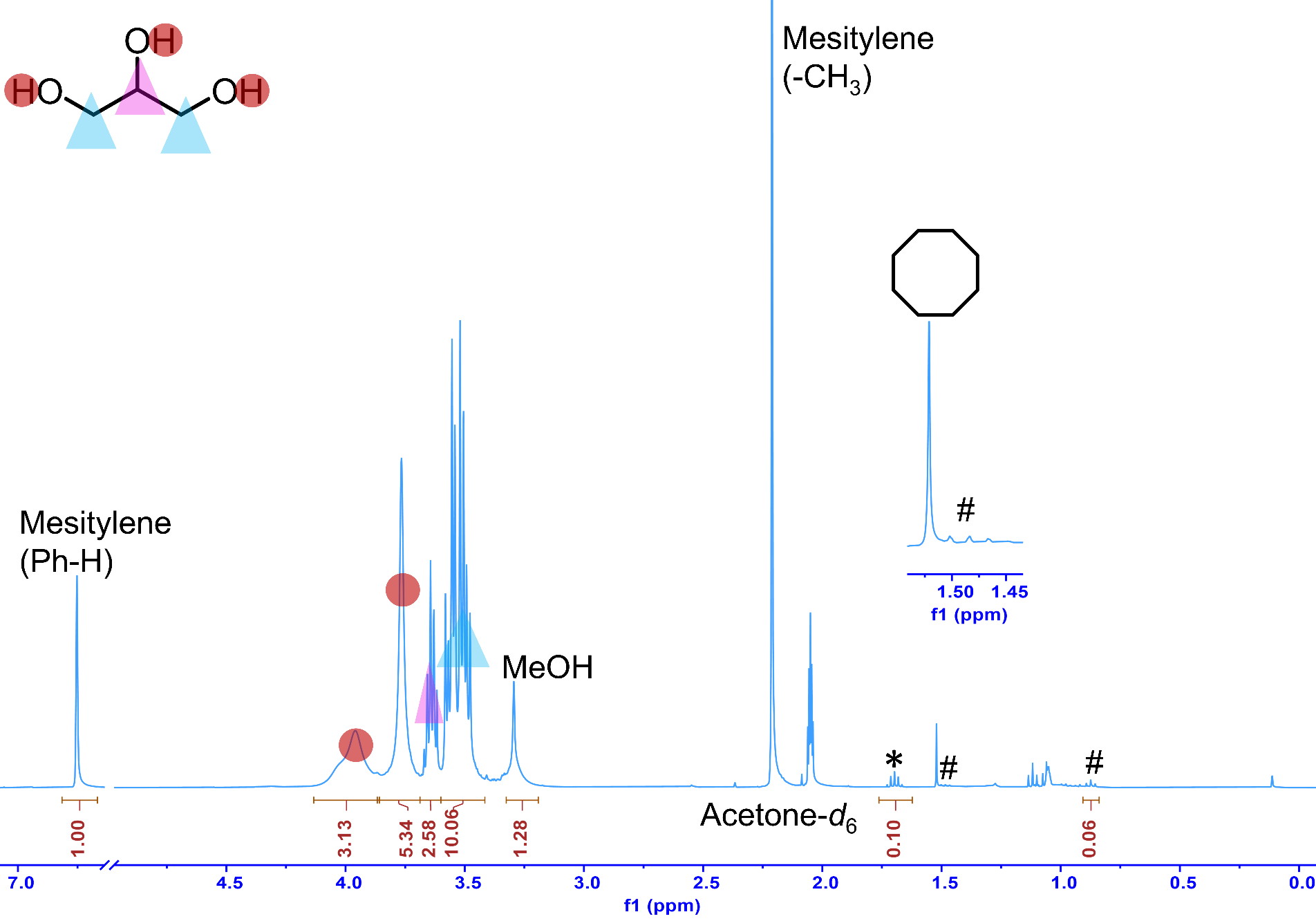
**

**Figure S35** ^1^H NMR (400 MHz, Acetone-d_6_, 25 ^o^C) spectrum of the hydrogenation of glycerol carbonate (GC, **1e**) by **PIL(NTf_2_**) (7 mol%) and Ru(II) (COD)(Meallyl)_2_ (2 mol%) (Table 3, **3e**, entry 5) using mesitylene as an internal standard. The signals at 0.90 and 1.53 ppm belong to the byproduct 1-propanol^#^. The signal at 1.75 ppm corresponds to the byproduct trimethyl glycol (TMG)^*^.

**
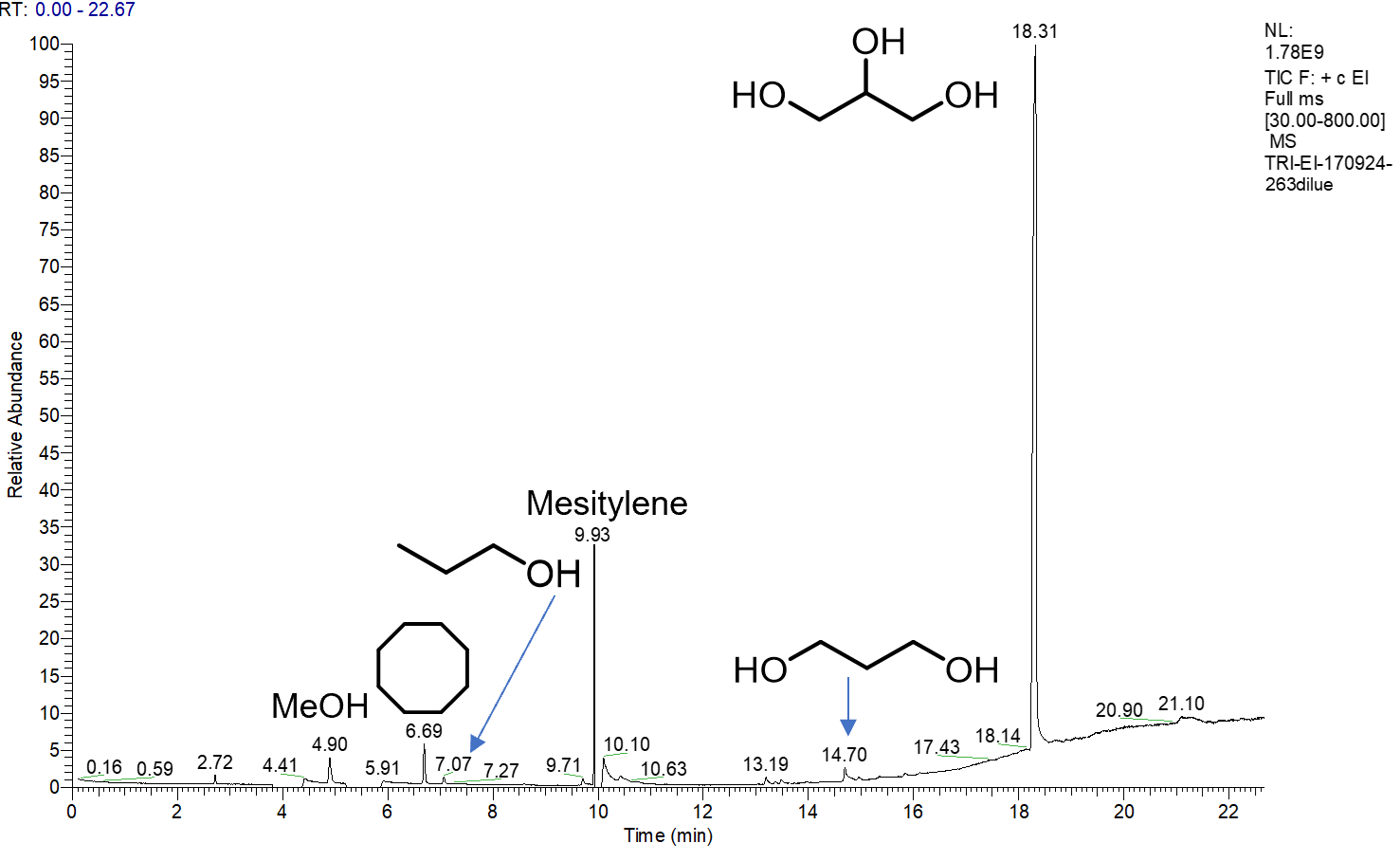
**

**Figure S36** GC-MS spectrum of the hydrogenation of glycerol carbonate (GC, **1e**) by **PIL(NTf_2_**) (7 mol%) and Ru(II) (COD)(Meallyl)_2_ (2 mol%) (Table 3, **3e**, entry 5) using mesitylene as an internal standard.

**
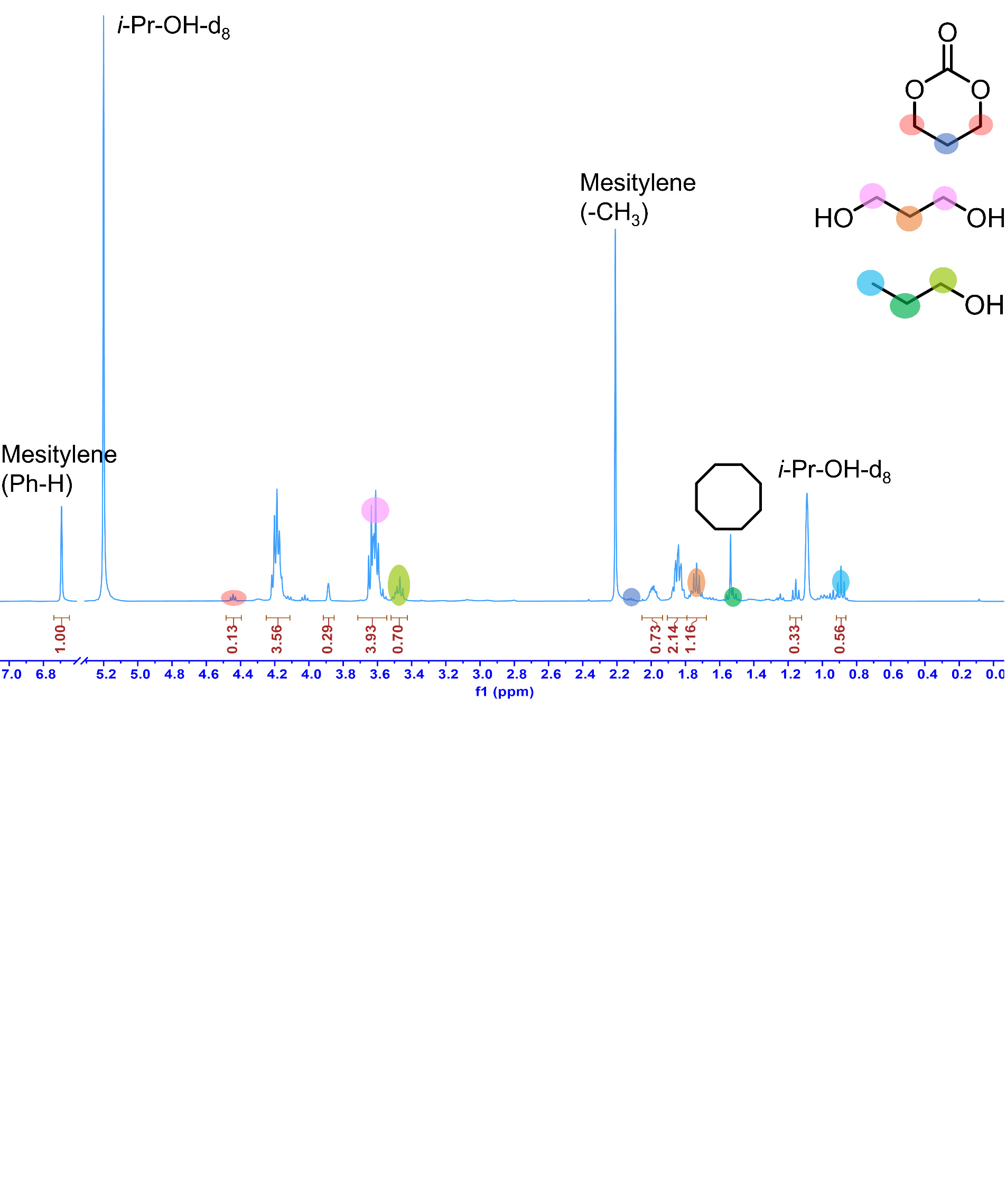
**

**Figure S37** ^1^H NMR (400 MHz, Acetone-d_6_, 25 ^o^C) spectrum of the hydrogenation of trimethylene carbonate (TMC, **1f**) by **PIL(NTf_2_**) (7 mol%) and Ru(II) (COD)(Meallyl)_2_ (2 mol%) (Table 3, **3f**, entry 6) using mesitylene as an internal standard.

**
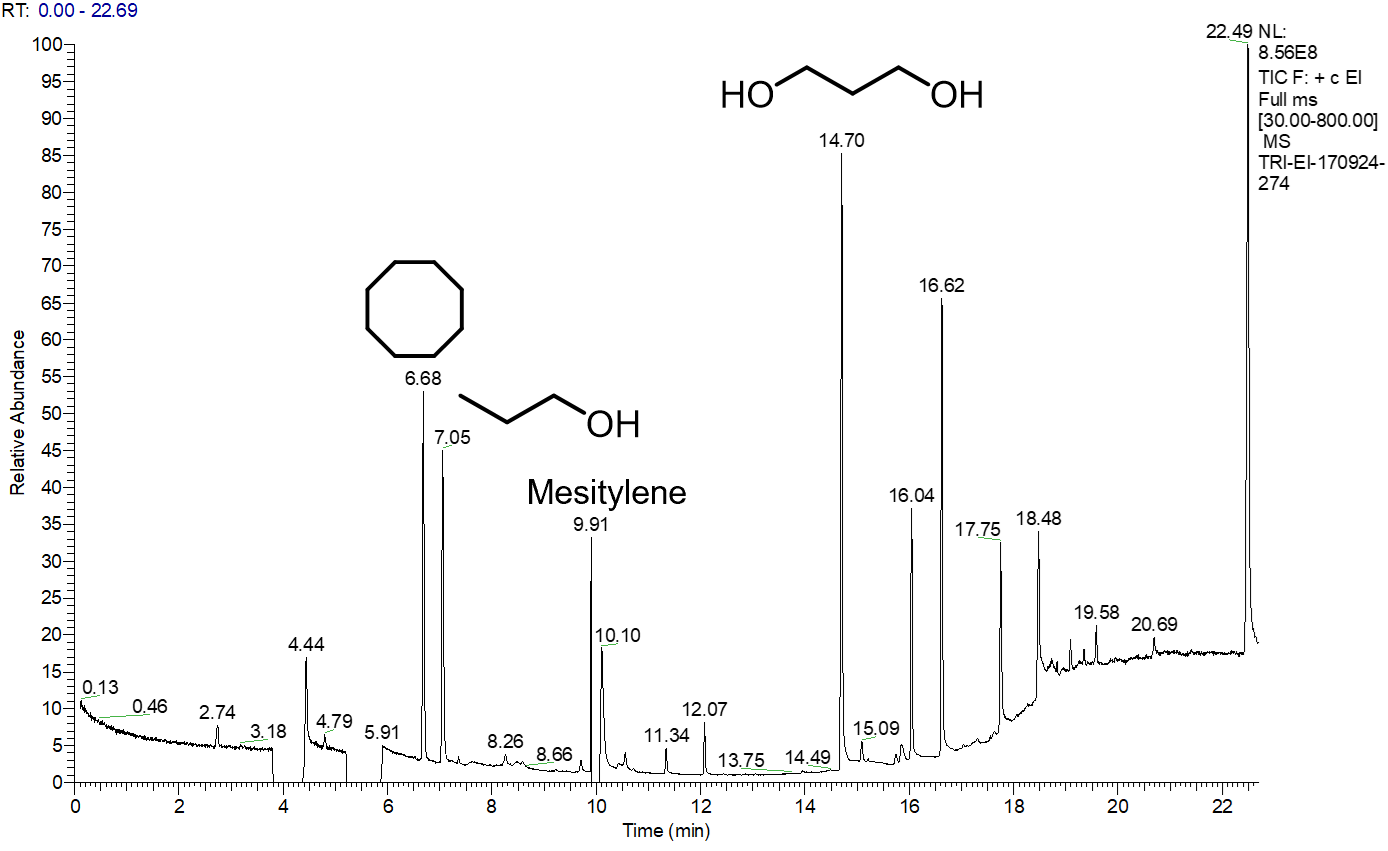
**

**Figure S38** GC-MS spectrum of the hydrogenation of trimethylene carbonate (TMC, **1f**) by **PIL(NTf_2_**) (7 mol%) and Ru(II) (COD)(Meallyl)_2_ (2 mol%) (Table 3, **3f**, entry 6) using mesitylene as an internal standard.

**
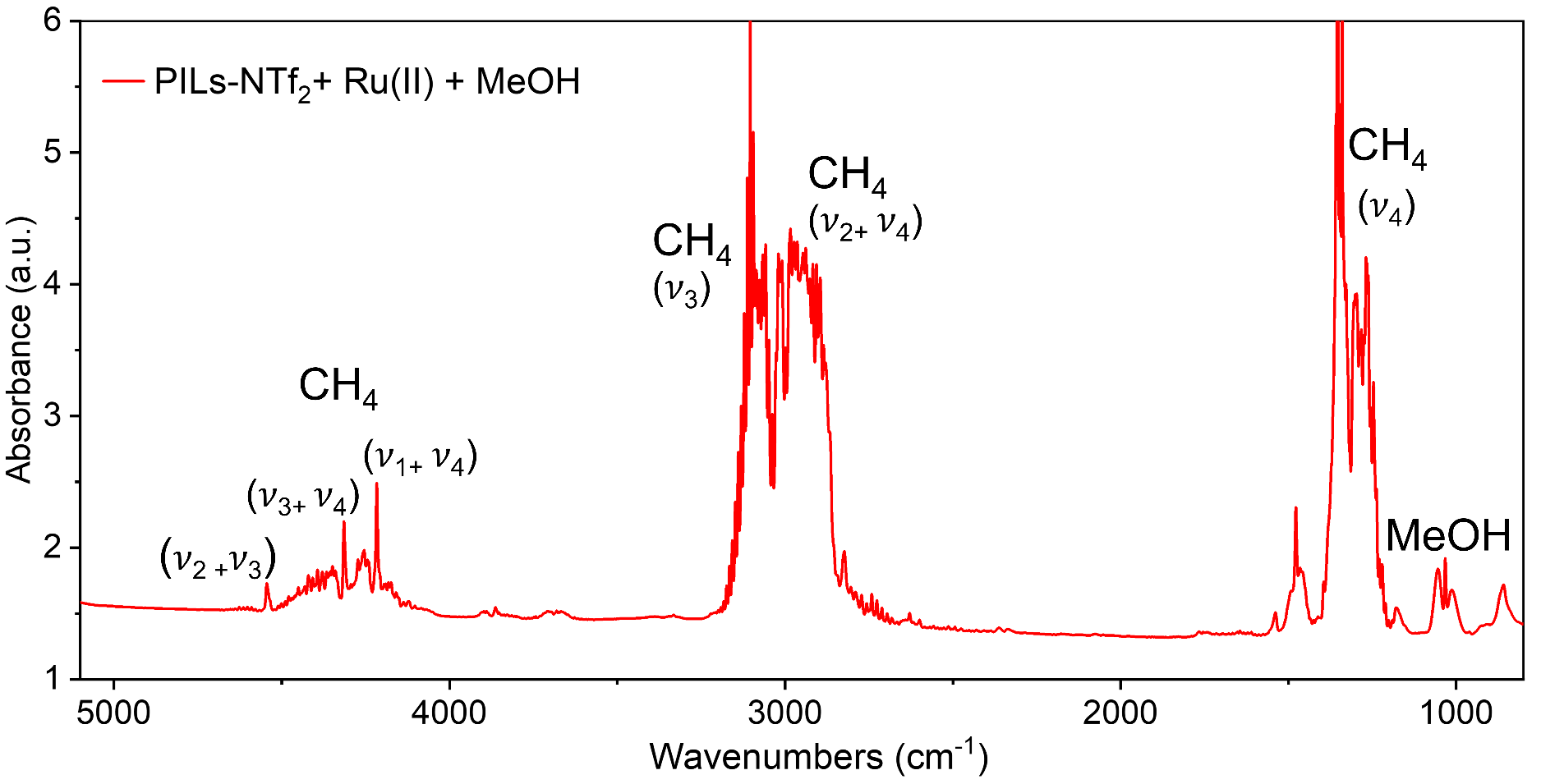
**

**Figure S39** FT-IR spectrum of the gas phase of the hydrogenation of MeOH by **PIL(NTf_2_**) (7 mol%) and Ru(II) (COD)(Meallyl)_2_ (2 mol%) (Table 3, **entry 7**) using mesitylene as an internal standard.

**References**

1. a) N. Chakroune, G. Viau, S. Ammar, L. Poul, D. Veautier, M. M. Chehimi, C. Mangeney, F. Villain, F. Fiévet, *Langmuir* **2005**, 21, 6788-6796; b) T. Cremer, C. Kolbeck, K. R. J. Lovelock, N. Paape, R. Wölfel, P. S. Schulz, P. Wasserscheid, H. Weber, J. Thar, B. Kirchner, F. Maier, H.-P. Steinrück, *Chem. Eur. J.* **2010**, *16*, 9018-9033.
2. a) J. Yang, J. Y. Lee, T. C. Deivaraj, H.-P. Too, *J. Colloid Interface Sci.* **2004**, *271*, 308-312; b) M. Zawadzki, J. Okal, *Mater. Res. Bull.* **2008**, *43*, 3111-3121; c) J. L. Gómez de la Fuente, M. V. Martínez-Huerta, S. Rojas, P. Hernández-Fernández, P. Terreros, J. L. G. Fierro, M. A. Peña, *Appl. Catal., B: Environmental* **2009**, *88*, 505-514; d) J.F. Moulder, W.F. Stickle, P.E. Sobol, K.D. Bomben, Handbook of X ray Photoelectron Spectroscopy, Physical Electronics **1995**.
3. NIST X-ray Photoelectron Spectroscopy Database, NIST Standard Reference Database Number 20, National Institute of Standards and Technology, Gaithersburg MD 2000, 20899 (retrieved 18/01/2022).
4. H. He, M. Zhong, B. Adzima, D. Luebke, H. Nulwala and K. Matyjaszewski, *J. Am. Chem. Soc.* **2013**, *135*, 4227-4230.
5. D. Parida, C. Bakkali-Hassani, E. Lebraud, C. Schatz, S. Grelier, D. Taton, J. Vignolle, *Nanoscale* **2022**, *14*, 4635–4643.
6. a) R. Marcilla, J. B. Alberto, J. Rodriguez, J. A. Pomposo and D. Mecerreyes, *J. Polym. Sci., Part A: Polym. Chem.* **2004**, *42*, 208-212; b) Y. Rong, Z. Ku, M. Xu, L. Liu, M. Hu, Y. Yang, J. Chen, A. Mei, T. Liu, H. Han, *RSC Adv.* **2014**, *4*, 9271-9274; c) R. Thenarukandiyil, H. Thrikkykkal, J. Choudhury, *Organometallics* **2016**, *35*, 3007-3013.
7. a) M. Ohtaki, M. Komiyama, H. Hirai, N. Toshima, *Macromolecules,* **1991**, *24*, 5567-5572; b) L. S. Sarma, C.-H. Chen, S. M. S. Kumar, G.-R. Wang, S.-C. Yen, D.-G. Liu, H.-S. Sheu, K.-L. Yu, M.-T. Tang, J.-F. Lee, C. Bock, K.-H. Chen, B.-J. Hwang, *Langmuir* **2007**, *23*, 5802-5809.
8. M. Buback, J. Schweer, H. Yups, Z. Naturforschung **1986**, *41a*, 505-511.
9. G. Boe, J.L. Bruneel, T. Tassaing, *Appl Spectrosc* **2024**, *78*, 760-771.
10. Ó. Gálvez, B. Maté, V. J. Herrero, R. Escribano, *Astrophys. J.*, **2009**, 703, 2101-2107.
11. a) Z. Han, L. Rong, J. Wu, L. Zhang, Z. Wang, K. Din, *Angew. Chem. Int. Ed.* **2012**, *51*, 13041 –13045; b) S. H. Kim, S. H. Hong, *ACS Catal.* **2014**, **4**, 3630-3636.
12. S. Wang, Y. Zhang, H. Liu, *Chem. Asian J.*, **2010**, 5, 1100-1111.
